# Ultra-rapid lispro or fast-acting aspart compared to standard insulin lispro and aspart using closed-loop insulin therapy: a systematic review and meta-analysis of randomized control trials

**DOI:** 10.3389/fendo.2025.1600157

**Published:** 2025-06-06

**Authors:** Mohamed Saad Rakab, Rahma Mogahed Rateb, Basel Hatem Elsalakawi, Bashar M. Al Zoubi, Abubakar Nazir, Mutaz Moath Abu-Laila, Abdelrahman Sherif Ghanem, Alaa Maamoun, Mohab Mattar, Basma Ataallah, Brian T. Layden, Abeer M. Mahmoud

**Affiliations:** ^1^ Faculty of Medicine, Mansoura University, Mansoura, Egypt; ^2^ Faculty of Medicine, Assiut University, Assiut, Egypt; ^3^ Faculty of Medicine, Hashemite University, Zarqa, Jordan; ^4^ College of Medicine, King Edward Medical University, Lahore, Pakistan; ^5^ Faculty of Medicine, Yarmouk University, Irbid, Jordan; ^6^ Faculty of Medicine, Zagazig University, Zagazig, Egypt; ^7^ Department of Medicine, Houston Methodist/Willowbrook Hospital, Houston, TX, United States; ^8^ Department of Medicine, Division of Endocrinology, Diabetes, and Metabolism, College of Medicine, University of Illinois Chicago, Chicago, IL, United States; ^9^ Department of Medicine, Jesse Brown Veterans Affairs Medical Center, Chicago, IL, United States; ^10^ Department of Kinesiology and Nutrition, College of Applied Health Sciences, University of Illinois Chicago, Chicago, IL, United States

**Keywords:** closed-loop systems, ultra fast acting insulin analogs, type 1 diabetes, glucose variability, meta-analysis

## Abstract

**Background:**

Ultra-rapid-acting insulin (URAI) improves glycemic control by reducing variability; however, optimal strategies for its use, especially within hybrid closed-loop (HCL) insulin delivery systems, remain unclear. This meta-analysis assesses the efficacy and safety of combining URAI with HCL systems in maintaining the euglycemic range and reducing glycemic excursions.

**Methods:**

We systematically searched PubMed, Scopus, Cochrane Library, Web of Science, and related article citations for relevant studies. Outcomes assessed included time in range (TIR), time below range (TBR), and time above range (TAR) during overall 24-hour periods, daytime, nighttime, postprandial, and post-exercise periods, as well as adverse events. Dichotomous outcomes were summarized using risk ratios (RR), and continuous outcomes were pooled using mean differences (MD) presented with 95% confidence intervals (CI).

**Results:**

URAI showed a modest, statistically non-significant improvement in TIR (70–180 mg/dL) compared to standard insulin (MD 0.87%, 95% CI [-0.21 to 1.85], P = 0.12). Importantly, glycemic variability significantly improved with URAI, as demonstrated by reductions in the coefficient of variation (CV) (MD -0.78%, 95% CI [-1.44 to -0.12], P = 0.02). The combination of URAI with HCL systems significantly reduced hypoglycemia (TBR <70 mg/dL: MD -0.32%, 95% CI [-0.56 to -0.13], P = 0.002). However, overall reductions in TAR >250 mg/dL and TAR >180 mg/dL were statistically non-significant.

**Conclusion:**

The integration of URAI with HCL demonstrates encouraging improvements in glycemic outcomes, notably reduced glucose variability and nighttime hypoglycemia risk. However, further research with larger sample sizes is essential to confirm these benefits and establish broader clinical recommendations.

**Systematic review registration:**

https://www.crd.york.ac.uk/PROSPERO/view/CRD42024594375, identifier CRD42024594375.

## 1 Introduction

The 21st century has seen an increase in the prevalence of diabetes, which has become a global public health concern. Once common in Western nations, diabetes has now struck globally, driven by widespread consumption of calorie-dense, nutrient-poor diets combined and increasingly sedentary lifestyles (1). According to the Global Burden of Diseases, Injuries, and Risk Factors Study (GBD) 2019, approximately 460 million individuals across all age groups were affected by diabetes, according to estimates from the Global Burden of Diseases, Injuries, and Risk Factors Study (GBD) 2019, ranking it as the seventh-leading cause of death and disability globally (2).

Recent technological advancements have significantly transformed diabetes management, notably through the development of automated insulin delivery (AID) systems designed to reduce disease burden and enhance glycemic control for individuals with type 1 diabetes (3). Among these innovations is the hybrid closed-loop (HCL) system, commonly referred to as an “artificial pancreas,” which integrates an insulin pump, a continuous glucose monitor (CGM), and a sophisticated algorithm running on a computer or smartphone (4). HCL systems, often integrated into AID devices, automatically determine and administer basal insulin doses, whereas mealtime insulin boluses require manual input regarding meal size and timing (5). Currently, these insulin administration systems represent the pinnacle of insulin delivery technology available for type 1 diabetes management, significantly improving glucose control and lowering hypoglycemia risk (6, 7). Commercially available HCL systems, such as the Medtronic 670G/780G, Tandem t:slim X2 Control IQ, and CamAPS FX systems, have obtained regulatory approval from the United States Food and Drug Administration (FDA) and European Conformity (CE) certification, supported by robust clinical trial data (8, 9).

Rapid-Acting Insulin Analogues (RAIAs) like aspart, lispro, and glulisine exhibit faster absorption kinetics compared to regular human insulin, yet managing optimal post-prandial glucose (PPG) remains challenging (10, 11). Consequently, newer ultra-rapid-acting insulins (URAIs) have been developed, including faster-acting insulin aspart (FIAsp; marketed as Fiasp, approved in 2017) and ultra-rapid lispro insulin (URLi; marketed as Lyumjev, approved in 2020) (12, 13).

Previous randomized controlled trials (RCTs) evaluating glycemic control with Fiasp and URLi in HCL systems have produced mixed results compared to conventional RAIAs (14–16). To address these inconsistencies, this systematic review and meta-analysis aim to comprehensively assess the effectiveness and safety of Fiasp and URLi within HCL systems among patients with type 1 diabetes.

## 2 Methods

### 2.1 Protocol registration

Our systematic review protocol was registered in PROSPERO (registration ID: CRD42024594375). This systematic review and meta-analysis were conducted following the guidelines outlined in the Preferred Reporting Items for Systematic Reviews and Meta-Analyses (PRISMA) statement and the Cochrane Handbook for Systematic Reviews and Meta-Analysis (17, 18).

### 2.2 Data sources & search strategy

We systematically searched the Web of Science, SCOPUS, PubMed (MEDLINE), and Cochrane Central Register of Controlled Trials (CENTRAL) databases from their inception through December 2024 without applying any search filters. The detailed approach and results are outlined in **Supplementary Table 1**.

### 2.3 Eligibility criteria

We included RCTs based on the following PICO criteria: Patients were individuals diagnosed with type 1 diabetes; interventions involved automated insulin delivery systems using URAIs (Lispro or Aspart); comparators were automated insulin delivery systems using standard insulin (Lispro or Aspart); outcomes focused on CGM data, specifically the time in range (TIR).

Studies were excluded if they met any of the following criteria (1): non-human or *in vitro* studies; (2) overlapping or duplicate datasets; (3) book chapters, reviews, commentaries, letters to the editor, or clinical guidelines; and (4) publications not available in English.

### 2.4 Study selection

Search results from all the databases were imported to Rayyan (19), and duplicates were manually removed. Four authors (A.N., M.M.A., A.S.G., and A.M.) independently screened the remaining articles, with disagreements resolved by a fifth reviewer (M.S.R.). The screening process consisted of two stages: initial assessment of titles and abstracts to identify relevant studies, followed by full-text screening to confirm eligibility according to predefined inclusion criteria for subsequent qualitative and quantitative analyses.

### 2.5 Data extraction

Data extraction was independently performed by four reviewers (A.N., M.M.A., A.S.G., and A.M.) using a standardized Excel template. Extracted information included study characteristics (study design, country, number of centers, study setting, total participants, population details, intervention type, comparators, and follow-up duration), baseline patient data (group sample sizes, age, BMI, sex, diabetes duration, HbA1c levels, and total daily insulin doses), and clinical outcomes (time in range [70–180 mg/dL and 70–140 mg/dL], time below range [<70 mg/dL and <54 mg/dL], time above range [>180 mg/dL and >250 mg/dL], total daily insulin dose, basal insulin dose, bolus insulin dose, coefficient of variation, standard deviation of sensor glucose, mean sensor glucose, severe hypoglycemia events, diabetic ketoacidosis incidents, and infusion site reactions).

The primary outcome of this meta-analysis was time in range (70–180 mg/dL), as assessed by continuous glucose monitoring (CGM) data. Secondary outcomes included time in range (70–140 mg/dL), time below range at thresholds <70 mg/dL and <54 mg/dL, and time above range at thresholds >180 mg/dL and >250 mg/dL. Additional glycemic control measures included mean glucose, glucose standard deviation (SD), and coefficient of variation (CV). We also assessed total daily insulin dose (TDD) and the frequency of adverse events (hypoglycemia events, diabetic ketoacidosis incidents, infusion site reactions, and device-related adverse events) to evaluate treatment safety. Any disagreements among reviewers were resolved through consensus discussions.

### 2.6 Risk of bias and certainty of evidence

Four reviewers (A.N., M.M.A., A.S.G., and A.M.) independently evaluated the methodological quality of included studies using the Cochrane Risk of Bias 2 (ROB2) tool (20). Assessments covered potential biases attributed to the randomization process, deviations from intended interventions, missing outcomes, measuring outcomes, and selective reporting of results. Each outcome was assessed individually, with all decisions clearly justified and documented. Any discrepancies between reviewers were resolved through discussion and consensus. To appraise the quality of evidence, we utilized the Grading of Recommendations Assessment, Development, and Evaluation (GRADE) guidelines (21, 22). Any discrepancies were settled through discussion.

### 2.7 Statistical analysis

Statistical analyses were performed using RevMan version 4.5.1 software (23). Study results were pooled using risk ratios (RR) for dichotomous outcomes and mean differences (MD) for continuous outcomes, both presented with 95% confidence intervals (CI). A random-effects model was utilized when significant heterogeneity was identified (I^2^ > 50% detected using the Chi-square and I^2^ tests); otherwise, a fixed-effect model was used. Sensitivity analyses were conducted to investigate and resolve identified heterogeneity. We used the available data in the trials, and when both intention-to-treat (ITT) and per-protocol (PP) analyses were reported, we prioritized the ITT data. Median and interquartile range data were converted to means and standard deviations using the Meta-Analysis Accelerator calculator (24). Meta-regression analysis was performed when at least ten studies reported on a specific outcome and moderator (25) using OpenMeta (Analyst) software. An omnibus p-value of <0.05 indicated a statistically significant association (26). Subgroup analyses were carried out whenever feasible. Publication bias was evaluated for primary outcomes reported by ten or more studies using funnel plots, with symmetrical distribution indicating a lower risk of publication bias (27).

## 3 Results

### 3.1 Literature search

**Figure 1:**
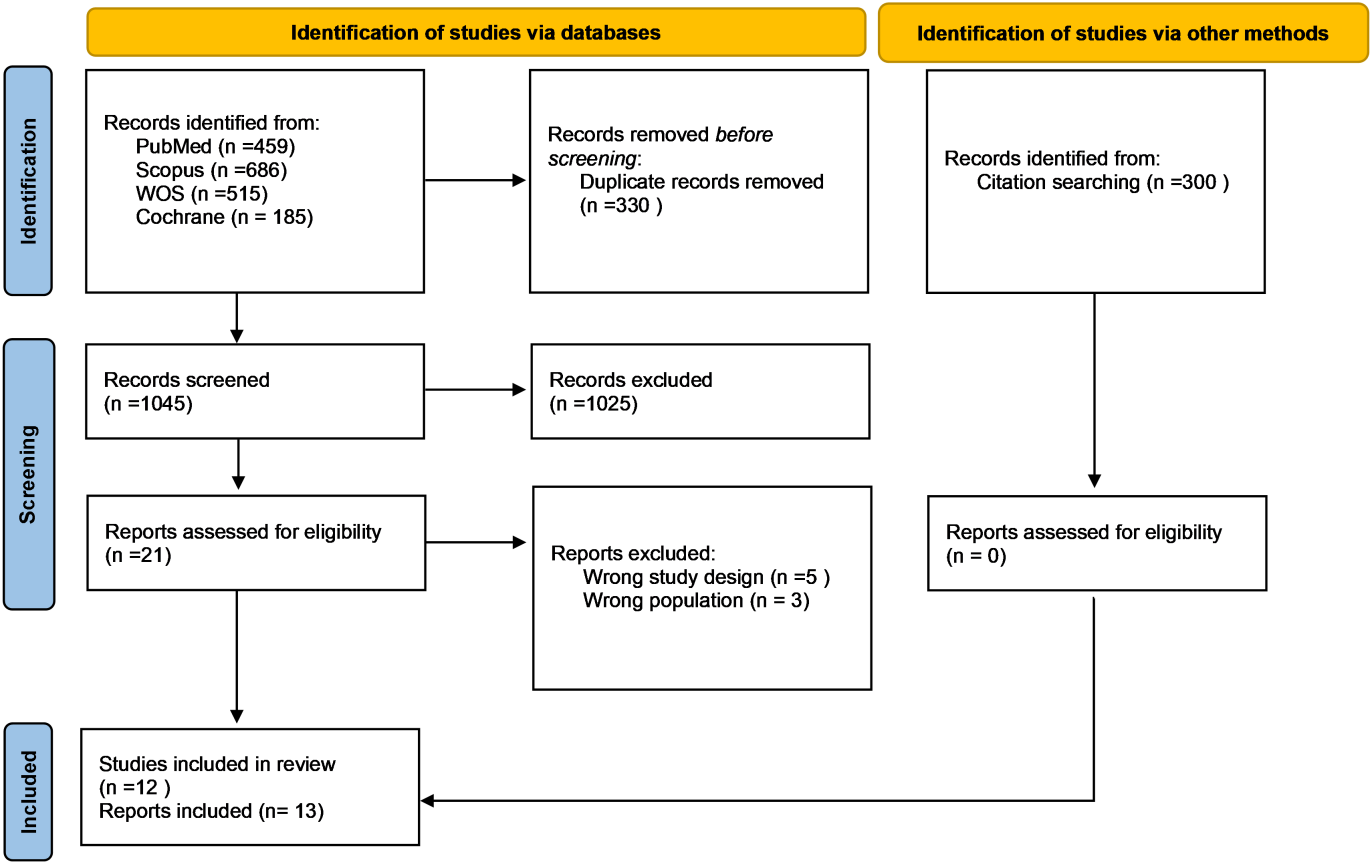
PRISMA flow diagram.

A systematic search was conducted across four databases (PubMed, Scopus, Web of Science, and Cochrane Library), yielding 1845 articles, of which 330 duplicates were excluded. After duplicate removal, 1045 articles underwent title and abstract screening. Of these, 21 studies qualified for full-text assessment, resulting in the inclusion of 12 clinical trials and one secondary analysis. The study selection process is detailed in the PRISMA flow diagram (**Figure 1**).

### 3.2 Characteristics of included studies

Of the 12 studies included in the review (14, 16, 28–37), only one was a parallel-arm randomized controlled trial (RCT) (Beck 2022) (37), while the remaining studies utilized a crossover design. Three trials were conducted in inpatient settings (Thabit 2022, Ware 2023, and Morrison 2021) (16, 29, 30). Two trials specifically involved children and adolescents (Dovc 2020, Ware 2023) (29, 35), whereas the others enrolled adult populations.

Regarding HCL systems, four trials employed the CamAPS FX system (Nwokolo 2023, Thabit 2022, Ware 2023, Boughton 2021) (15, 29–31), three utilized Medtronic 670G (Ozer 2020, Bode 2021, Hsu 2021) (14, 33, 36), and two evaluated Medtronic 780G (Morrison 2021, Dovc 2023) (16, 34). Two studies examined ultra-rapid insulin lispro (URLi) (Bode 2021, Nwokolo 2023) (31, 36), while the remaining studies assessed faster-acting insulin aspart (Fiasp). Follow-up durations across the studies ranged widely, from 24 hours to 17 weeks (**Tables 1**, **2**).

**Table 1:** Study characteristics.

Study ID	Registration Number	Study Design and Centers	Country	Study Setting	Total Participants	Population	Device Used	Insulin Used in Intervention Group	Control Details	Follow-up
Beck 2022 (37)	NCT 04200212	multicenter, RCT parallel arm	USA	Outpatient	275	Adults	iLet bionic pancreas	Fisap	Standard Insulin Aspart or Lispro using CL or conventional methods	13 weeks
Bode 2021 (36)	NCT 03760640	multicenter, RCT crossover	USA	Outpatient	42	Adults	Medtronic MiniMed 670G HCL system	URLi	Standard Insulin Lispro	4 weeks
Boughton 2021 (15)	NCT 04055480	multicenter, RCT crossover	UK, Austria, Switzerland	Outpatient	25	Adults	CamAPS FX Closed-Loop system	Fisap	Standard Insulin Aspart	8 weeks
Dovc 2020 (35)	NCT03212950	single-center, RCT, crossover	Slovenia	Inpatient	20	Adults	Fuzzy-logic control algorithm DreaMed GlucoSitter	Fisap	Standard Insulin Aspart	27 hours
Dovc 2023 (34)	NCT04853030	multicenter, RCT crossover	Slovenia, Austria	Outpatient and inpatient	30	Adolescents	Medtronic MiniMed 780G HCL system	Fisap	Standard Insulin Aspart	8 weeks
Hsu 2021 (33)	NCT 03554486	multicenter, RCT crossover	USA	Outpatient	19	Adults	Medtronic MiniMed 670G HCL system	Fisap	Standard Insulin Aspart	4 weeks
Lee 2021 (32)	ACTRN12619000469112	single-center, RCT, crossover	Australia	Outpatient	25	Adults	MiniMed 600	Fisap	Standard Insulin Aspart	17 weeks
Nwokolo 2023 (31)	NCT 05257460	multicenter, RCT crossover	United Kingdom	Outpatient	28	Adults	CamAPS FX Closed-Loop system	URLi	Standard Insulin Lispro	8 weeks
Thabit 2022 (30)	NCT 03579615	single-center, RCT, crossover	UK	Inpatient	16	Adults	CamAPS FX Closed-Loop system	Fisap	Standard Insulin Aspart	24 hours
Ware 2023 (29)	NCT 04759144	multicenter, RCT crossover	United Kingdom	Inpatient	25	Children	CamAPS FX Closed-Loop system	Fisap	Standard Insulin Aspart	8 weeks
Ozer 2020 (14)	NCT03977727	single-center, RCT, crossover	USA	Outpatient	40	Adults	Medtronic MiniMed 670G HCL system	Fisap	Standard Insulin Aspart	6 weeks
Morrison 2021 (16)	ACTRN12619000469112	single-center, RCT, crossover	Australia	Inpatient	16	Adults	Medtronic MiniMed 780G HCL system	Fisap	Standard Insulin Aspart	24 hours

RCT, randomized controlled trial; Fisap, faster insulin aspart; URLi, ultrarapid insulin lispro.

**Table 2:** Baseline study data.

Study ID	Participants	Age	BMI (Kg/M2)	Male n (%)	Duration of diabetes	Prior pump use duration	HbA1c, %	Insulin TDD
Beck 2022 (37)	221	43 (15.5)	28.75 (5.29)	107 (48.4)	24.97 (14)	NA	7.7 (1.2)	0.61 (0.21)
Bode 2021 (36)	42	47.8 (13.8)	27.2 (4.2)	15 (35.7)	29.6 (13.9)	1.4 (0.7)	7.07 (0.47)	0.53 (0.17)
Boughton 2021 (40)	25	38 (9)	26 (3.81)	12 (48)	22 (12)	NA	7.4 (0.8)	NA
Dovc 2020 (35)	20	21.3 (2.3)	22 (2)	9 (45)	13 (4.2)	10.8 (3.6)	7.5 (0.5)	NA
Dovc 2023 (34)	30	15 (1.7)	NA	14 (47)	7.8 (3.8)	NA	7.5 (0.9)	NA
Hsu 2021 (33)	19	40.4 (17.7)	NA	10 (53)	26.6 (12.3)	NA	7.17 (0.48)	0.77 (0.64)
Lee 2021 (32)	25	47.33 (15.72)	NA	13 (52)	25.67 (19.65)	8.67 (9.43)	6.93 (0.47)	0.53 (0.24)
Nwokolo 2023 (31)	28	44 (11)	29.6 (3.9)	18 (64)	29.6	NA	7.1 (0.9)	NA
Thabit 2022 (30)	16	32 (9)	25.7 (2.1)	7 (44)	17 (8)	NA	3.6(0.33)	NA
Ware 2023 (29)	25	5.2 (1.3)	NA	17 (68)	2.4 (1.2)	NA	7.2 (0.8)	0.74 (0.14)
Morrison 2021 (16)	16	47.33(16.26)	27.60 (3.74)	9(60)	29.33 (19.51)	10 (7.32)	7.0 (6.4, 7.2)	NA
Ozer 2021 (14)	37	45.7 (12.93)	27.1 (3.41)	67.6 (47.46)	NA	NA	7 (0.54)	NA

BMI, body mass index; HbA1c, glycated hemoglobin; TDD, total daily dose.

### 3.3 Risk of bias assessment

For crossover trials, we considered potential carryover effects. All included crossover studies implemented appropriate washout periods or randomized sequence allocation to minimize such bias. Risk of bias specific to crossover designs was evaluated using the ROB2 model tailored for crossover trials. Among the 11 crossover studies evaluated, they all demonstrated a low risk of bias across all domains, including randomization processes, carryover effects, deviations from intended interventions, missing outcome data, measuring of the outcomes, and selective result reporting, except for Ozer 2020. The study by Ozer et al. (14) raised concerns regarding the randomization method, as details about randomization procedures and allocation concealment were not reported. Additionally, Beck 2020 presented some concerns related to deviations from intended interventions due to an increased number of unscheduled visits in the intervention group during the COVID-19 pandemic (**Supplementary Figure 1**). A GRADE evidence profile demonstrates the certainty of evidence in **Table 3**.

**Table 3:** GRADE evidence profile.

Certainty assessment							Summary of findings				
Participants (studies) Follow-up	Risk of bias	Inconsistency	Indirectness	Imprecision	Publication bias	Overall certainty of evidence	Study event rates (%)		Relative effect (95% CI)	Anticipated absolute effects	
							With rapid acting lispro and aspart	With ultra-rapid acting lispro and aspart		Risk with rapid acting lispro and aspart	Risk difference with ultra-rapid acting lispro and aspart
TIR 70–180 mg/dL											
710 (11 RCTs)	not serious	not serious	not serious	serious^a^	none	⨁⨁⨁◯ Moderate^a^	352	358	–	–	MD 0.87% higher (0.21 lower to 1.95 higher)
Night-time TIR 70–180 mg/dL											
620 (9 RCTs)	not serious	not serious	not serious	not serious	none	⨁⨁⨁⨁ High	307	313	–	–	MD 1.9% lower (2.47 lower to 1.33 lower)
TBR <54 mg/dL											
632 (9 RCTs)	not serious	not serious	not serious	serious^a^	none	⨁⨁⨁◯ Moderate^a^	313	319	–	–	MD 0.05% lower (0.11 lower to 0.01 higher)
TBR <70 mg/dL											
657 (10 RCTs)	not serious	not serious	not serious	not serious	publication bias strongly suspected	⨁⨁⨁◯ Moderate	325	332	–	–	MD 0.34% lower (0.56 lower to 0.13 lower)
TAR >250 mg/dL											
487 (6 RCTs)	not serious	not serious	not serious	serious^a^	none	⨁⨁⨁◯ Moderate^a^	240	247	–	–	MD 0.36% lower (1.24 lower to 0.52 higher)
TAR >180 mg/dL											
670 (10 RCTs)	not serious	not serious	not serious	serious^a^	publication bias strongly suspected	⨁⨁◯◯ Low^a^	332	338	–	–	MD 0.34% lower (1.48 lower to 0.79 higher)
Glucose mean											
572 (10 RCTs)	not serious	not serious	not serious	serious^a^	publication bias strongly suspected	⨁⨁◯◯ Low^a^	256	316	–	–	MD 0.28 mg/dl lower (0.95 lower to 0.39 higher)
Glucose SD											
464 (6 RCTs)	not serious	not serious	not serious	serious^a^	none	⨁⨁⨁◯ Moderate^a^	229	235	–	–	MD 1.48 mg/dl lower (3.03 lower to 0.08 higher)
Glucose CV											
586 (9 RCTs)	not serious	not serious	not serious	not serious	none	⨁⨁⨁⨁ High	290	296	–	–	MD 0.78 lower (1.44 lower to 0.12 lower)
Insulin total daily dose											
403 (8 RCTs)	not serious	not serious	not serious	serious^a^	none	⨁⨁⨁◯ Moderate^a^	202	201	–	–	MD 0.52 Units higher (0.03 lower to 1.07 higher)
Total adverse events											
752 (11 RCTs)	not serious	not serious	not serious	not serious	none	⨁⨁⨁⨁ High	94/373 (25.2%)	133/379 (35.1%)	RR 1.38 (1.12 to 1.69)	94/373 (25.2%)	96 more per 1,000 (from 30 more to 174 more)
Infusion site reactions											
299 (5 RCTs)	not serious	not serious	not serious	not serious	strong association	⨁⨁⨁⨁ High	11/150 (7.3%)	32/149 (21.5%)	RR 2.77 (1.50 to 5.12)	11/150 (7.3%)	130 more per 1,000 (from 37 more to 302 more)
Hypoglycemic events											
784 (12 RCTs)	not serious	not serious	not serious	serious^a^	none	⨁⨁⨁◯ Moderate^a^	8/389 (2.1%)	3/395 (0.8%)	RR 0.35 (0.10 to 1.29)	8/389 (2.1%)	13 fewer per 1,000 (from 19 fewer to 6 more)

CI, confidence interval; MD, mean difference; RR, risk ratio.

Explanations

a. A wide confidence interval that does not exclude the appreciable harm or benefit.

### 3.4 Primary outcomes

#### 3.4.1 Time in range

**Figure 2:**
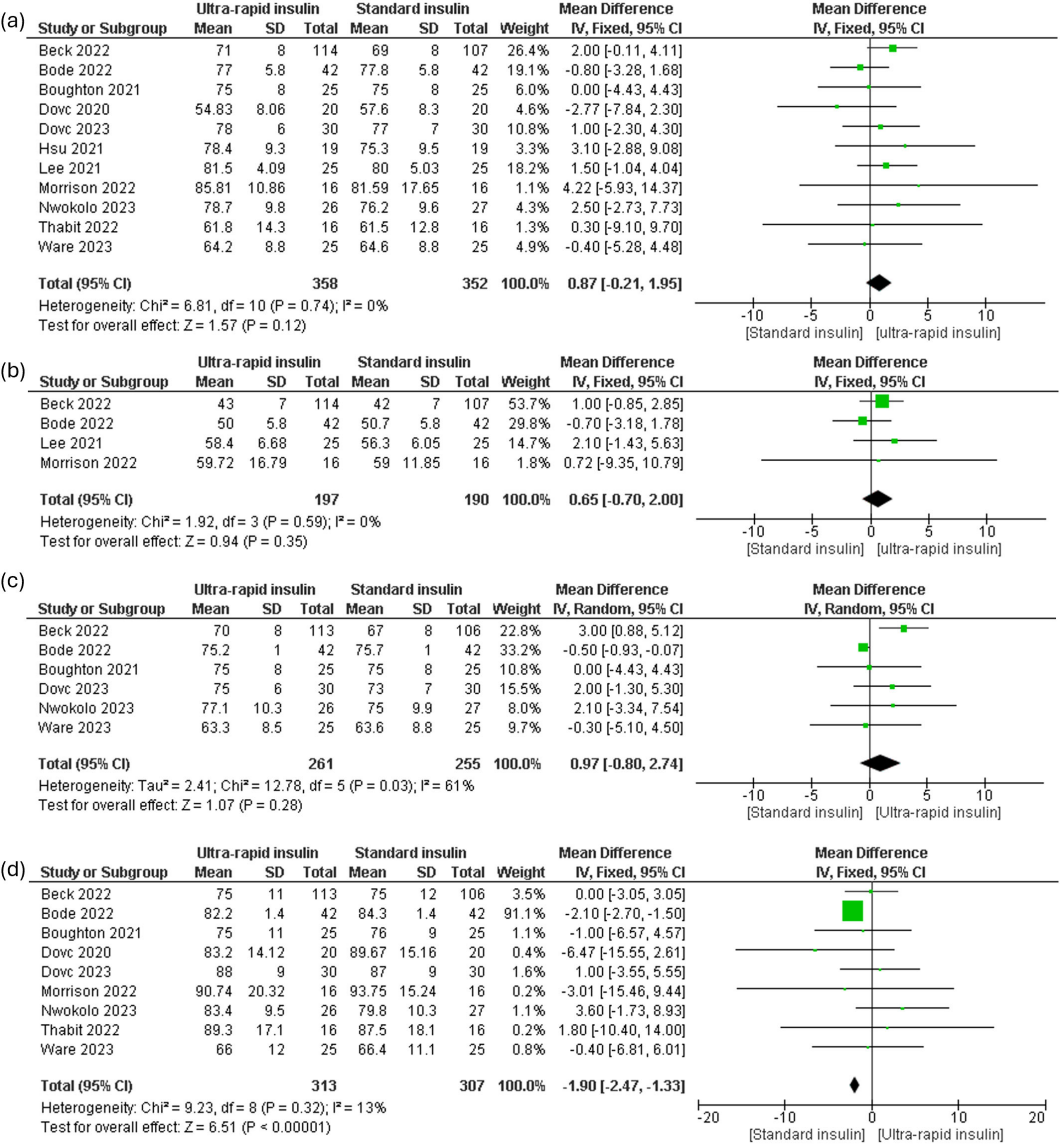
Forest plots of time in range (TIR): **(a)** All day TIR 70-180 mg/dl, **(b)** TIR 70-140 mg/dl, **(c)** Daytime TIR 70-180 mg/dl, **(d)** TIR 70-180 nighttime.

Pooled analysis of TIR within the 70–180 mg/dL range showed a slight, non-significant improvement in response to ultra-rapid insulin [mean difference (MD) 0.87%, 95% CI (-0.21 to 1.85), P = 0.12, I² = 0%] compared to standard insulin (**Figure 2a**). Analysis of TIR within the tighter range of 70–140 mg/dL also indicated no significant improvement [MD 0.65%, 95% CI (-0.7 to 2.0), P = 0.35, I² = 0%] (**Figure 2b**). Similarly, subgroup analyses for daytime and nighttime TIR (70–180 mg/dL) revealed statistically non-significant changes in ultra-rapid insulin users during daytime [MD 0.97%, 95% CI (-0.8 to 2.74), P = 0.28, I² = 61%] but a significant difference during nighttime [MD -1.9%, 95% CI (-2.47 to -1.33), P = 0.0001, I² = 13%] (**Figures 2c, d**). However, removing the Bode 2022 study resolved heterogeneity and indicated a significant improvement for daytime TIR (70–180 mg/dL) with ultra-rapid insulin [MD 2.05%, 95% CI (0.55 to 3.56), P = 0.007, I² = 0%] (**Supplementary Figure 2**).

Meta-regression analysis assessing the influence of diabetes duration and HbA1c levels on TIR (70–180 mg/dL) revealed no statistically significant associations (p > 0.05) (**Supplementary Table 2**, **Supplementary Figures 3**, **4**). Assessment of publication bias demonstrated a symmetrical distribution of the effect size, suggesting minimal risk of publication bias (**Supplementary Figure 5**).

### 3.5 Secondary outcomes:

#### 3.5.1 Time below range

**Figure 3:**
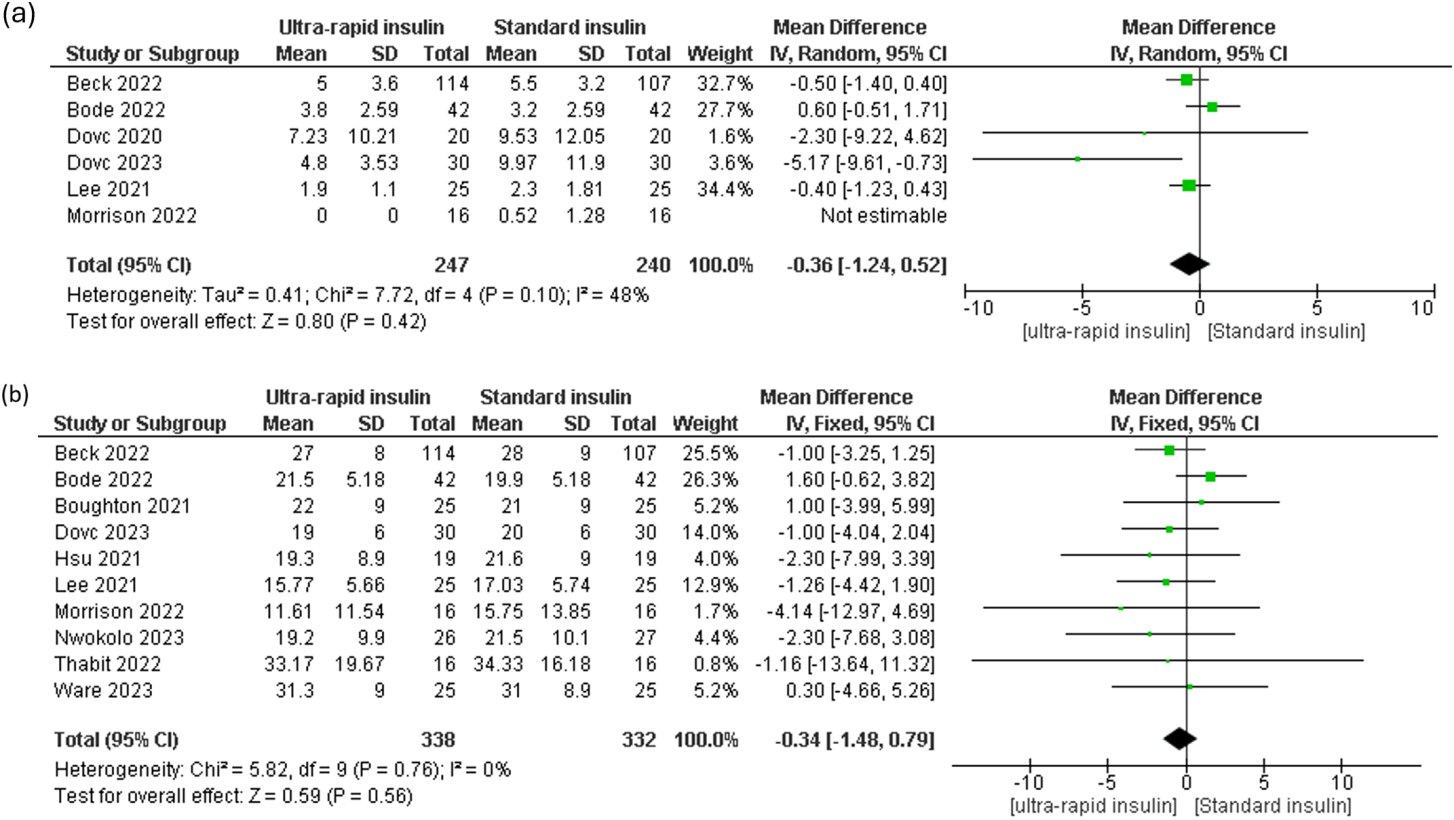
Forest plots of Time below range (TBR): **(a)** All day TBR 54 mg/dl, **(b)** All day TBR 70 mg/dl.

Analysis of TBR <54 mg/dL showed a slight but non-significant reduction with ultra-rapid insulin [MD -0.05%, 95% CI (-0.11 to 0.01), P = 0.11, I² = 0%] (**Figure 3a**). However, daytime and nighttime TBR <54 mg/dL percentages were significantly reduced in ultra-rapid insulin users [daytime: MD -0.1%, 95% CI (-0.13 to -0.06), P = 0.0001, I² = 0%; nighttime: MD 0.09%, 95% CI (0.02 to 0.16), P = 0.01, I² = 0%] (**Supplementary Figures 6a, b**). For TBR <70 mg/dL, the meta-analysis demonstrated a significant overall reduction with ultra-rapid insulin [MD -0.34%, 95% CI (-0.56 to -0.13), P = 0.002, I² = 0%] (**Figure 3b**). Daytime TBR <70 mg/dL showed a significant improvement with ultra-rapid insulin [MD -0.53%, 95% CI (-0.85 to -0.2), P = 0.002, I² = 50%] (**Supplementary Figure 7a**), whereas nighttime data revealed no significant difference [MD 0.13%, 95% CI (-0.43 to 0.69), P = 0.65, I² = 86%] (**Supplementary Figure 7b**).

Sensitivity analysis indicated that excluding the Bode 2022 study eliminated heterogeneity in daytime TBR <70 mg/dL, resulting in continued significance favoring ultra-rapid insulin [MD -0.31%, 95% CI (-0.61 to -0.01), P = 0.04, I² = 0%] (**Supplementary Figure 8**). Similarly, removing Boughton 2021 from nighttime TBR <70 mg/dL resolved heterogeneity and shifted results significantly toward ultra-rapid insulin [MD -0.3%, 95% CI (-0.45 to -0.16), P = 0.0001, I² = 0%] (**Supplementary Figure 9**).

Meta-regression analyses assessing the influence of diabetes duration and HbA1c levels on TBR <70 mg/dL revealed no significant associations (p > 0.05) (**Supplementary Table 1**, **Supplementary Figures 10**, **11**). Assessment for publication bias showed an asymmetrical distribution, indicating possible publication bias (**Supplementary Figure 12**).

#### 3.5.2 Time above range

**Figure 4:**
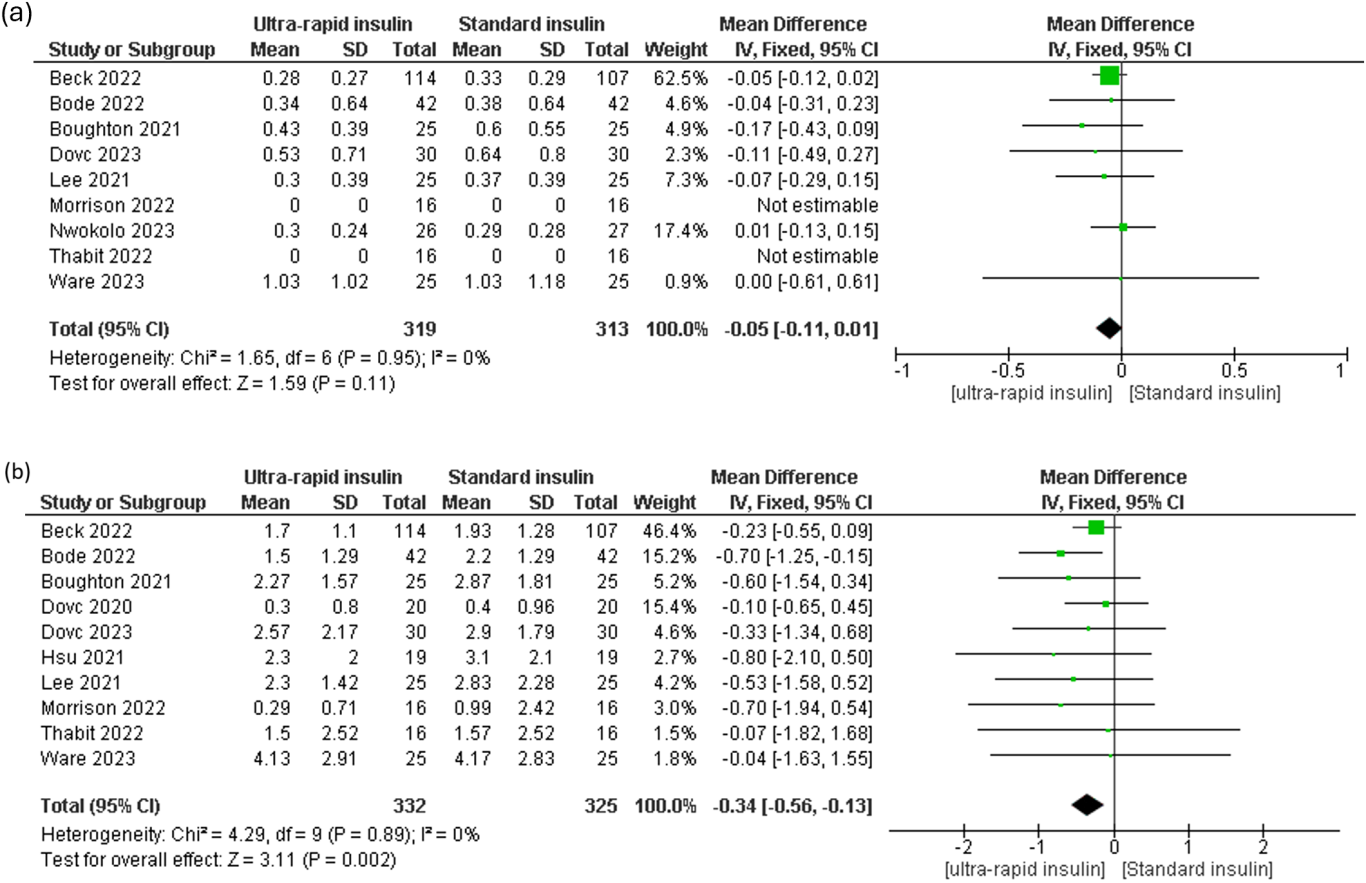
Forest plots of Time above range (TAR): **(a)** All day TAR 250 mg/dl, **(b)** All day TAR 180 mg/dl.

Analysis of TAR >250 mg/dL and TAR >180 mg/dL demonstrated non-significant overall reductions with ultra-rapid insulin [TAR >250 mg/dL: MD -0.36%, 95% CI (-1.24 to 0.52), P = 0.42, I² = 48%; TAR >180 mg/dL: MD -0.34%, 95% CI (-1.48 to 0.79), P = 0.56, I² = 0%] (**Figures 4a, b**). Daytime TAR >250 mg/dL also showed no significant difference [MD -1.42%, 95% CI (-3.9 to 1.07), P = 0.26, I² = 96%] (**Supplementary Figure 13a**), while nighttime TAR >250 mg/dL was significantly higher with ultra-rapid insulin [MD 0.35%, 95% CI (0.11 to 0.5), P = 0.005, I² = 0%] (**Supplementary Figure 13b**). Analysis of daytime TAR >180 mg/dL revealed no significant difference [MD -0.29%, 95% CI (-2.06 to 1.48), P = 0.75, I² = 58%] (**Supplementary Figure 14a**), whereas nighttime TAR >180 mg/dL significantly increased in ultra-rapid insulin users compared to standard insulin [MD 2.19%, 95% CI (1.66 to 2.72), P = 0.0001, I² = 30%] (**Supplementary Figure 14b**).

Sensitivity analysis indicated that removing the Dovc 2023 study resolved heterogeneity for TAR >250 mg/dL [MD -0.22%, 95% CI (0.75 to 0.32), P = 0.43, I² = 0%] (**Supplementary Figure 15**). Exclusion of Bode 2022 resolved heterogeneity resulting in lower daytime TAR >180 mg/dL in ultra-rapid insulin users [MD -1.4%, 95% CI (-2.96 to 0.16), P = 0.08, I² = 0%] (**Supplementary Figure 16**).

Meta-regression analysis found no significant relationship between TAR >180 mg/dL and diabetes duration or HbA1c levels (**Supplementary Table 2**, **Supplementary Figures 17**, **18**). Publication bias evaluation for TAR >180 mg/dL using funnel blot showed an asymmetrical distribution of effect size, suggesting the presence of publication bias (**Supplementary Figure 19**).

#### 3.5.3 Glycemic variability

**Figure 5:**
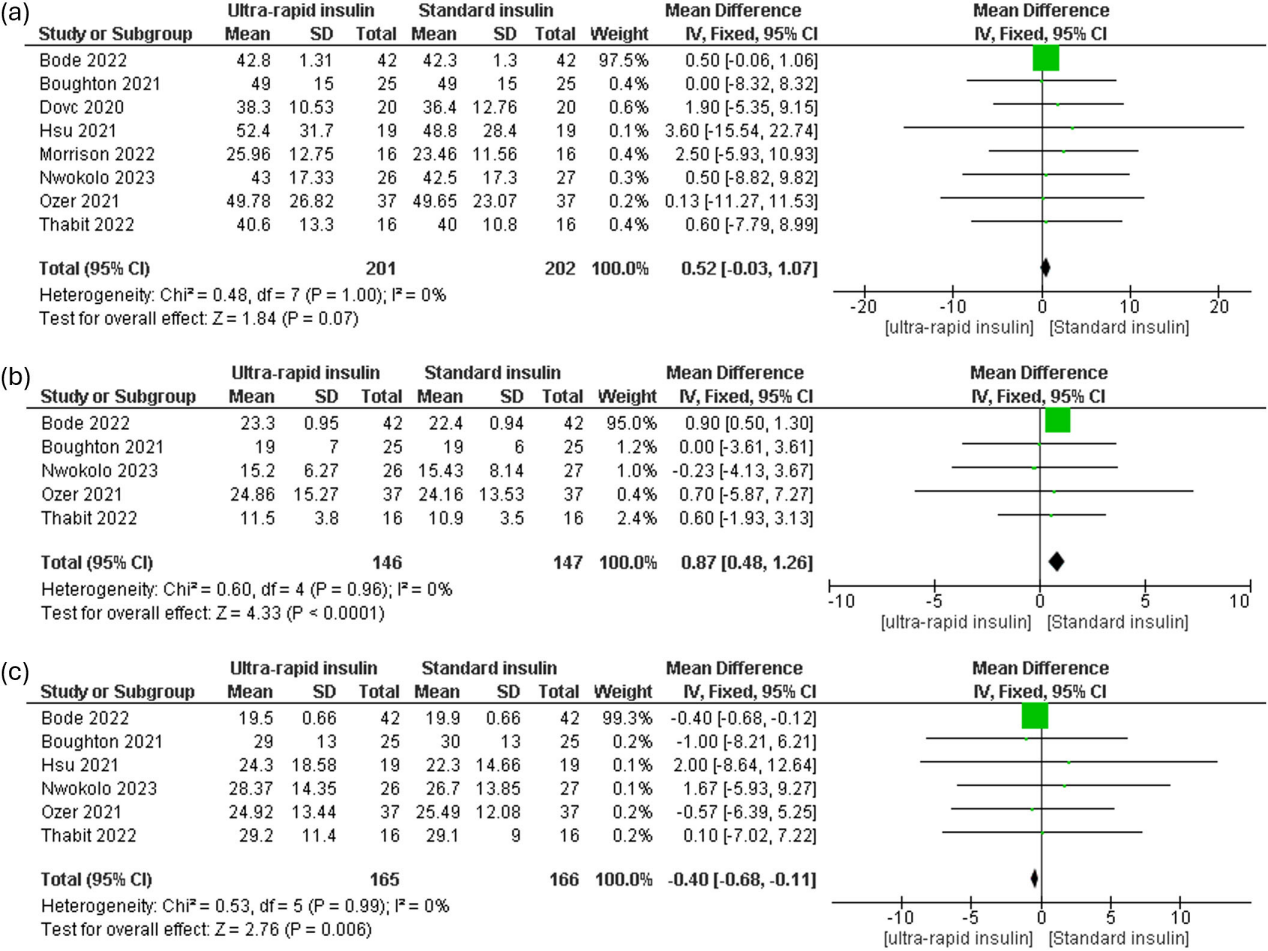
Forest plots of glycemic variability: **(a)** All day mean glucose, **(b)** Standard deviation of glucose, **(c)** All day glucose coefficient variation (CV).

Ultra-rapid insulin showed non-significant reductions in mean glucose levels compared to standard insulin across all-day [MD -0.28 mg/dL, 95% CI (-0.95 to 0.39), P = 0.42, I² = 0%], daytime [MD -1.42 mg/dL, 95% CI (-3.97 to 1.13), P = 0.27, I² = 0%], and nighttime periods [MD 23.59 mg/dL, 95% CI (-4.19 to 51.36), P = 0.10, I² = 99%] (**Figure 5a**, **Supplementary Figures 20a, b**). Similarly, glucose standard deviation (SD) showed a non-significant downward trend [MD -1.48, 95% CI (-3.03 to 0.08), P = 0.06, I² = 0%] (**Figure 5b**). However, the coefficient of variation (CV) demonstrated a significant improvement with ultra-rapid insulin for both all-day [MD -0.78, 95% CI (-1.44 to -0.12), P = 0.02, I² = 0%] and daytime periods [MD -1.08, 95% CI (-2.07 to -0.08), P = 0.03, I² = 24%] (**Figures 5c**, **Supplementary Figures 21a**), though nighttime CV showed no significant differences [MD -0.71, 95% CI (-1.78 to 0.35), P = 0.19, I² = 0%] (**Supplementary Figure 21b**).

Heterogeneity in nighttime mean glucose levels was resolved upon excluding the Ware 2023 study [MD 0.1 mg/dL, 95% CI (-1.03 to 1.24), P = 0.86, I² = 0%] (**Supplementary Figure 22**). Removing Beck 2022 from daytime CV resolved the heterogeneity and changed results to non-significance [MD -0.4 mg/dl, 95% CI [-1.57 to 0.77], *P =* 0.5, I^2^ = 0%] (**Supplementary Figure 23**). Meta-regression analyses showed no significant relationships between mean glucose levels and either diabetes duration or HbA1c levels (**Supplementary Table 2**, **Supplementary Figures 24**, **25**). Funnel plot assessment for publication bias in mean glucose outcomes revealed an asymmetrical distribution of effect size, indicating possible publication bias (**Supplementary Figure 26**).

#### 3.5.4 Insulin total daily insulin dose

**Figure 6:**
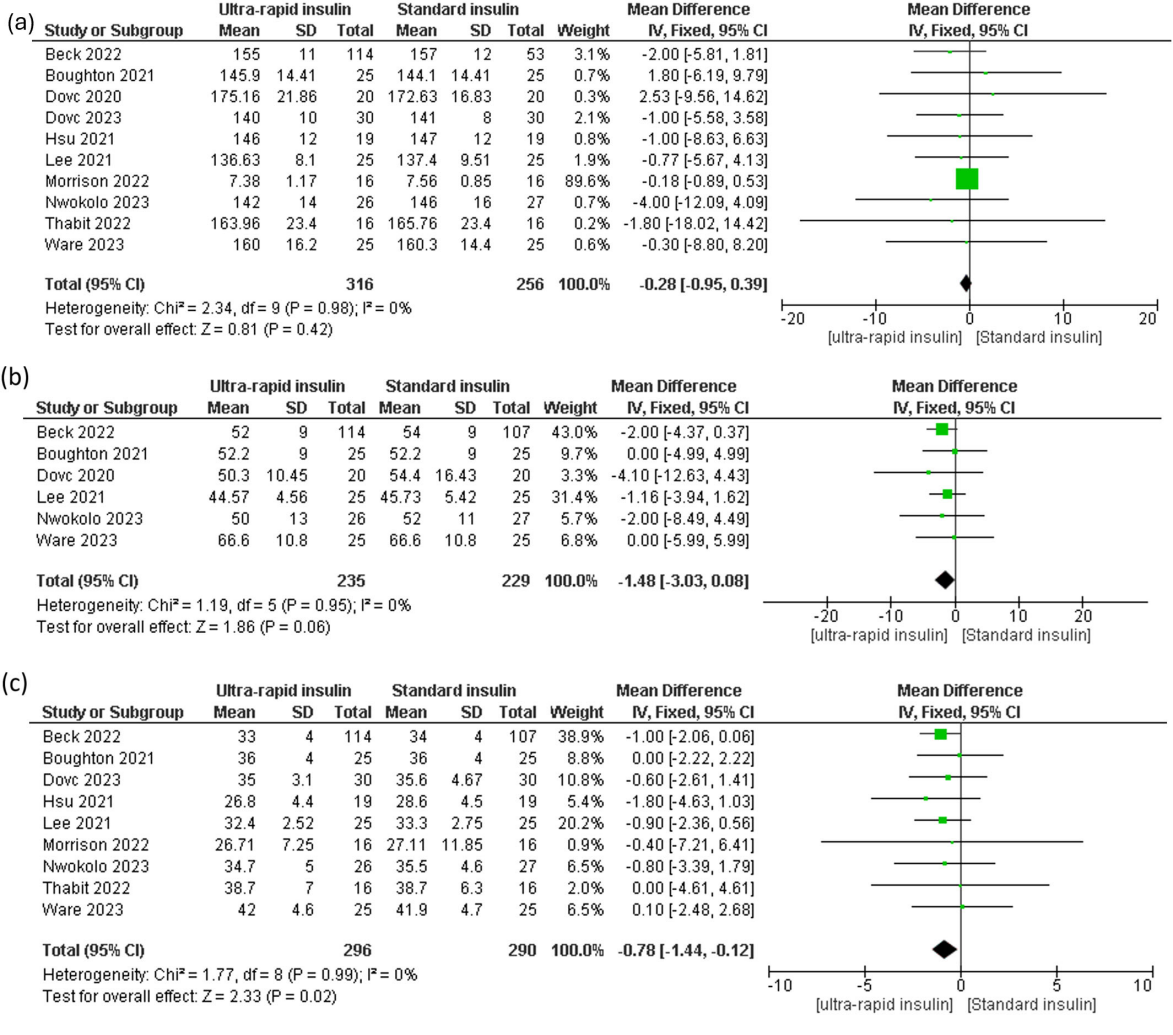
Forest plots of Insulin dose: **(a)** Total daily insulin dose (TDD), **(b)** Bolus insulin dose, **(c)** Basal insulin dose.

Ultra-rapid insulin demonstrated a trend toward reducing total daily insulin doses, approaching statistical significance [MD 0.52 Units, 95% CI (-0.03 to 1.07), P = 0.07, I² = 0%] (**Figure 6a**). Bolus insulin doses were significantly lower in ultra-rapid insulin users [MD 0.87 Units, 95% CI (0.48 to 1.26), P = 0.0001, I² = 0%] (**Figure 6b**). Additionally, basal insulin delivery was significantly reduced among ultra-rapid insulin users [MD -0.4 Units, 95% CI (-0.68 to -0.11), P = 0.006, I² = 0%] (**Figure 6c**).

#### 3.5.5 Adverse events

Regarding safety outcomes, the overall frequency of adverse events was significantly higher with ultra-rapid insulin [Risk Ratio (RR) 1.38, 95% CI (1.21 to 1.69), P = 0.002, I² = 21%] (**Supplementary Figure 27a**). Infusion site reactions also occurred more frequently in the ultra-rapid insulin group [RR 2.77, 95% CI (1.5 to 5.12), P = 0.001, I² = 29%] (**Supplementary Figure 27b**). Conversely, no significant differences were observed between ultra-rapid and standard insulin groups regarding hypoglycemic events [RR 0.35, 95% CI (0.10 to 1.29), P = 0.12], diabetic ketoacidosis (DKA) events [RR 4.70, 95% CI (0.23 to 96.7), P = 0.32], or device-related events [RR 1.24, 95% CI (0.87 to 1.77), P = 0.23, I² = 19%] (**Supplementary Figures 27c–e**).

#### 3.5.6 Post-meal glycemic control

During the two-hour post-lunch period, ultra-rapid insulin did not significantly reduce blood glucose incremental area under the curve (IAUC) [MD -30.1, 95% CI (-68.45 to 8.24), P = 0.12, I² = 0%] (**Supplementary Figure 28a**). However, ultra-rapid insulin significantly lowered mean glucose levels within two hours following breakfast [MD -57.57 mg/dL, 95% CI (-108.74 to -6.39), P = 0.03, I² = 65%], but not after dinner [MD -33.06 mg/dL, 95% CI (-74.43 to 8.32), P = 0.12, I² = 4%] (**Supplementary Figures 28b, c**). Furthermore, the four-hour blood glucose area under the curve (AUC) following lunch did not significantly differ between ultra-rapid insulin and standard insulin [MD -42.97, 95% CI (-128.27 to 42.33), P = 0.32, I² = 9%] (**Supplementary Figure 29a**). Similar non-significant findings were observed for breakfast [MD -9.27, 95% CI (-84.05 to 65.5), P = 0.81, I² = 0%] and dinner [MD -42.97, 95% CI (-128.27 to 42.33), P = 0.32, I² = 9%] (**Supplementary Figures 29b, c**).

Regarding post-meal TIR (70–180 mg/dL), ultra-rapid acting insulin showed no significant improvement compared to standard insulin after lunch [MD 0.9%, 95% CI (-3.0 to 4.8), P = 0.65, I² = 0%], breakfast [MD 3.94%, 95% CI (-0.7 to 8.58), P = 0.10, I² = 34%], or dinner [MD 1.42%, 95% CI (-2.78 to 5.62), P = 0.51, I² = 71%] (**Supplementary Figures 30a–c**). Similarly, ultra-rapid insulin did not significantly reduce post-meal TBR <70 mg/dL at lunch [MD 0.55%, 95% CI (-0.47 to 1.56), P = 0.29, I² = 0%], breakfast [MD 0.35%, 95% CI (-0.51 to 1.2), P = 0.43, I² = 52%], or dinner [MD 0.34%, 95% CI (-0.71 to 1.0), P = 0.52, I² = 7%] (**Supplementary Figures 31a–c**). Additionally, ultra-rapid insulin showed non-significant reductions in TAR >180 mg/dL following lunch [MD -1.35%, 95% CI (-5.63 to 2.94), P = 0.54, I² = 0%], breakfast [MD -3.47%, 95% CI (-11.39 to 4.44), P = 0.39, I² = 62%], and dinner [MD -1.41%, 95% CI (-9.54 to 6.73), P = 0.73, I² = 67%] (**Supplementary Figures 32a–c**).

#### 3.5.7 Post-exercise glycemic control

Ultra-rapid acting insulin showed no significant improvement in TIR (70–180 mg/dL) within two hours post-exercise [MD 3.77%, 95% CI [-9.37 to 1.84], *P =* 0.19, I^2^ = 99%] (**Supplementary Figure 33a**). Similarly, there were no significant differences between ultra-rapid and standard insulin regarding mean glucose levels [MD 0.34 mg/dl, 95% CI [-0.71 to 1.39], *P =* 0.52, I^2^ = 0%] (**Supplementary Figure 33b**), TBR (<70 mg/dl) [MD 0.4%, 95% CI [-0.64 to 1.44], *P =* 0.5, I^2^ = 0%] (**Supplementary Figure 33c**), TAR (>180 mg/dl) [MD 0%, 95% CI [-8.95 to 8.95], *P =* 1%] (**Supplementary Figure 33d**), or TAR (>250 mg/dl) [MD -2%, 95% CI [-4.33 to 0.33], *P =* 0.09] (**Supplementary Figure 33e**). Sensitivity analysis removing Morrison 2022 resolved heterogeneity for TIR (70–180 mg/dL) post-exercise, resulting in outcomes that no longer favored ultra-rapid insulin [MD -2.37%, 95% CI [-9.86 to 5.12], *P =* 0.54. I^2^ = 0] (**Supplementary Figure 34**).

#### 3.5.8 Psychological outcomes

Ultra-rapid-acting insulin did not significantly influence psychological outcomes as measured by the Hypoglycemia Fear Scale [MD 0.11, 95% CI [-3.68 to 3.89], *P =* 0.96, I^2^ = 0%] (**Supplementary Figure 35a**). On the other hand, Insulin Delivery Systems: Perspectives, Ideas, Reflections, and Expectations [INSPIRE score] showed a near-significant increase among ultra-rapid insulin users [MD 3.98, 95% CI [-0.24 to 8.2], P *=* 0.06, I^2^ = 20] (**Supplementary Figure 35b**).

### 3.6 Subgroup analysis

We conducted subgroup analyses based on study location, population subgroups, device type, insulin type, and study duration. Subgroup analysis for closed-loop devices showed no significant changes in TIR (70-180 mg/dl) among patients using the CamAPX FX [MD 0.56%, 95% CI (-2.11, 3.22), P=0.68, I²=0%], Medtronic 670G [MD -0.23%, 95% CI (-2.52, 2.06), P=0.85, I²=28%], or Medtronic 870G [MD 1.31%, 95% CI (-1.83, 4.45), P=0.41, I²=0%] devices (**Supplementary Figure 36**). In insulin subgroup analyses, Fiasp showed a borderline significant increase in TIR (70-180 mg/dl) [MD 1.20%, 95% CI (-0.04, 2.43), P=0.06, I²=0%], while URLi demonstrated no significant effect [MD -0.19%, 95% CI (-2.43, 2.05), P=0.87, I²=20%]; the difference between these two insulins was also not significant (**Supplementary Figure 37**). Additional details can be found in **Supplementary Table 3**.

## 4 Discussion

Our systematic review and meta-analysis comprehensively assessed the impact of integrating ultra-rapid-acting insulin (URAI) with hybrid closed-loop (HCL) systems, providing a direct comparison to standard insulin across key glycemic metrics and safety outcomes, including TIR, TBR, and TAR metrics, mean glucose levels, glucose variability, insulin dosage, adverse events, hypoglycemia, and DKA. Although our findings indicated no significant differences between URAI and standard insulin in terms of TIR (both 70-180 mg/dL and 70-140 mg/dL) or TAR (both >180 mg/dL and >250 mg/dL), URAI demonstrated a clinically meaningful advantage by significantly reducing the time spent in mild hypoglycemia (<70 mg/dL), with a mean difference of 0.33%. Moreover, URAI significantly lowered glucose variability (coefficient of variation) by an average of 0.79%, suggesting more stable glycemic control. Conversely, the mean glucose level and total daily insulin dose did not differ significantly between the groups. Importantly, adverse events, particularly infusion site reactions, occurred significantly more frequently with URAI, underscoring the necessity for clinicians to balance the glycemic benefits of URAI against the increased risk of local adverse reactions. However, reassuringly, rates of severe hypoglycemia, DKA, and related serious events were not increased with URAI. These insights highlight the potential clinical utility of URAI in enhancing glycemic stability while emphasizing careful monitoring of infusion-site tolerability during clinical application.

A previous meta-analysis by Stamati et al. (11) evaluating URAI, specifically Fiasp, and URLi, in patients with T1DM utilizing continuous subcutaneous insulin infusion (CSII) systems reported the superiority of URAI analogs over rapid-acting insulin analogs (RAIAs) in increasing time spent within normoglycemia (70–180 mg/dL). This finding contrasts with the results of our current analysis. However, consistent with our findings, their analysis demonstrated a significant reduction in time spent in hypoglycemia (<70 mg/dL) alongside an increased incidence of infusion site reactions in the URAI group. Notably, Stamati’s meta-analysis did not clearly define statistical significance nor explicitly include hybrid closed-loop (HCL) systems, limiting direct comparability with our study.

Additionally, two simultaneous but separate meta-analyses individually assessing Fiasp and URLi against rapid insulin analogs or placebo reported no discernible differences in TIR or mean glucose levels. Interestingly, URLi was associated with fewer hypoglycemic events but a higher frequency of infusion site reactions, whereas Fiasp showed no notable differences in adverse events compared to rapid aspart insulin (38, 39). Our results differ from these later studies primarily due to methodological variations, including individual analyses of each URAI and the inclusion of placebo-controlled trials in these two meta-analyses. Furthermore, neither study specifically evaluated URAI performance within HCL systems, emphasizing the uniqueness and clinical relevance of our analysis to real-world applications and device-specific glycemic outcomes.

Given their unique pharmacokinetic profiles, the advent of faster insulin analogs (URAI) was anticipated to substantially enhance closed-loop system performance by accelerating insulin action onset and offset following delivery, thus enabling better glycemic control (40). Contrary to these expectations, our meta-analysis indicated that URAI’s primary advantages were limited to significant reductions in time spent below the glycemic target range and a notable improvement in glucose variability, as reflected by the reduced coefficient of variation. However, these benefits were offset by an increased frequency of adverse events, particularly infusion site reactions. Notably, our findings challenge prior predictions (11), demonstrating that URAI exacerbated, rather than reduced, infusion site complications in clinical practice. To address this limitation, ongoing research is exploring innovative strategies such as improving insulin delivery techniques through the implementation of infusion site modifications, including adjusting the insertion angle and the infusion protocols in AID systems and integrating novel technologies, including insulin infusion site warming devices, to enhance insulin absorption efficiency and minimize adverse reactions (41–43). These ongoing investigations underscore the critical need for continuous optimization of insulin delivery methods to fully harness the potential benefits of URAI within closed-loop systems.

To enhance clinical applicability and interpretation, our meta-analysis incorporated focused subgroup analyses of two prominent HCL systems extensively studied in individual trials: the Cam-APS FX Closed-Loop system and the Medtronic MiniMed 670G HCL system. These devices are among the earliest commercially approved and widely adopted technologies in diabetes management (44). Thus, their detailed performance evidence using URAI is required. Interestingly, despite the Cam-APS FX being the only licensed system to utilize ultra-rapid-acting insulin (URAI) analogs (45), our analysis uncovered a lack of substantial glycemic improvement coupled with a higher frequency of adverse events when using URAI analogs with this system. Conversely, the Medtronic MiniMed 670G demonstrated superior performance by achieving meaningful improvements in glycemic outcomes by reducing time spent in hypoglycemia (<70 mg/dl) and glucose variability without increasing adverse events such as hypoglycemia, DKA, or infusion site reactions. This finding is clinically significant, highlighting potential device-specific interactions with URAI that could influence both efficacy and safety profiles. These observations underscore the importance of device selection in clinical practice and call for further targeted research into optimizing URAI utilization within specific HCL systems to maximize patient outcomes and minimize potential risks.

We recognize several limitations in our current study that warrant consideration when interpreting the findings. Primarily, our conclusions rely heavily on the available primary evidence, which, despite an extensive and systematic literature review, is limited to a small number of RCTs with short duration and relatively small sample sizes, with only two RCTs having data for URLi. Most included studies used a crossover design, which may introduce carryover, period, or learning effects. While most trials implemented washout periods and proper randomization to minimize these biases, this design may limit generalizability and requires cautious interpretation. The included studies varied significantly in terms of quality and consistency, potentially affecting the robustness of our outcomes. Specifically, while we evaluated infusion site reactions as a safety outcome, the underlying mechanisms driving these adverse events remain unclear due to a lack of detailed reporting in the primary studies. Additionally, our analysis faced constraints in terms of generalizability, given the scarcity of data addressing the effects of URAI in patients with type 2 diabetes mellitus (T2DM) and pediatric populations; notably, we identified only two studies involving children and none involving patients with non-insulin-dependent diabetes. Thus, future research should prioritize the generation of robust real-world evidence from broader patient cohorts, including individuals with T2DM and pediatric groups. Detailed investigations into the long-term outcomes and patient-specific factors affecting efficacy and safety are crucial. Such studies will provide invaluable insights, enabling clinicians to make more informed decisions and ultimately optimizing the clinical application of URAI in hybrid closed-loop systems and enhancing patients’ treatment satisfaction and quality of life.

## 5 Conclusion

Our systematic review and meta-analysis demonstrated that the use of URAIs instead of standard insulin within HCL systems provides clinically meaningful improvements in glycemic control, particularly by significantly reducing nighttime hyperglycemia, both at moderate (TAR >180 mg/dL) and severe (TAR >250 mg/dL) levels. These findings have important clinical implications, suggesting that URAI may be particularly beneficial in optimizing overnight glycemic control, a period often challenging for patients with diabetes. Depending on our comparison between URAIs and standard insulin, individualized treatment selection is crucial; Despite offering improved glycemic stability and reduced hypoglycemia, the cost appears to be an increase in infusion site reactions, necessitating careful risk-benefit assessment. To further validate and expand upon these promising results, future research should prioritize conducting robust trials with larger patient cohorts, extended follow-up periods, and targeted inclusion of populations currently underrepresented in existing studies, notably individuals with type 2 diabetes mellitus and pediatric patients.

## Data Availability

The original contributions presented in the study are included in the article/**Supplementary Material**. Further inquiries can be directed to the corresponding author.
