# Supplementary material for: Ultra-rapid lispro or fast-acting aspart compared to standard insulin lispro and aspart using closed-loop insulin therapy: a systematic review and meta-analysis of randomized control trials
Source: Front Endocrinol (Lausanne). 2025 Jun 6;16:1600157. doi: 10.3389/fendo.2025.1600157 (PMC12178897; doi:10.3389/fendo.2025.1600157)
Supplement: Supplementary file 1 [file DataSheet1.pdf]

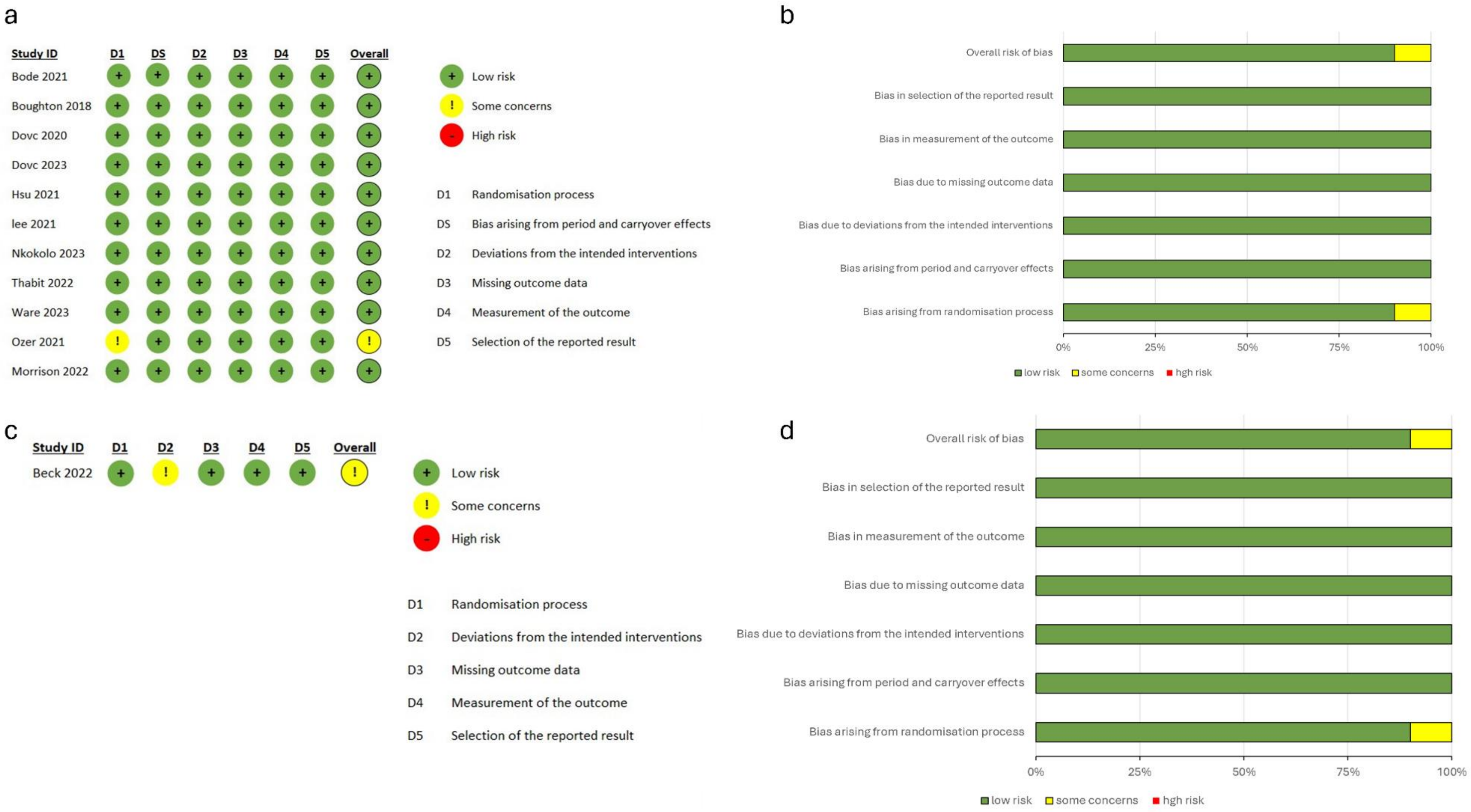

Supplementary Figure 1. Risk of bias, (a) traffic light table for crossover randomized controlled trials, (b) stacked bar graph for crossover randomized controlled trials, (c) traffic light table for parallel randomized controlled trials, (d) stacked bar graph for crossover randomized controlled trials

	Ultra-rapid insulin			Standard insulin				Mean Difference
**Study or Subgroup**	**Mean**	**SD**	**Total**	**Mean**	**SD**	**Total**	**Weight**	**IV, Random, 95% CI**
Beck 2022	70	8	113	67	8	106	50.3%	3.00 [0.88, 5.12]
Bode 2022	75.2	1	42	75.7	1	42	0.0%	-0.50 [-0.93, -0.07]
Boughton 2021	75	8	25	75	8	25	11.5%	0.00 [-4.43, 4.43]
Dovc 2023	75	6	30	73	7	30	20.8%	2.00 [-1.30, 5.30]
Nwokolo 2023	77.1	10.3	26	75	9.9	27	7.6%	2.10 [-3.34, 7.54]
Ware 2023	63.3	8.5	25	63.6	8.8	25	9.8%	-0.30 [-5.10, 4.50]
**Total (95% CI)**			**219**			**213**	**100.0%**	**2.05 [0.55, 3.56]**

Heterogeneity: Tau² = 0.00; Chi² = 2.52, df = 4 (P = 0.64); I² = 0%

Test for overall effect: Z = 2.68 (P = 0.007)

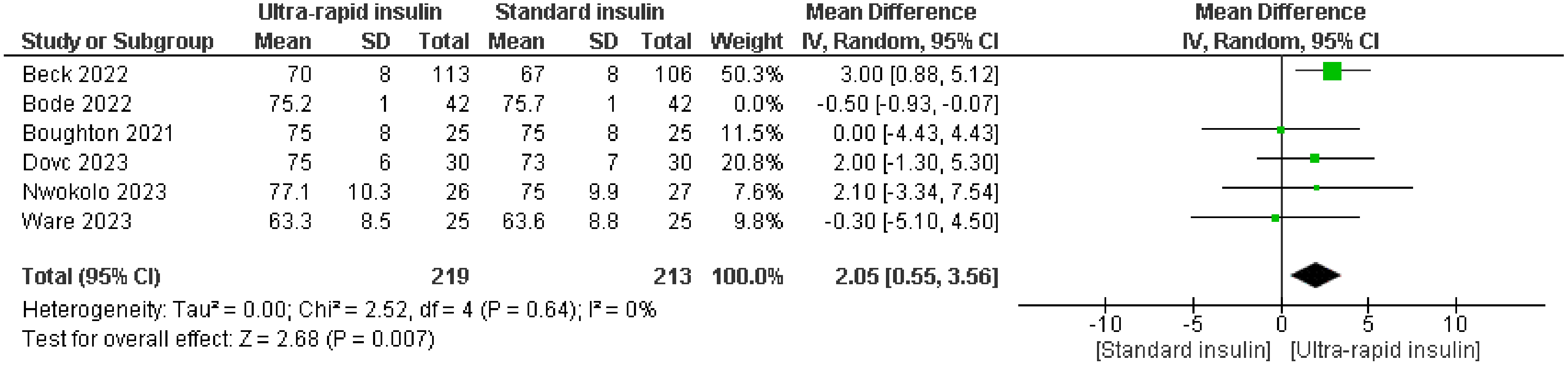

Supplementary Figure 2. TIR 70-180 mg/dl daytime sensitivity analysis

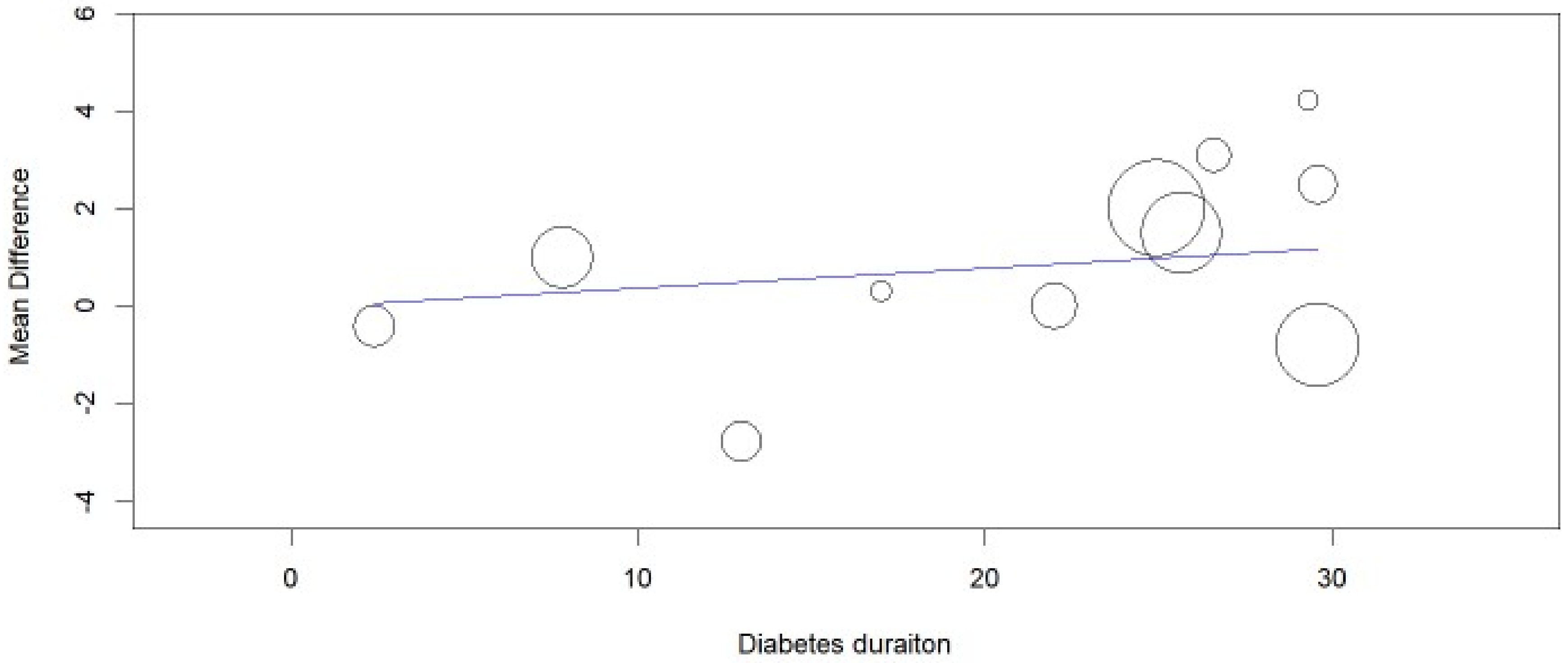

Supplementary Figure 3. TIR 70-180 mg/dl meta-regression analysis according to diabetes duration

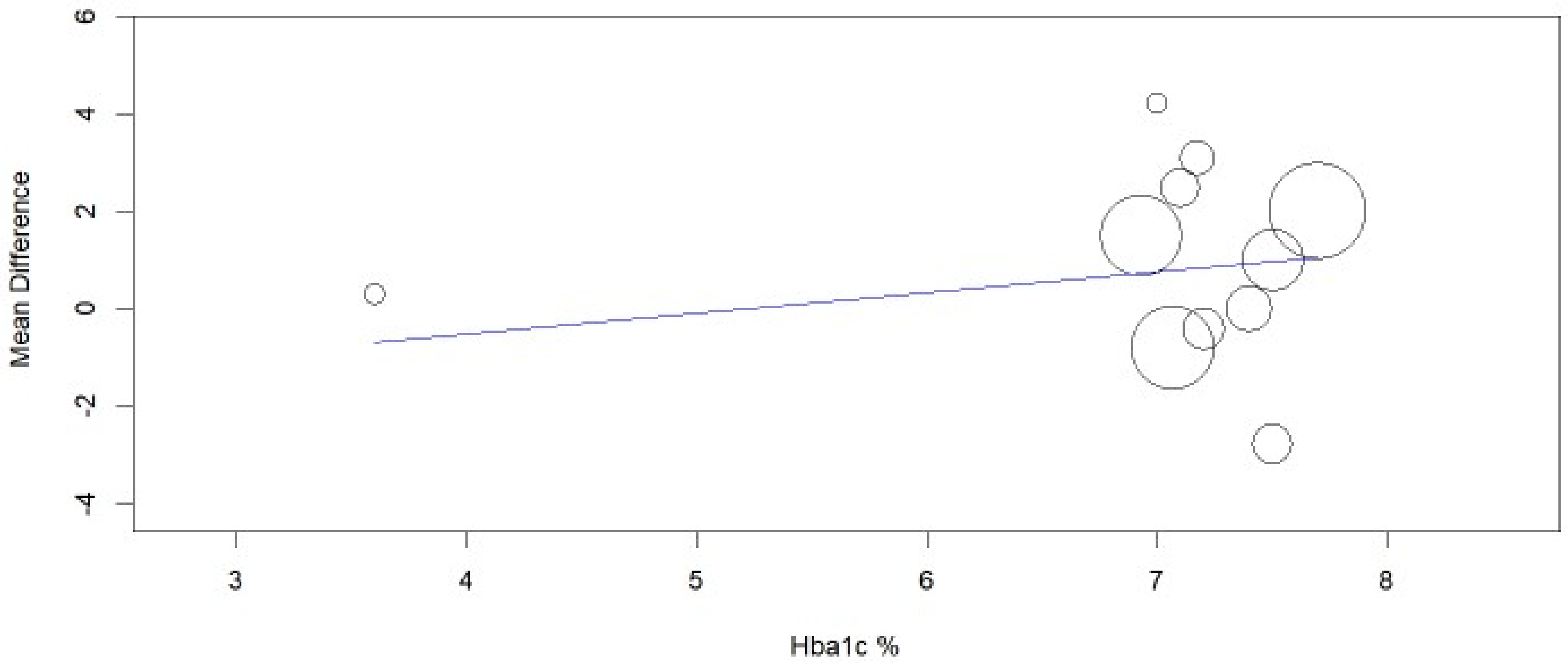

Supplementary Figure 4. TIR 70-180 mg/dl meta-regression analysis according to HbA1c

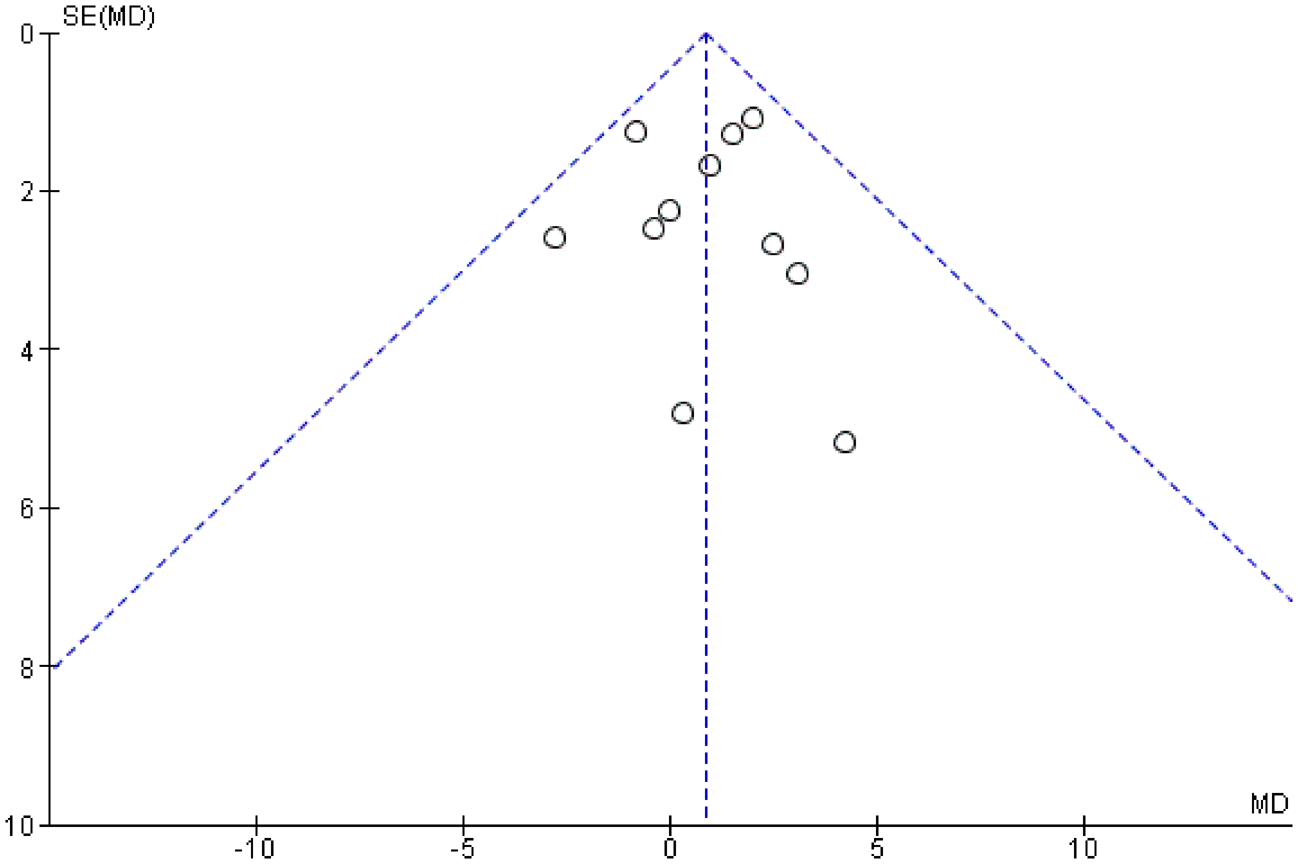

Supplementary Figure 5. TIR 70-180 mg/dl Publication bias Funnel plot

(a)

Study or Subgroup	Experimental Mean	Experimental SD	Experimental Total	Control Mean	Control SD	Control Total	Weight	Mean Difference IV, Fixed, 95% CI
Beck 2022	0.27	0.26	113	0.31	0.32	106	22.5%	-0.04 [-0.12, 0.04]
Bode 2022	0.31	0.1	42	0.42	0.1	42	74.0%	-0.11 [-0.15, -0.07]
Boughton 2021	0.4	0.47	25	0.57	0.55	25	1.7%	-0.17 [-0.45, 0.11]
Dovc 2023	0.48	0.51	30	0.64	0.65	30	1.5%	-0.16 [-0.46, 0.14]
Nwokolo 2023	0.28	0.21	26	0.33	0	27		Not estimable
Ware 2023	1.27	1.42	25	1.13	1.18	25	0.3%	0.14 [-0.58, 0.86]
**Total (95% CI)**			**261**			**255**	**100.0%**	**-0.10 [-0.13, -0.06]**

Heterogeneity: Chi² = 3.27, df = 4 (P = 0.51); I² = 0%

Test for overall effect: Z = 5.08 (P < 0.00001)

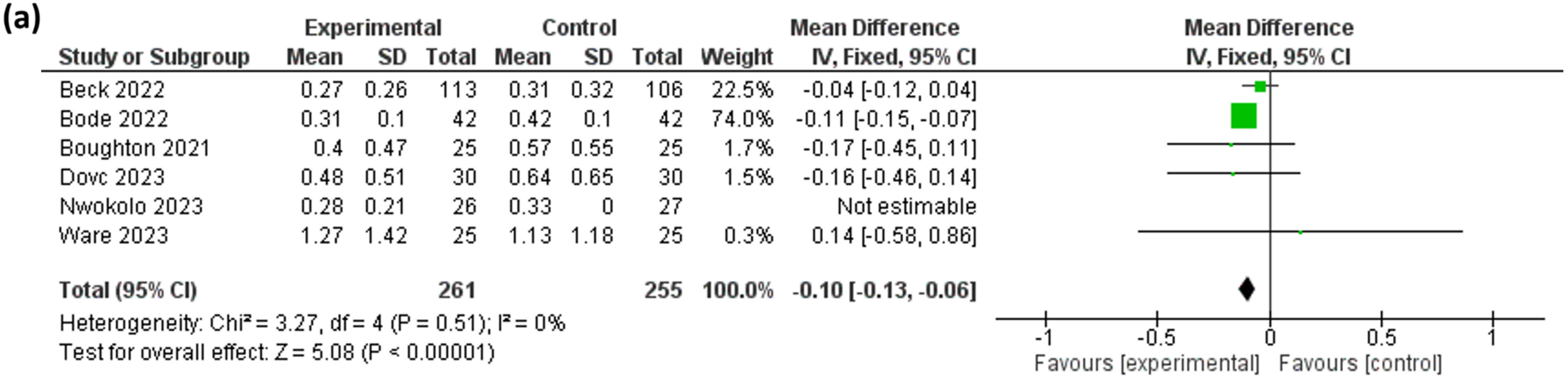

(b)

Study or Subgroup	Ultra-rapid insulin Mean	Ultra-rapid insulin SD	Ultra-rapid insulin Total	Standard insulin Mean	Standard insulin SD	Standard insulin Total	Weight	Mean Difference IV, Fixed, 95% CI
Beck 2022	0.27	0	113	0.39	0.35	106		Not estimable
Bode 2022	0.45	0.2	42	0.33	0.2	42	70.1%	0.12 [0.03, 0.21]
Boughton 2021	0.33	0	25	0.5	0.55	25		Not estimable
Dovc 2020	0	0	20	0	0	20		Not estimable
Dovc 2023	0.42	0.8	30	0.32	0.35	30	5.3%	0.10 [-0.21, 0.41]
Morrison 2022	0	0	16	0	0	16		Not estimable
Nwokolo 2023	0.22	0.34	26	0.24	0.22	27	21.4%	-0.02 [-0.17, 0.13]
Ware 2023	0.83	0.63	25	0.67	0.79	25	3.3%	0.16 [-0.24, 0.56]
**Total (95% CI)**			**297**			**291**	**100.0%**	**0.09 [0.02, 0.16]**

Heterogeneity: Chi² = 2.54, df = 3 (P = 0.47); I² = 0%

Test for overall effect: Z = 2.47 (P = 0.01)

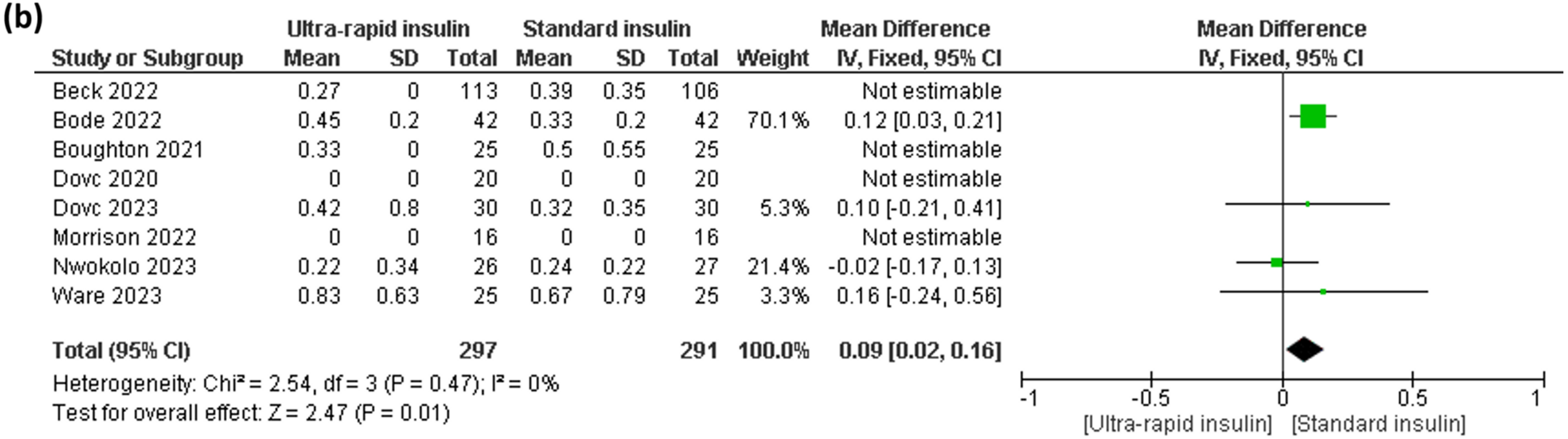

Supplementary Figure 6: Forest plots of Time Below Range (TBR): (a) Daytime TBR 54 mg/dl, (b) Nighttime TBR 54 mg/dl

(a)

Study or Subgroup	Ultra-rapid inulin Mean	SD	Total	Standard insulin Mean	SD	Total	Weight	Mean Difference IV, Random, 95% CI
Beck 2022	1.6	1.05	113	1.9	1.58	106	29.1%	-0.30 [-0.66, 0.06]
Bode 2022	1.6	0.2	42	2.4	0.2	42	43.0%	-0.80 [-0.89, -0.71]
Boughton 2021	2.5	1.97	25	3.2	2.44	25	6.2%	-0.70 [-1.93, 0.53]
Dovc 2023	2.63	2.34	30	3.03	1.71	30	8.2%	-0.40 [-1.44, 0.64]
Nwokolo 2023	2.33	1.33	26	2.5	1.8	27	11.2%	-0.17 [-1.02, 0.68]
Ware 2023	4.93	4.01	25	4.9	3.46	25	2.4%	0.03 [-2.05, 2.11]
**Total (95% CI)**			**261**			**255**	**100.0%**	**-0.53 [-0.85, -0.20]**

Heterogeneity: Tau² = 0.06; Chi² = 10.07, df = 5 (P = 0.07); I² = 50%

Test for overall effect: Z = 3.13 (P = 0.002)

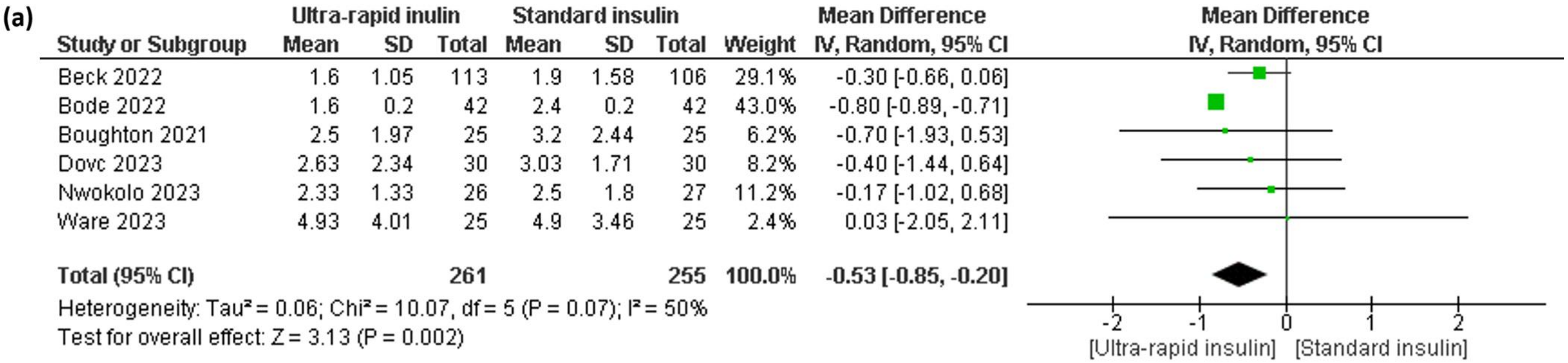

(b)

Study or Subgroup	Ultra-rapid insulin Mean	SD	Total	Standard insulin Mean	SD	Total	Weight	Mean Difference IV, Random, 95% CI
Beck 2022	1.6	1.35	113	2	1.28	106	20.8%	-0.40 [-0.75, -0.05]
Bode 2022	1.4	0.4	42	1.7	0.4	42	22.2%	-0.30 [-0.47, -0.13]
Boughton 2021	2	1.26	25	0	1.42	25	16.2%	2.00 [1.26, 2.74]
Dovc 2020	0	0	20	0	0	20		Not estimable
Dovc 2023	1.7	2.1	30	1.9	2.18	30	12.3%	-0.20 [-1.28, 0.88]
Morrison 2022	0	0	16	0	0	16		Not estimable
Nwokolo 2023	1.43	1.49	26	1.63	0.63	27	17.7%	-0.20 [-0.82, 0.42]
Ware 2023	2.93	2.12	25	2.77	2.36	25	10.7%	0.16 [-1.08, 1.40]
**Total (95% CI)**			**297**			**291**	**100.0%**	**0.13 [-0.43, 0.69]**

Heterogeneity: Tau² = 0.36; Chi² = 36.42, df = 5 (P < 0.00001); I² = 86%

Test for overall effect: Z = 0.46 (P = 0.65)

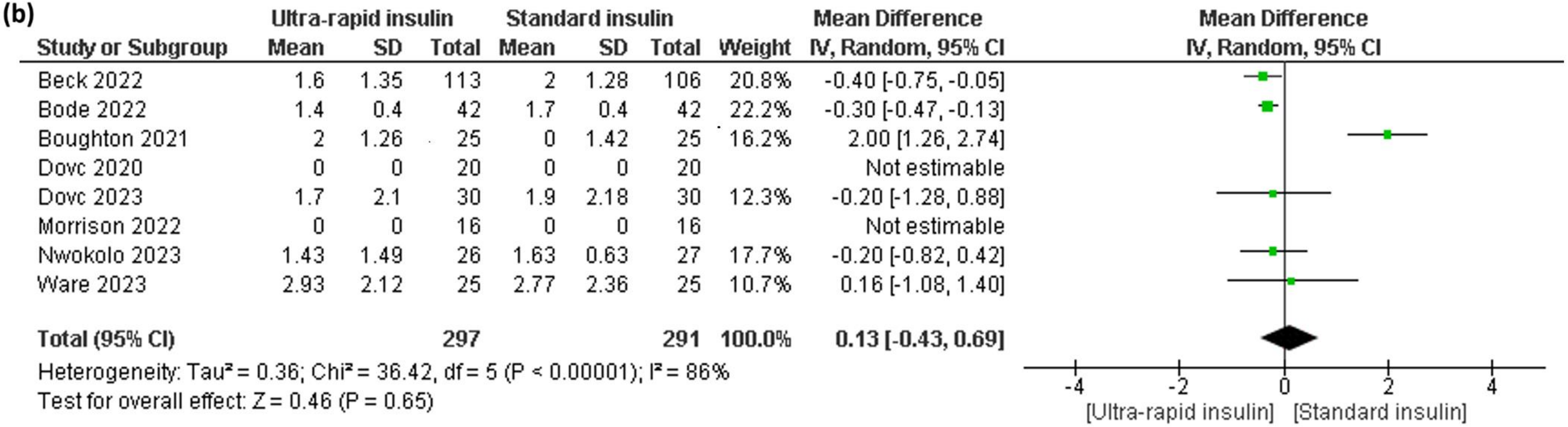

Supplementary Figure 7: Forest plots of Time Below Range (TBR): (a) Daytime TBR 70 mg/dl, (b) Nighttime TBR 70 mg/dl

	Ultra-rapid inulin			Standard insulin				Mean Difference
Study or Subgroup	Mean	SD	Total	Mean	SD	Total	Weight	IV, Random, 95% CI
Beck 2022	1.6	1.05	113	1.9	1.58	106	70.9%	-0.30 [-0.66, 0.06]
Bode 2022	1.6	0.2	42	2.4	0.2	42	0.0%	-0.80 [-0.89, -0.71]
Boughton 2021	2.5	1.97	25	3.2	2.44	25	6.0%	-0.70 [-1.93, 0.53]
Dovc 2023	2.63	2.34	30	3.03	1.71	30	8.4%	-0.40 [-1.44, 0.64]
Nwokolo 2023	2.33	1.33	26	2.5	1.8	27	12.6%	-0.17 [-1.02, 0.68]
Ware 2023	4.93	4.01	25	4.9	3.46	25	2.1%	0.03 [-2.05, 2.11]
**Total (95% CI)**			**219**			**213**	**100.0%**	**-0.31 [-0.61, -0.01]**

Heterogeneity: $Tau^2 = 0.00$; $Chi^2 = 0.63$, $df = 4$ ($P = 0.96$); $I^2 = 0\%$
Test for overall effect: $Z = 2.01$ ($P = 0.04$)

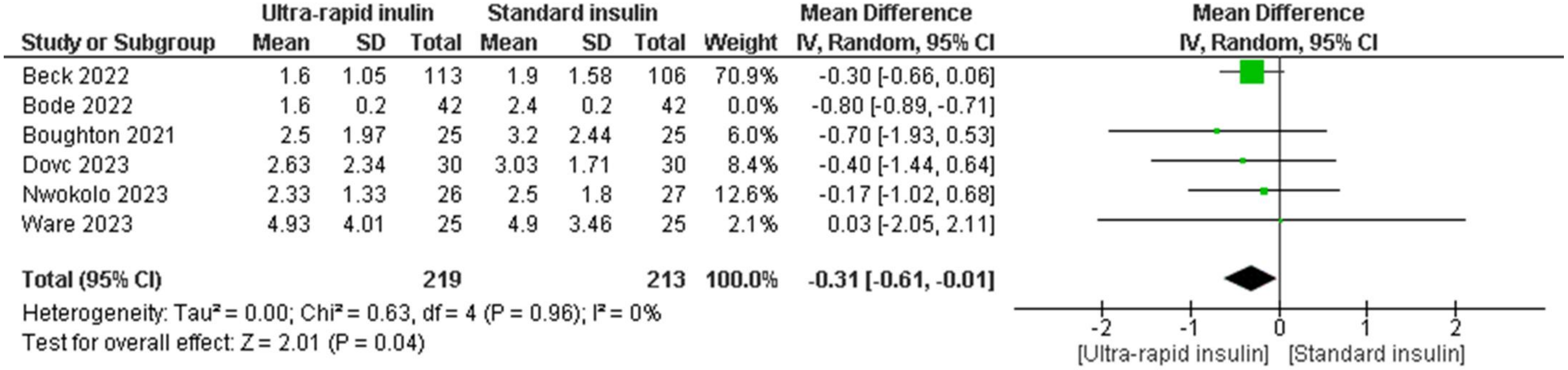

Supplementary Figure 8. TBR 70 mg/dl daytime sensitivity analysis

Study or Subgroup	Ultra-rapid insulin Mean	Ultra-rapid insulin SD	Ultra-rapid insulin Total	Standard insulin Mean	Standard insulin SD	Standard insulin Total	Weight	Mean Difference IV, Random, 95% CI
Beck 2022	1.6	1.35	113	2	1.28	106	17.7%	-0.40 [-0.75, -0.05]
Bode 2022	1.4	0.4	42	1.7	0.4	42	73.5%	-0.30 [-0.47, -0.13]
Boughton 2021	2	1.26	25	0	1.42	25	0.0%	2.00 [1.26, 2.74]
Dovc 2020	0	0	20	0	0	20		Not estimable
Dovc 2023	1.7	2.1	30	1.9	2.18	30	1.8%	-0.20 [-1.28, 0.88]
Morrison 2022	0	0	16	0	0	16		Not estimable
Nwokolo 2023	1.43	1.49	26	1.63	0.63	27	5.6%	-0.20 [-0.82, 0.42]
Ware 2023	2.93	2.12	25	2.77	2.36	25	1.4%	0.16 [-1.08, 1.40]
**Total (95% CI)**			**272**			**266**	**100.0%**	**-0.30 [-0.45, -0.16]**

Heterogeneity: Tau² = 0.00; Chi² = 0.97, df = 4 (P = 0.91); I² = 0%

Test for overall effect: Z = 4.06 (P < 0.0001)

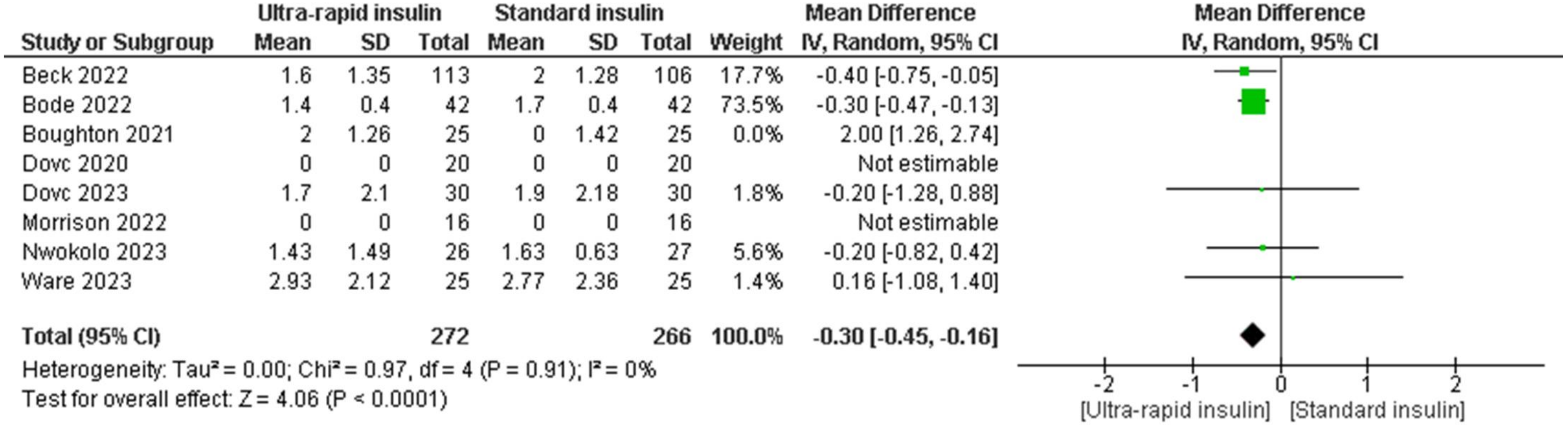

Supplementary Figure 9. TBR 70 mg/dl nighttime sensitivity analysis

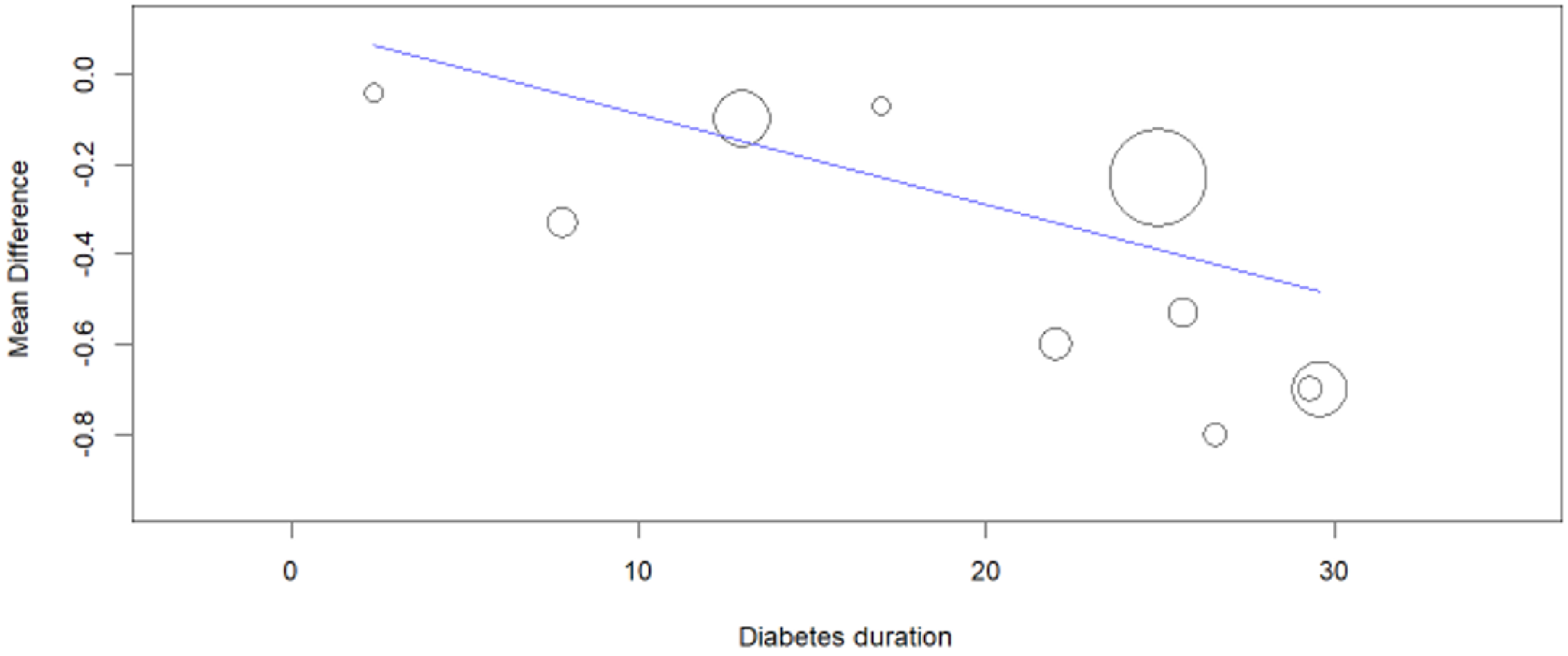

Supplementary Figure 10. TBR 70 mg/dl meta-regression analysis according to diabetes duration

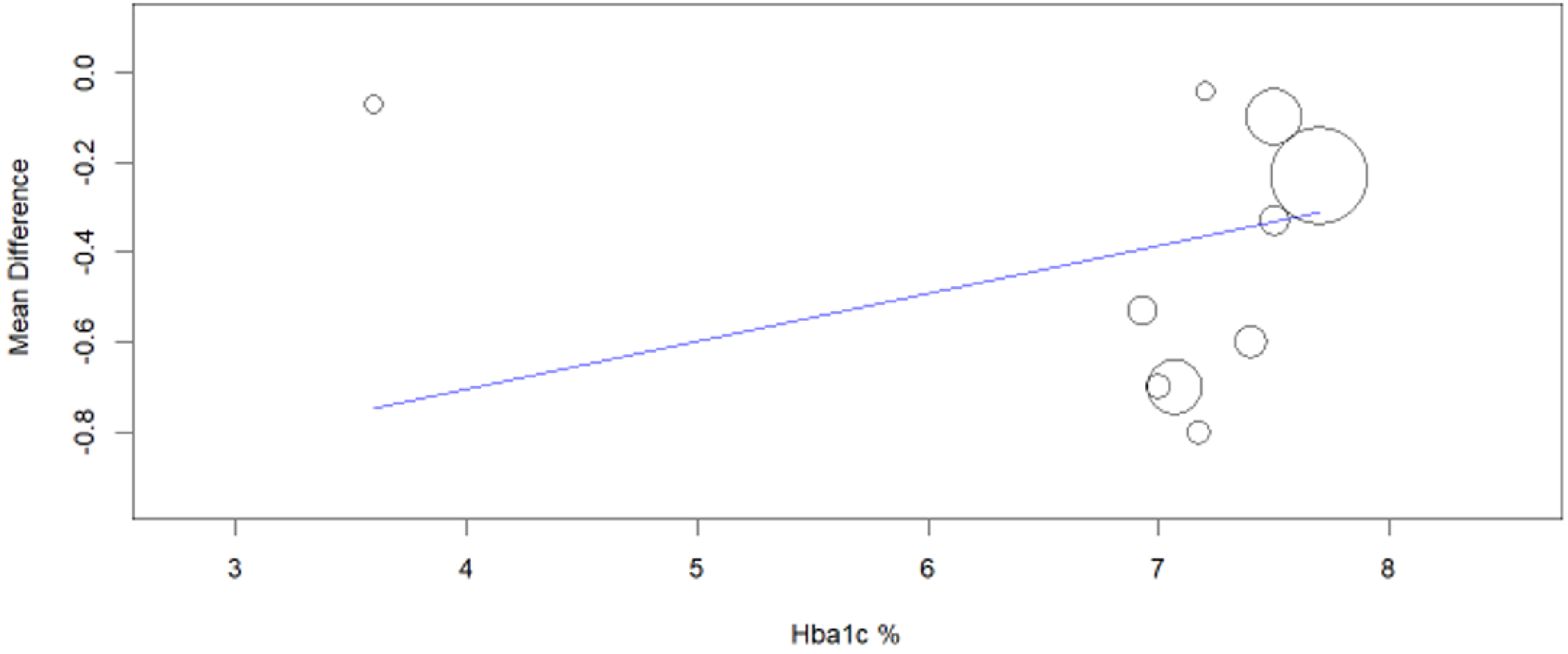

Supplementary Figure 11. TBR 70 mg/dl meta-regression analysis according to HbA1c

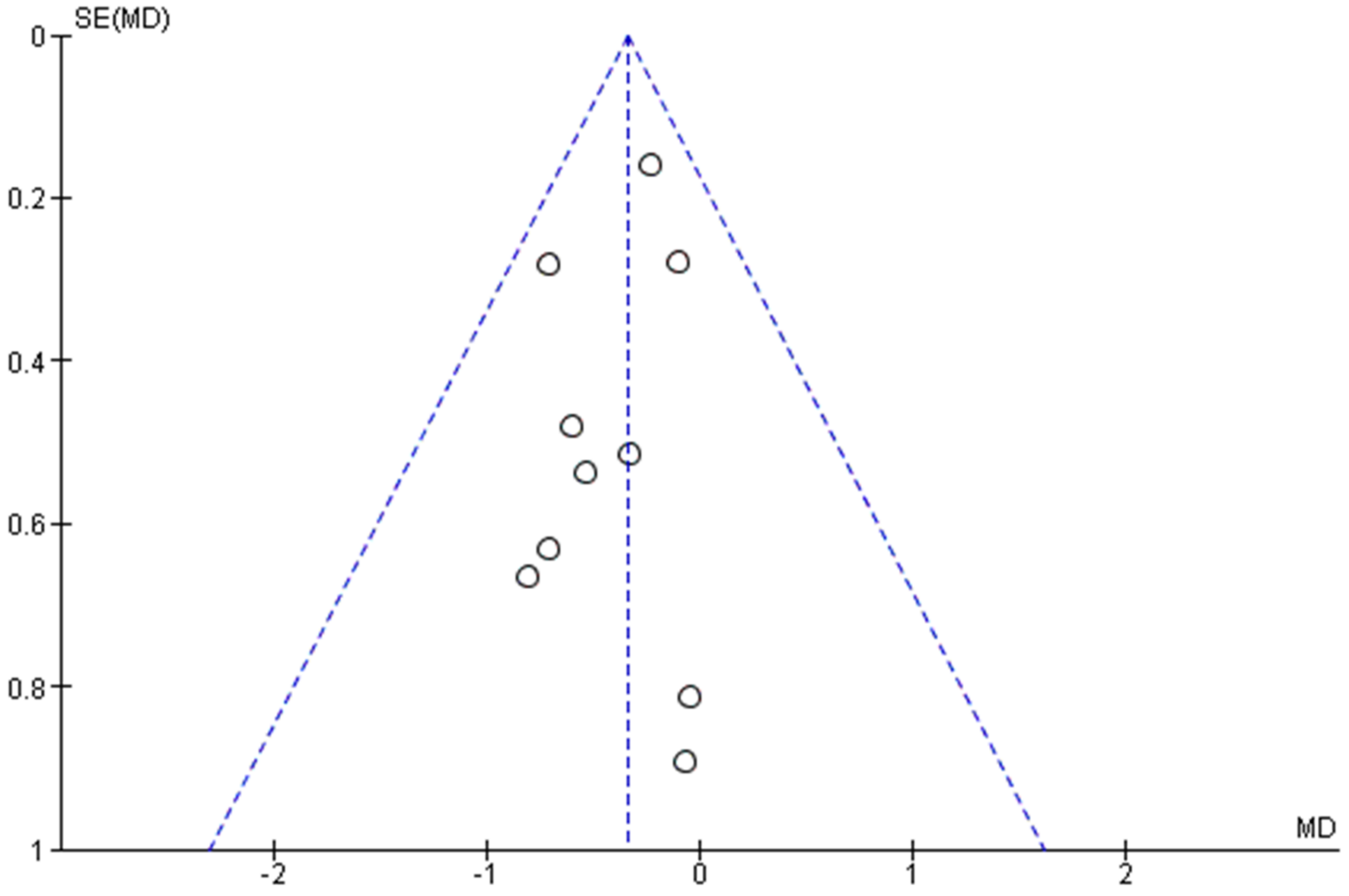

Supplementary Figure 12. TBR 70 mg/dl Publication bias Funnel plot

(a)

Study or Subgroup	Ultra-rapid insulin Mean	SD	Total	Standard insulin Mean	SD	Total	Weight	Mean Difference IV, Random, 95% CI
Beck 2022	5.37	3.6	113	6.43	3.83	106	33.4%	-1.06 [-2.05, -0.07]
Bode 2022	4.1	0.4	42	3.4	0.4	42	35.2%	0.70 [0.53, 0.87]
Dovc 2023	0	2.26	30	4.17	3.43	30	31.4%	-4.17 [-5.64, -2.70]
**Total (95% CI)**			**185**			**178**	**100.0%**	**-1.42 [-3.90, 1.07]**

Heterogeneity: Tau² = 4.57; Chi² = 52.63, df = 2 (P < 0.00001); I² = 96%

Test for overall effect: Z = 1.12 (P = 0.26)

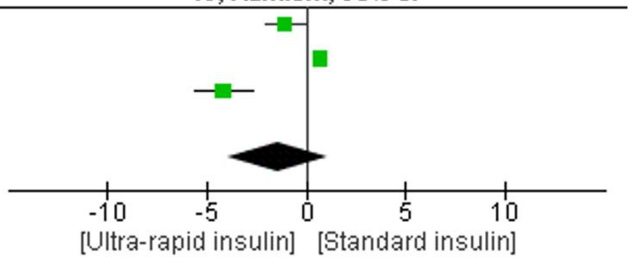

(b)

Study or Subgroup	Ultra-rapid insulin Mean	SD	Total	Standard insulin Mean	SD	Total	Weight	Mean Difference IV, Fixed, 95% CI
Beck 2022	3.5	3.38	113	3.6	3.76	106	6.6%	-0.10 [-1.05, 0.85]
Bode 2022	2.9	0.6	42	2.5	0.6	42	90.0%	0.40 [0.14, 0.66]
Dovc 2023	1.3	2.49	30	1.4	2.72	30	3.4%	-0.10 [-1.42, 1.22]
Morrison 2022	0	0	16	0	0	16		Not estimable
**Total (95% CI)**			**201**			**194**	**100.0%**	**0.35 [0.11, 0.59]**

Heterogeneity: Chi² = 1.46, df = 2 (P = 0.48); I² = 0%

Test for overall effect: Z = 2.82 (P = 0.005)

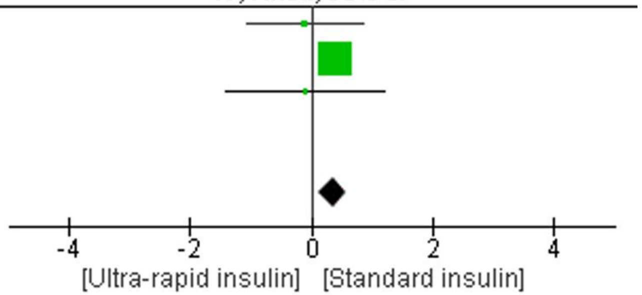

Supplementary Figure 13. Forest plots of Time Above Range (TAR): (a) Daytime TAR 250 mg/dl, (b) Nighttime TAR 250 mg/dl

(a)

Study or Subgroup	Ultra-rapid insulin Mean	SD	Total	Standard insulin Mean	SD	Total	Weight	Mean Difference IV, Random, 95% CI
Beck 2022	28	8	113	30	8	106	23.8%	-2.00 [-4.12, 0.12]
Bode 2022	23.2	1	42	21.9	1	42	35.4%	1.30 [0.87, 1.73]
Boughton 2021	22	9	25	22	9	25	9.3%	0.00 [-4.99, 4.99]
Dovc 2023	22	7	30	23	7	30	14.8%	-1.00 [-4.54, 2.54]
Nwokolo 2023	20.6	10.5	26	22.5	10.4	27	7.8%	-1.90 [-7.53, 3.73]
Ware 2023	31.7	9.2	25	31.5	9.3	25	9.0%	0.20 [-4.93, 5.33]
**Total (95% CI)**			**261**			**255**	**100.0%**	**-0.29 [-2.06, 1.48]**

Heterogeneity: Tau² = 2.25; Chi² = 11.80, df = 5 (P = 0.04); I² = 58%
Test for overall effect: Z = 0.32 (P = 0.75)

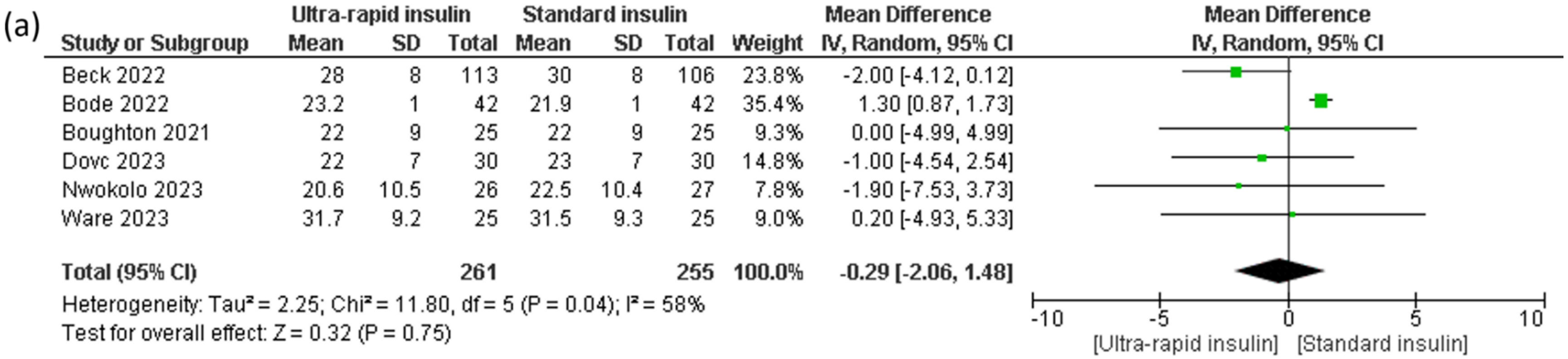

(b)

Study or Subgroup	Ultra-rapid insulin Mean	SD	Total	Standard insulin Mean	SD	Total	Weight	Mean Difference IV, Fixed, 95% CI
Beck 2022	23	11	113	23	12	106	3.0%	0.00 [-3.05, 3.05]
Bode 2022	16.4	1.3	42	14	1.3	42	91.9%	2.40 [1.84, 2.96]
Boughton 2021	22	11	25	21	10	25	0.8%	1.00 [-4.83, 6.83]
Dovc 2023	10	8	30	10	6	30	2.2%	0.00 [-3.58, 3.58]
Morrison 2022	8.33	20.32	16	6.25	15.24	16	0.2%	2.08 [-10.37, 14.53]
Nwokolo 2023	15.2	9.2	26	18.5	10.4	27	1.0%	-3.30 [-8.58, 1.98]
Ware 2023	30.7	11.5	25	30.2	10.2	25	0.8%	0.50 [-5.53, 6.53]
**Total (95% CI)**			**277**			**271**	**100.0%**	**2.19 [1.66, 2.72]**

Heterogeneity: Chi² = 8.57, df = 6 (P = 0.20); I² = 30%
Test for overall effect: Z = 8.05 (P < 0.00001)

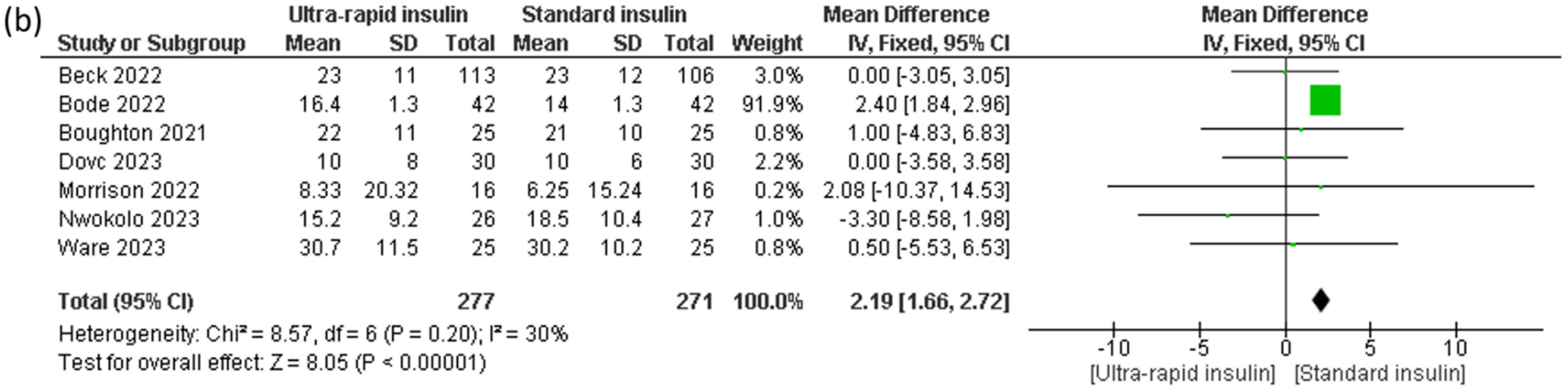

Supplementary Figure 14. Forest plots of Time Above Range (TAR): (a) Daytime TAR 180 mg/dl, (b) Nighttime TAR 180 mg/dl

	Ultra-rapid insulin			Standard insulin				Mean Difference
Study or Subgroup	Mean	SD	Total	Mean	SD	Total	Weight	IV, Random, 95% CI
Beck 2022	5	3.6	114	5.5	3.2	107	35.2%	-0.50 [-1.40, 0.40]
Bode 2022	3.8	2.59	42	3.2	2.59	42	23.1%	0.60 [-0.51, 1.71]
Dovc 2020	7.23	10.21	20	9.53	12.05	20	0.6%	-2.30 [-9.22, 4.62]
Dovc 2023	4.8	3.53	30	9.97	11.9	30	0.0%	-5.17 [-9.61, -0.73]
Lee 2021	1.9	1.1	25	2.3	1.81	25	41.1%	-0.40 [-1.23, 0.43]
Morrison 2022	0	0	16	0.52	1.28	16		Not estimable
**Total (95% CI)**			**217**			**210**	**100.0%**	**-0.22 [-0.75, 0.32]**

Heterogeneity: Tau² = 0.00; Chi² = 3.01, df = 3 (P = 0.39); I² = 0%
Test for overall effect: Z = 0.79 (P = 0.43)

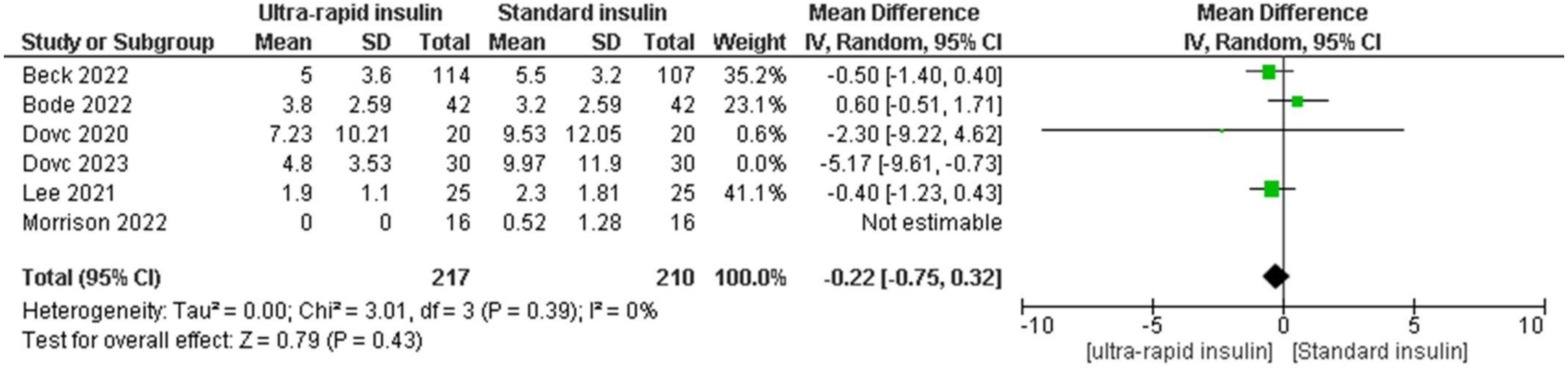

Supplementary Figure 15. All day TAR 250 mg/dl sensitivity analysis

Study or Subgroup	Ultra-rapid insulin Mean	Ultra-rapid insulin SD	Ultra-rapid insulin Total	Standard insulin Mean	Standard insulin SD	Standard insulin Total	Weight	Mean Difference IV, Random, 95% CI
Beck 2022	28	8	113	30	8	106	54.0%	-2.00 [-4.12, 0.12]
Bode 2022	23.2	1	42	21.9	1	42	0.0%	1.30 [0.87, 1.73]
Boughton 2021	22	9	25	22	9	25	9.8%	0.00 [-4.99, 4.99]
Dovc 2023	22	7	30	23	7	30	19.3%	-1.00 [-4.54, 2.54]
Nwokolo 2023	20.6	10.5	26	22.5	10.4	27	7.7%	-1.90 [-7.53, 3.73]
Ware 2023	31.7	9.2	25	31.5	9.3	25	9.2%	0.20 [-4.93, 5.33]
**Total (95% CI)**			**219**			**213**	**100.0%**	**-1.40 [-2.96, 0.16]**

Heterogeneity: $Tau^2 = 0.00$; $Chi^2 = 1.06$, df = 4 (P = 0.90); $I^2 = 0\%$

Test for overall effect: Z = 1.76 (P = 0.08)

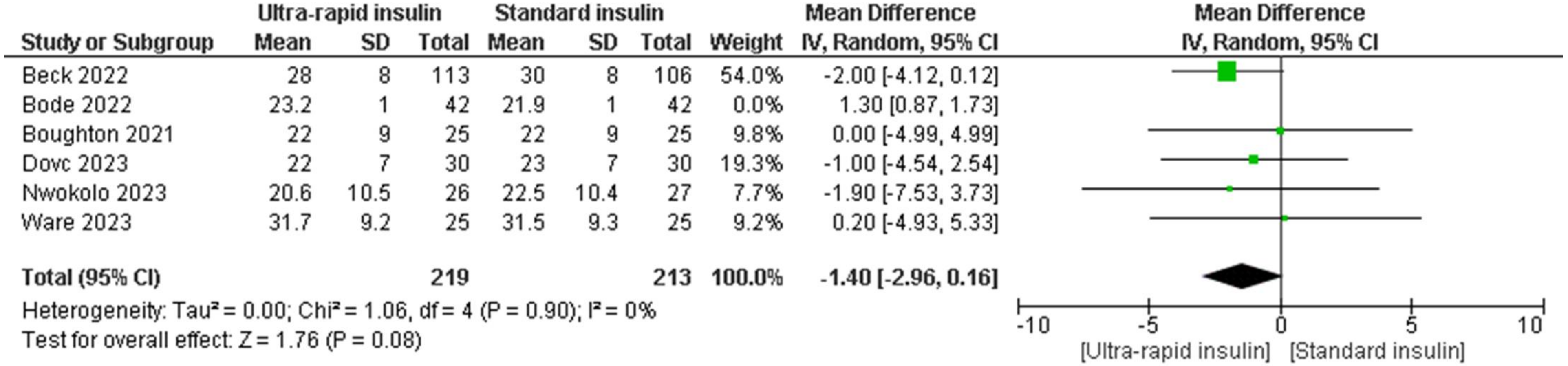

Supplementary Figure 16. Daytime TAR 180 mg/dl sensitivity analysis

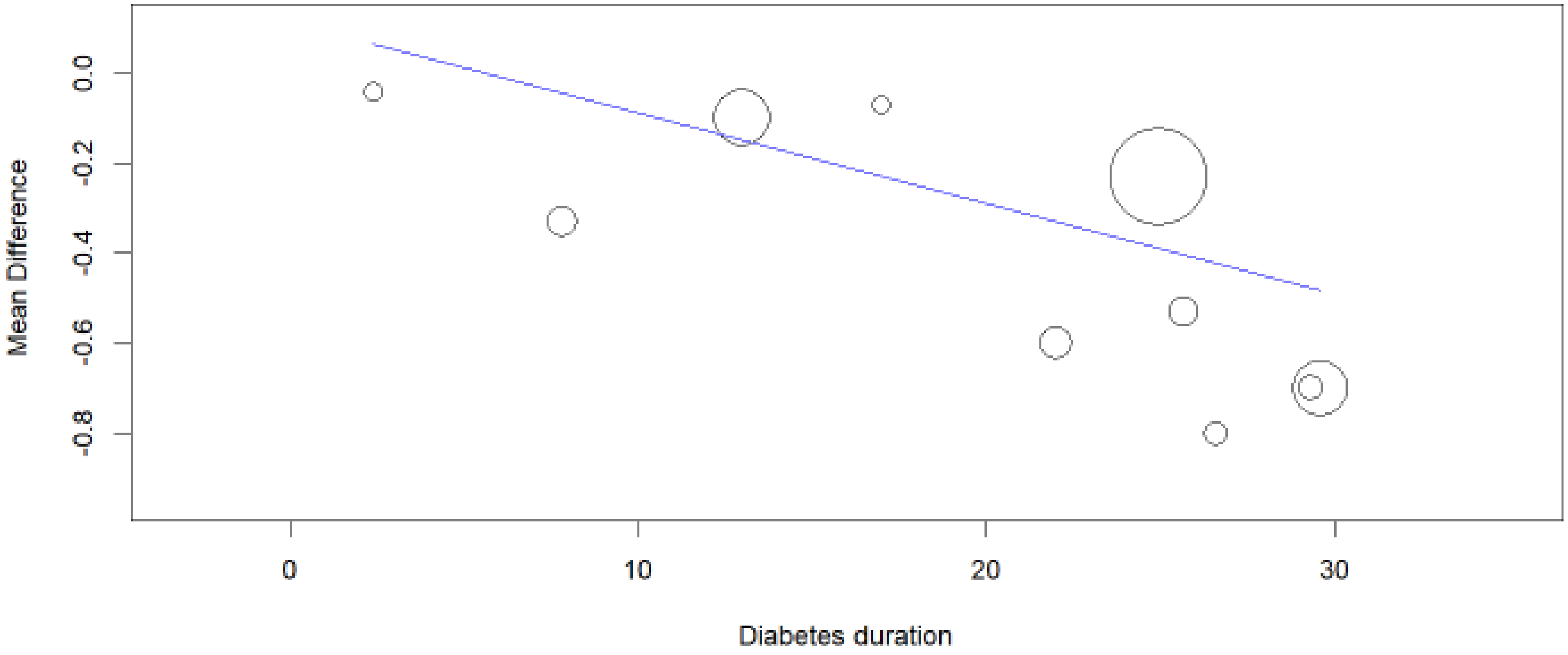

Supplementary Figure 17. TAR 180 mg/dl meta-regression analysis according to diabetes duration

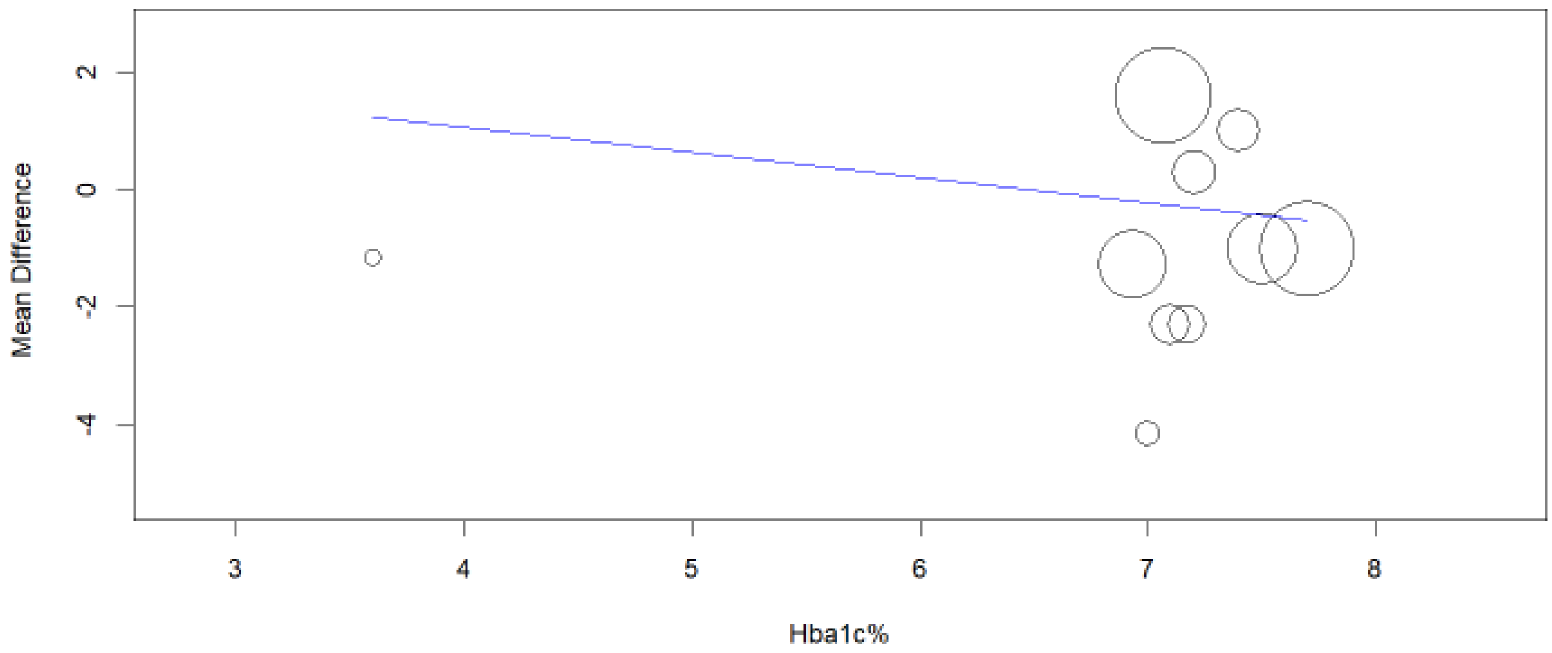

Supplementary Figure 18. TAR 180 mg/dl meta-regression analysis according to HbA1c

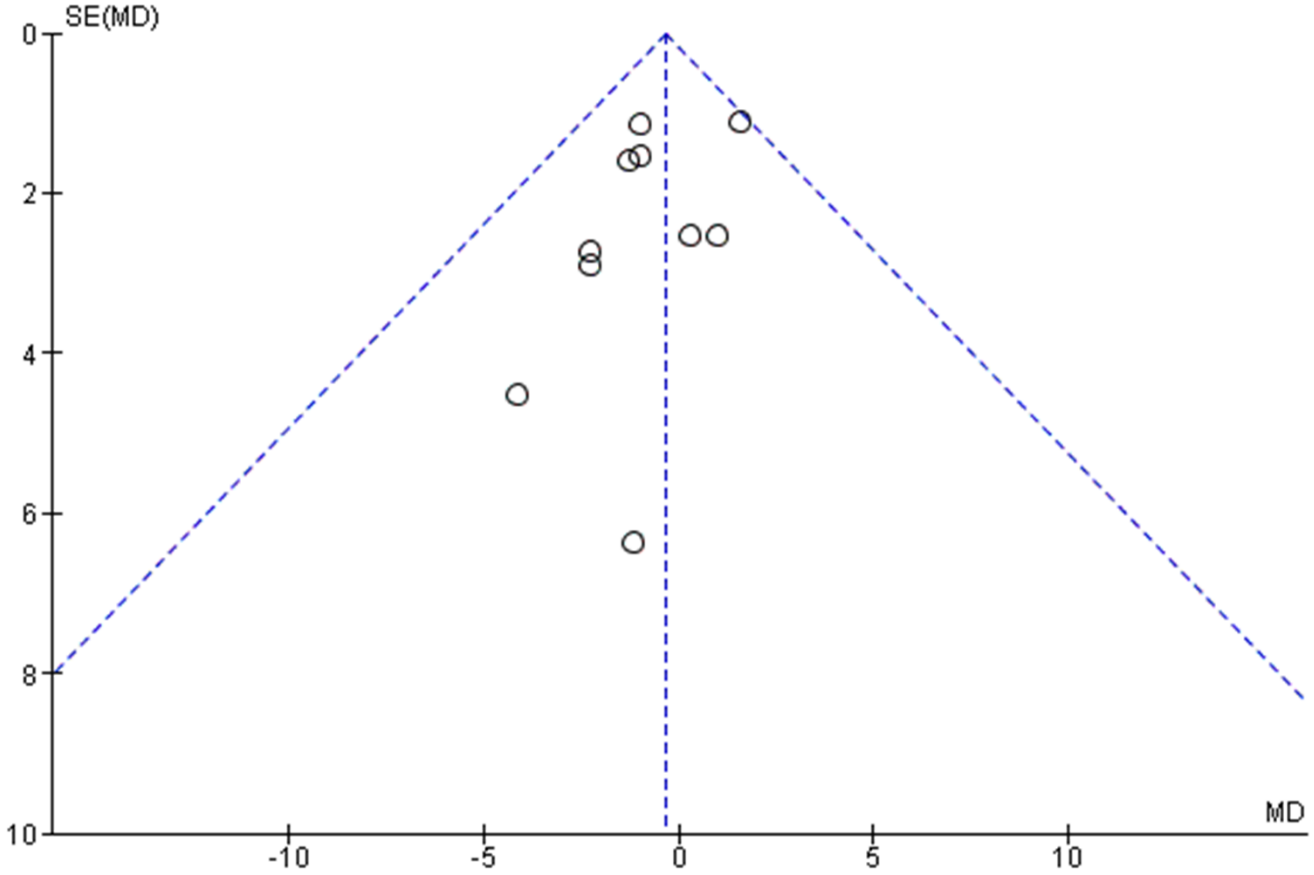

Supplementary Figure 19. TBR 180 mg/dl Publication bias Funnel plot

(a)

Study or Subgroup	Ultra-rapid insulin Mean	SD	Total	Standard insulin Mean	SD	Total	Weight	Mean Difference IV, Fixed, 95% CI
Beck 2022	157	11	113	159	12	106	69.5%	-2.00 [-5.05, 1.05]
Boughton 2021	144.144	14.414	25	144.144	0	25		Not estimable
Dovc 2023	126	15	30	126	12	30	13.7%	0.00 [-6.87, 6.87]
Nwokolo 2023	144	16	26	146	16	27	8.7%	-2.00 [-10.62, 6.62]
Ware 2023	160.36	16.22	25	158.558	16.216	25	8.0%	1.80 [-7.19, 10.79]
**Total (95% CI)**			**219**			**213**	**100.0%**	**-1.42 [-3.97, 1.13]**

Heterogeneity: Chi² = 0.81, df = 3 (P = 0.85); I² = 0%

Test for overall effect: Z = 1.09 (P = 0.27)

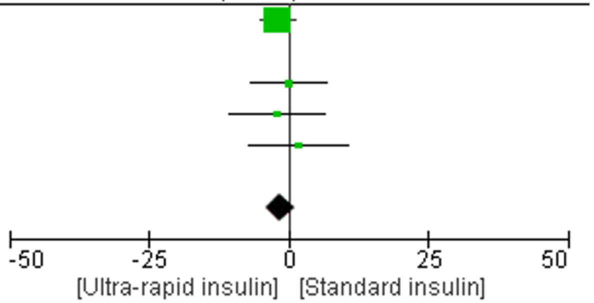

(b)

Study or Subgroup	Ultra-rapid insulin Mean	SD	Total	Standard insulin Mean	SD	Total	Weight	Mean Difference IV, Random, 95% CI
Beck 2022	150	15	113	150	17	106	14.4%	0.00 [-4.26, 4.26]
Boughton 2021	145.9	16.216	25	144.144	16.216	25	14.2%	1.76 [-7.23, 10.75]
Dovc 2020	140.2	22.9	20	133.63	15.64	20	14.1%	6.57 [-5.58, 18.72]
Dovc 2023	126	15	30	126	12	30	14.3%	0.00 [-6.87, 6.87]
Morrison 2022	6.95	1.64	16	6.86	1.89	16	14.4%	0.09 [-1.14, 1.32]
Nwokolo 2023	139	14	26	142	16	27	14.3%	-3.00 [-11.09, 5.09]
Ware 2023	160.36	18.018	25	0	14.414	25	14.2%	160.36 [151.32, 169.40]
**Total (95% CI)**			**255**			**249**	**100.0%**	**23.59 [-4.19, 51.36]**

Heterogeneity: Tau² = 1389.42; Chi² = 1189.02, df = 6 (P < 0.00001); I² = 99%

Test for overall effect: Z = 1.66 (P = 0.10)

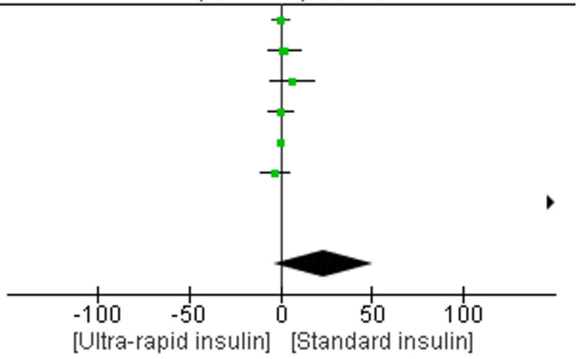

Supplementary Figure 20. Forest plots of glycemic variability: (a) Daytime mean glucose, (b) Nighttime mean glucose

(a)

Study or Subgroup	Ultra-rapid insulin Mean	SD	Total	Standard insulin Mean	SD	Total	Weight	Mean Difference IV, Random, 95% CI
Beck 2022	33	4	113	35	4	106	41.8%	-2.00 [-3.06, -0.94]
Boughton 2021	36	4	25	36	3	25	19.4%	0.00 [-1.96, 1.96]
Dovc 2023	35	3.11	30	36.67	6.23	30	13.2%	-1.67 [-4.16, 0.82]
Nwokolo 2023	35.2	4.7	26	35.5	4.2	27	14.1%	-0.30 [-2.70, 2.10]
Ware 2023	42.9	4.8	25	42.7	5	25	11.4%	0.20 [-2.52, 2.92]
**Total (95% CI)**			**219**			**213**	**100.0%**	**-1.08 [-2.07, -0.08]**

Heterogeneity: Tau² = 0.32; Chi² = 5.28, df = 4 (P = 0.26); I² = 24%
Test for overall effect: Z = 2.13 (P = 0.03)

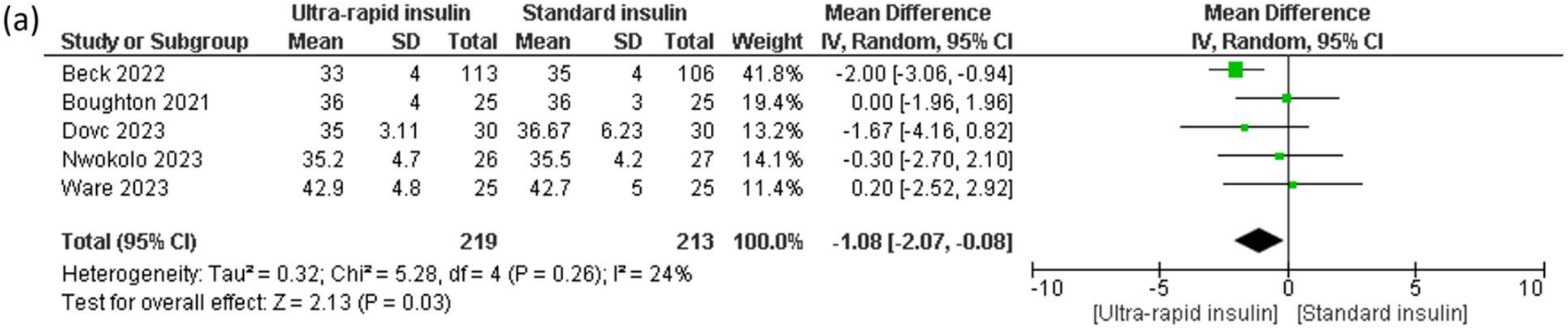

(b)

Study or Subgroup	Ultra-rapid insulin Mean	SD	Total	Standard insulin Mean	SD	Total	Weight	Mean Difference IV, Fixed, 95% CI
Beck 2022	31	6	113	32	5	106	53.3%	-1.00 [-2.46, 0.46]
Boughton 2021	35.5	5	25	36	5	25	14.8%	-0.50 [-3.27, 2.27]
Dovc 2023	29.67	6.23	30	29.67	7.01	30	10.1%	0.00 [-3.36, 3.36]
Morrison 2022	16.94	8.06	16	14.32	10.48	16	2.7%	2.62 [-3.86, 9.10]
Nwokolo 2023	32.1	7.2	26	34.5	7.3	27	7.4%	-2.40 [-6.30, 1.50]
Ware 2023	39.4	5.2	25	39.4	6	25	11.7%	0.00 [-3.11, 3.11]
**Total (95% CI)**			**235**			**229**	**100.0%**	**-0.71 [-1.78, 0.35]**

Heterogeneity: Chi² = 2.28, df = 5 (P = 0.81); I² = 0%
Test for overall effect: Z = 1.31 (P = 0.19)

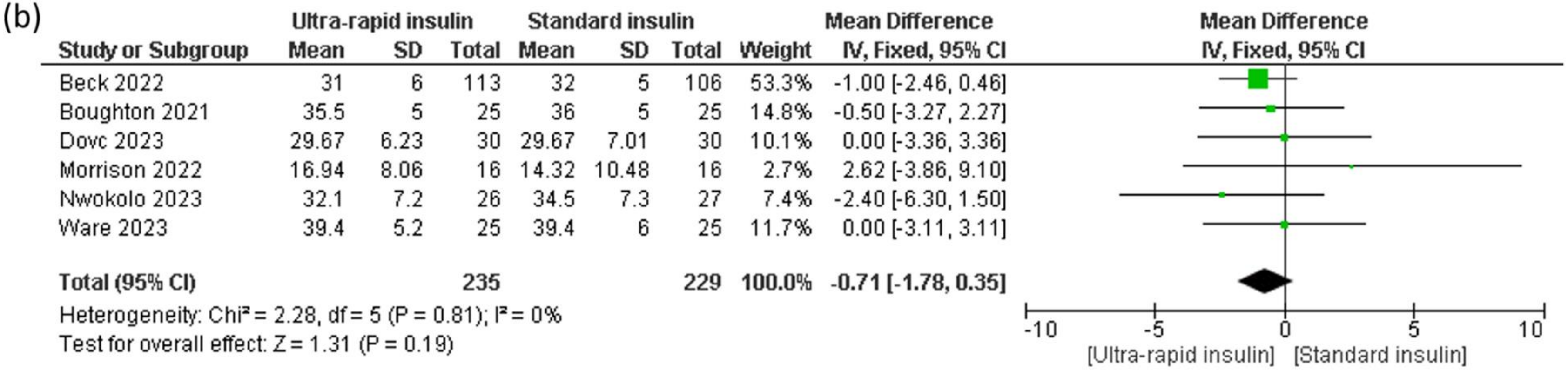

Supplementary Figure 21. Forest plots of glycemic variability: (a) Daytime CV, (b) Nighttime CV

Study or Subgroup	Ultra-rapid insulin Mean	SD	Total	Standard insulin Mean	SD	Total	Weight	Mean Difference IV, Random, 95% CI
Beck 2022	150	15	113	150	17	106	7.1%	0.00 [-4.26, 4.26]
Boughton 2021	145.9	16.216	25	144.144	16.216	25	1.6%	1.76 [-7.23, 10.75]
Dovc 2020	140.2	22.9	20	133.63	15.64	20	0.9%	6.57 [-5.58, 18.72]
Dovc 2023	126	15	30	126	12	30	2.7%	0.00 [-6.87, 6.87]
Morrison 2022	6.95	1.64	16	6.86	1.89	16	85.7%	0.09 [-1.14, 1.32]
Nwokolo 2023	139	14	26	142	16	27	2.0%	-3.00 [-11.09, 5.09]
Ware 2023	160.36	18.018	25	0	14.414	25	0.0%	160.36 [151.32, 169.40]
**Total (95% CI)**			**230**			**224**	**100.0%**	**0.10 [-1.03, 1.24]**

Heterogeneity: Tau² = 0.00; Chi² = 1.79, df = 5 (P = 0.88); I² = 0%
Test for overall effect: Z = 0.18 (P = 0.86)

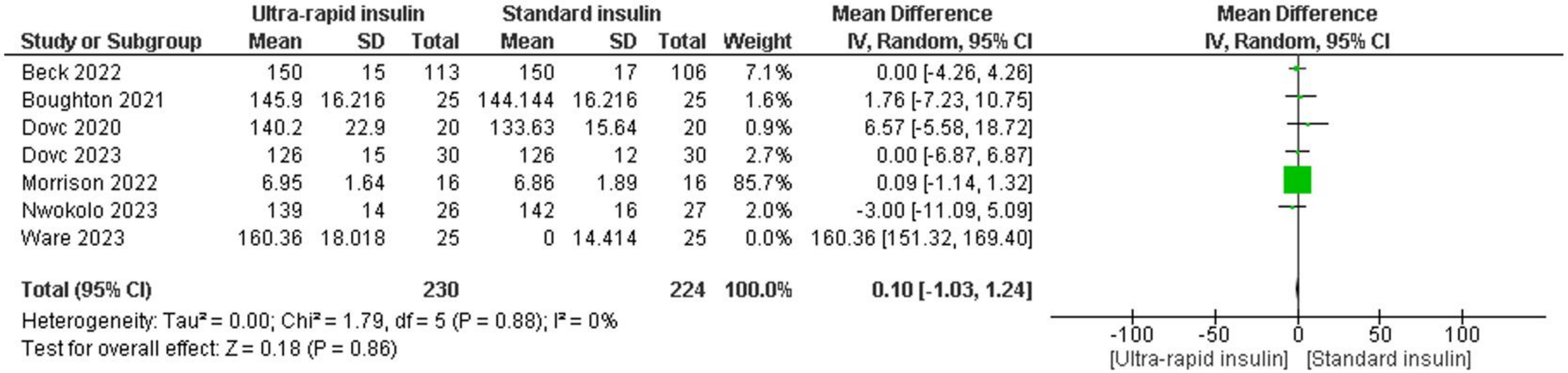

Supplementary Figure 22. Nighttime mean glucose sensitivity analysis

	Ultra-rapid insulin			Standard insulin				Mean Difference
**Study or Subgroup**	**Mean**	**SD**	**Total**	**Mean**	**SD**	**Total**	**Weight**	**IV, Random, 95% CI**
Beck 2022	33	4	113	35	4	106	0.0%	-2.00 [-3.06, -0.94]
Boughton 2021	36	4	25	36	3	25	35.7%	0.00 [-1.96, 1.96]
Dovc 2023	35	3.11	30	36.67	6.23	30	22.1%	-1.67 [-4.16, 0.82]
Nwokolo 2023	35.2	4.7	26	35.5	4.2	27	23.7%	-0.30 [-2.70, 2.10]
Ware 2023	42.9	4.8	25	42.7	5	25	18.6%	0.20 [-2.52, 2.92]
**Total (95% CI)**			**106**			**107**	**100.0%**	**-0.40 [-1.57, 0.77]**

Heterogeneity: Tau² = 0.00; Chi² = 1.35, df = 3 (P = 0.72); I² = 0%
Test for overall effect: Z = 0.67 (P = 0.50)

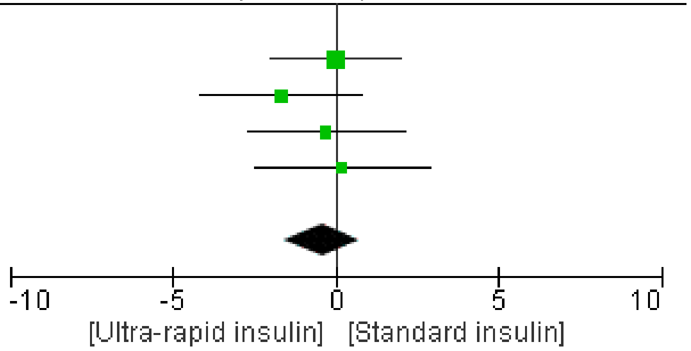

Supplementary Figure 23. Daytime CV sensitivity analysis

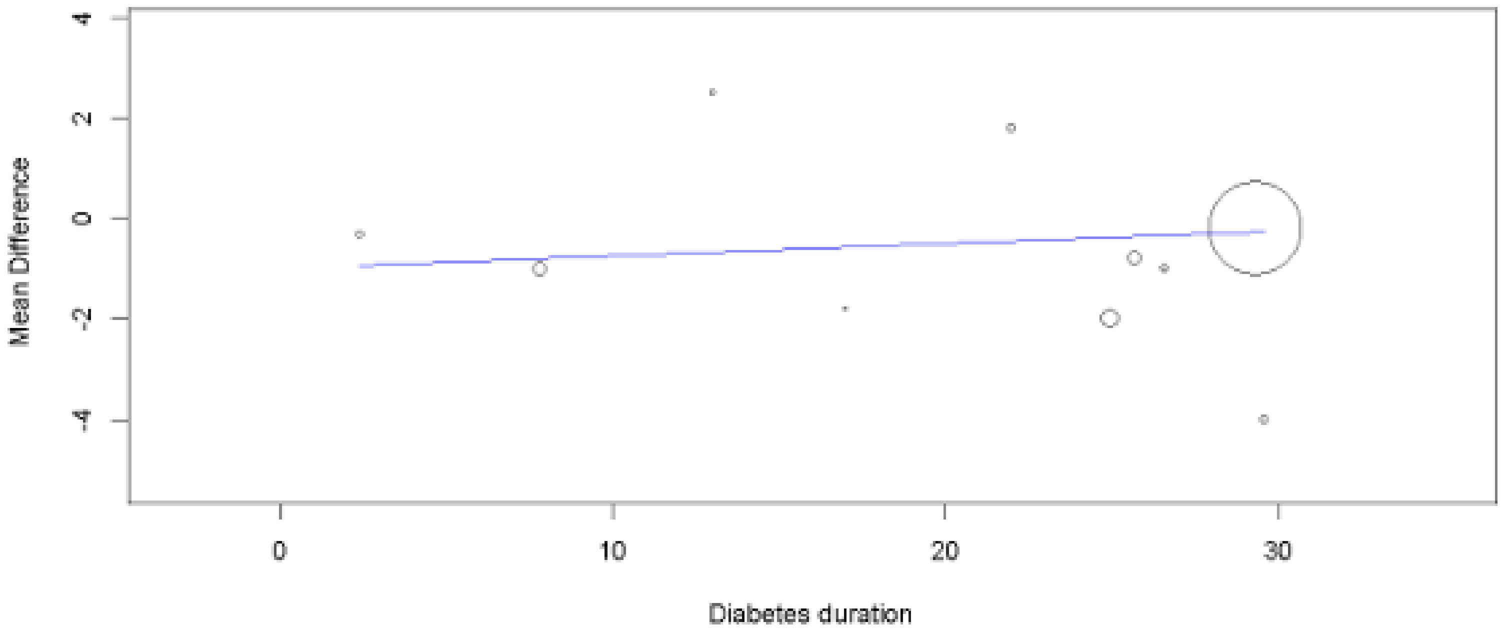

Supplementary Figure 24. Mean Glucose meta-regression analysis according to diabetes duration

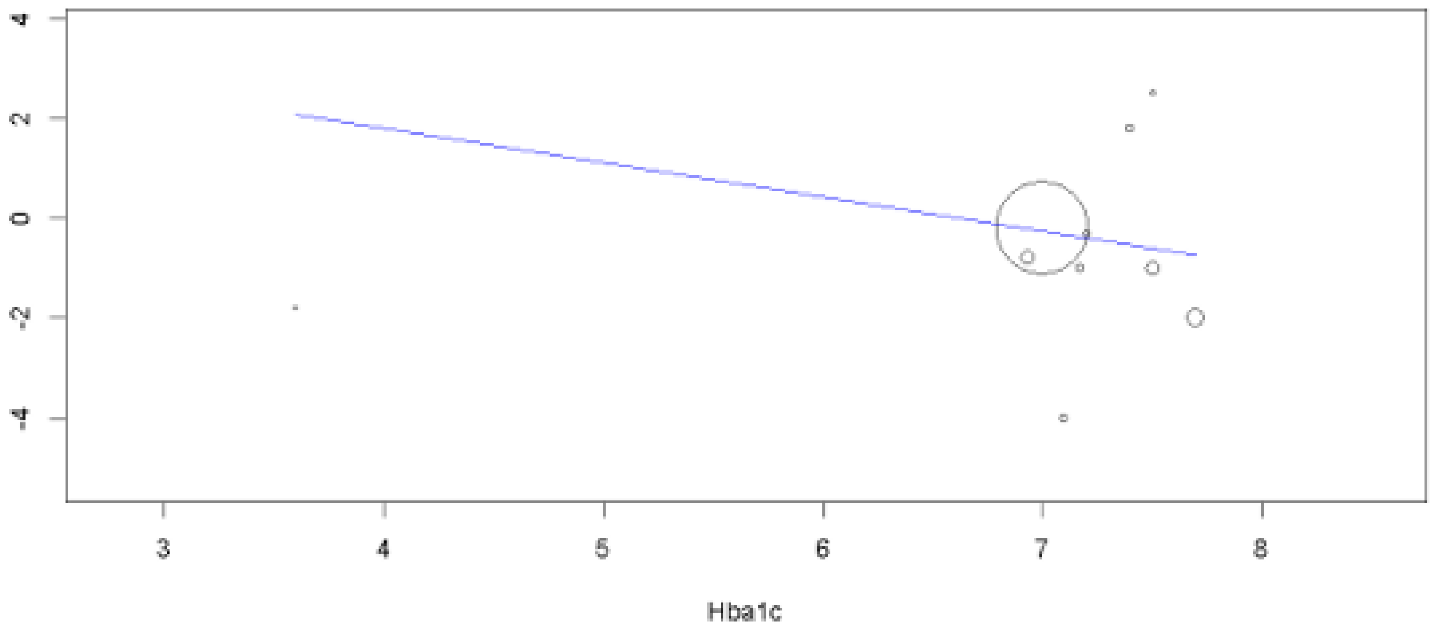

Supplementary Figure 25. Mean Glucose meta-regression analysis according to HbA1c

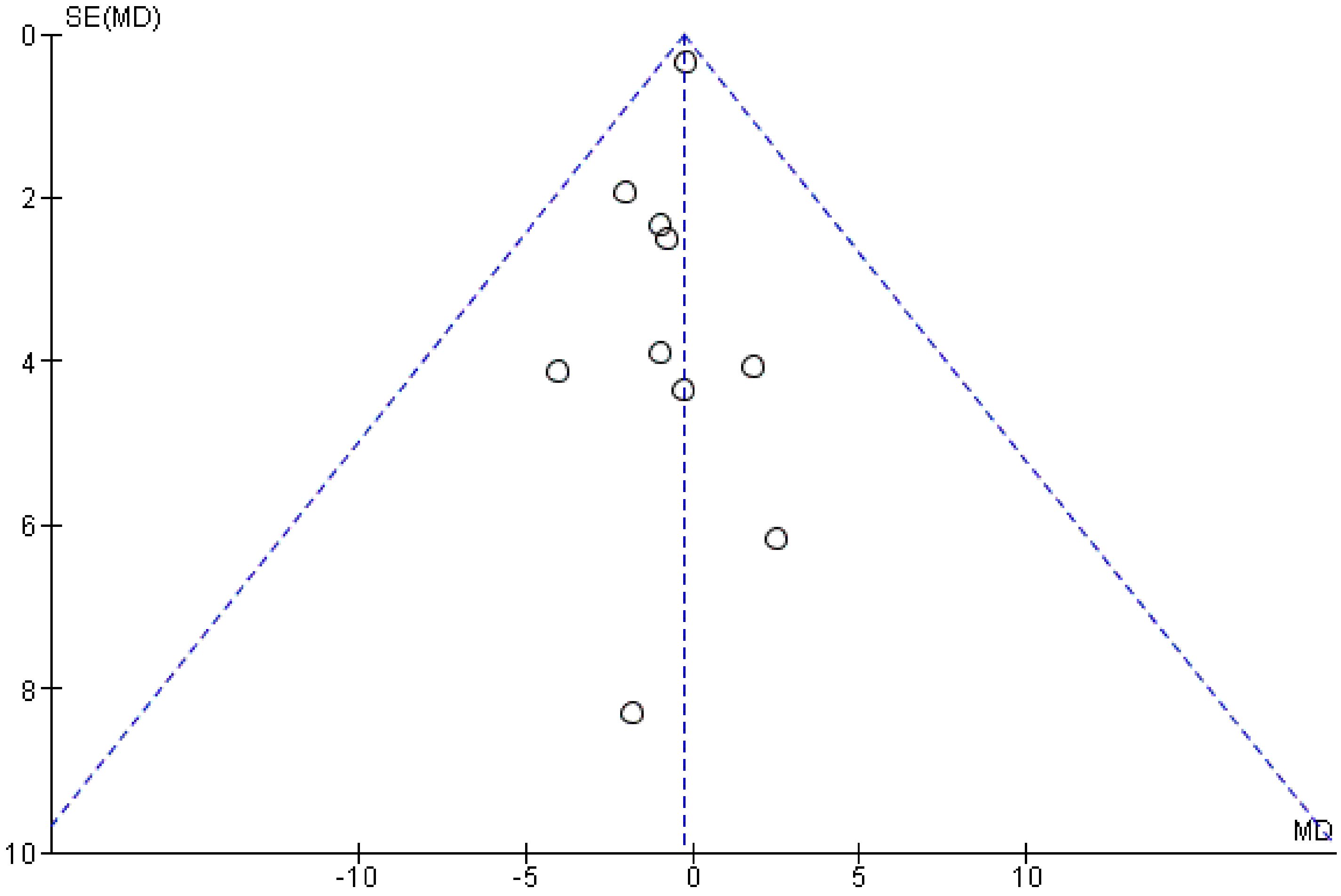

Supplementary Figure 26. Mean Glucose publication bias Funnel plot

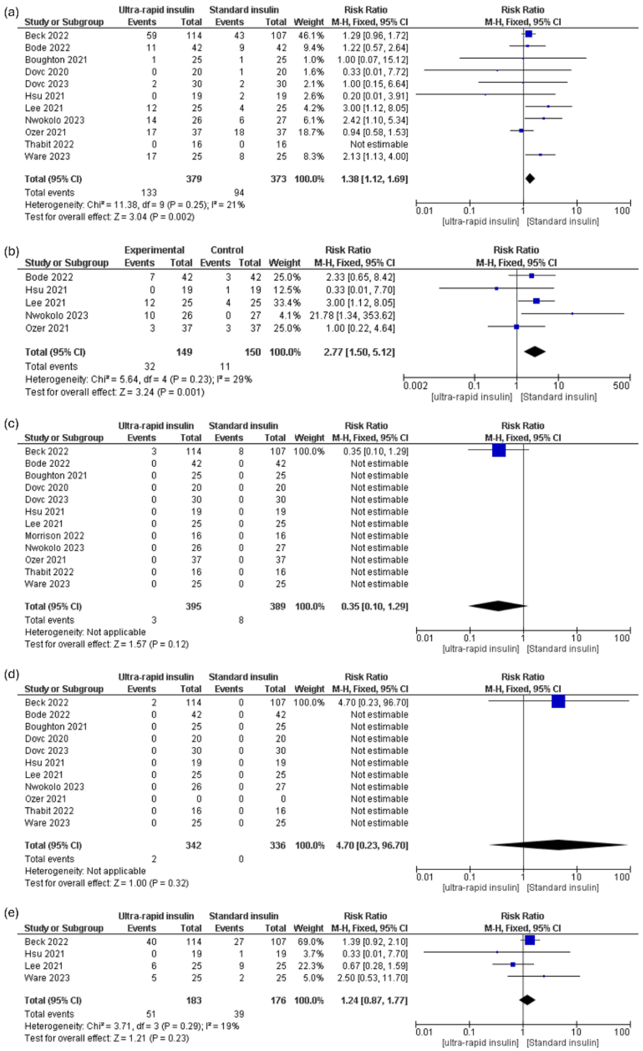

Figure 11. Forest plots of Safety outcomes: (a) Total adverse events, (b) Infusion set reaction, (c) Hypoglycemic events, (d) Diabetic ketoacidosis (DKA) events, (e) Events related to study device

Supplementary Figure 27. Forest plots of Safety outcomes: (a) Total adverse events, (b) Infusion set reaction, (c) Hypoglycemic events, (d) Diabetic ketoacidosis (DKA) events, (e) Events related to study device

(a)

Study or Subgroup	Ultra-rapid insulin Mean	SD	Total	Standard insulin Mean	SD	Total	Weight	Mean Difference IV, Fixed, 95% CI
Haliloglu 2024 Fisap	133	128	25	165	96	24	36.8%	-32.00 [-95.19, 31.19]
Haliloglu 2024 URLi	99	78	24	128	90	23	63.2%	-29.00 [-77.24, 19.24]
**Total (95% CI)**			**49**			**47**	**100.0%**	**-30.10 [-68.45, 8.24]**

Heterogeneity: Chi² = 0.01, df = 1 (P = 0.94); I² = 0%
Test for overall effect: Z = 1.54 (P = 0.12)

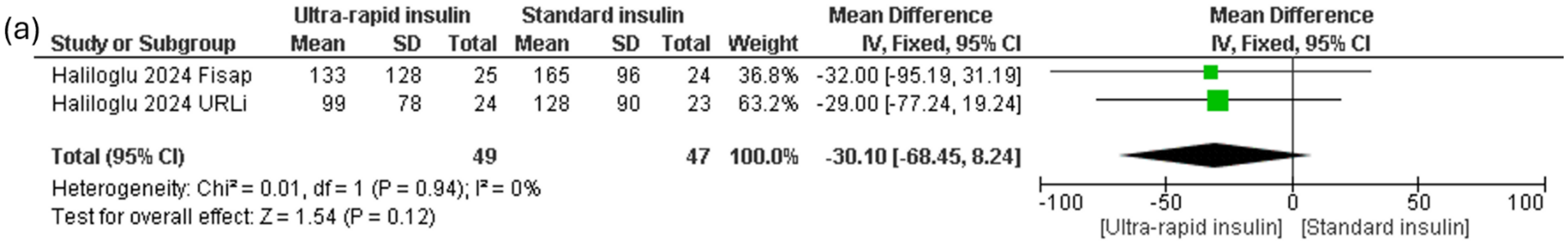

(b)

Study or Subgroup	Ultra-rapid insulin Mean	SD	Total	Standard insulin Mean	SD	Total	Weight	Mean Difference IV, Fixed, 95% CI
Haliloglu 2024 Fisap	193	141	22	197	128	21	40.5%	-4.00 [-84.43, 76.43]
Haliloglu 2024 URLi	103	98	22	197	122	21	59.5%	-94.00 [-160.33, -27.67]
**Total (95% CI)**			**44**			**42**	**100.0%**	**-57.57 [-108.74, -6.39]**

Heterogeneity: Chi² = 2.86, df = 1 (P = 0.09); I² = 65%
Test for overall effect: Z = 2.20 (P = 0.03)

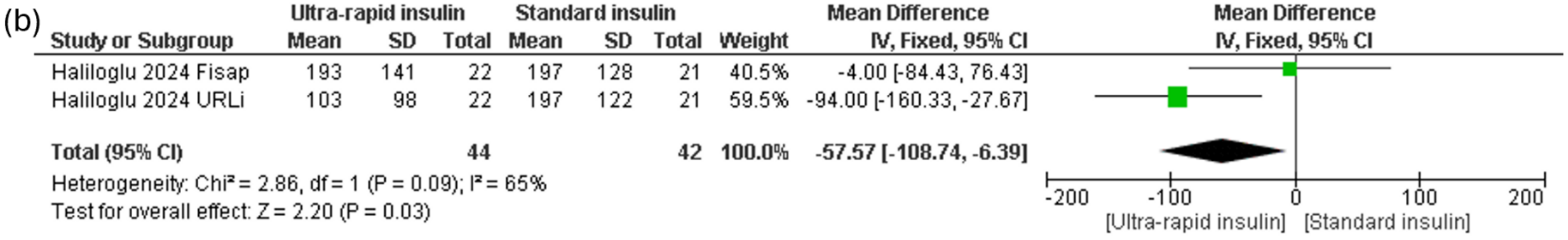

(c)

Study or Subgroup	Ultra-rapid insulin Mean	SD	Total	Standard insulin Mean	SD	Total	Weight	Mean Difference IV, Fixed, 95% CI
Haliloglu 2024 Fisap	96	135	25	100	115	25	35.4%	-4.00 [-73.52, 65.52]
Haliloglu 2024 URLi	38	91	24	87	91	24	64.6%	-49.00 [-100.49, 2.49]
**Total (95% CI)**			**49**			**49**	**100.0%**	**-33.06 [-74.43, 8.32]**

Heterogeneity: Chi² = 1.04, df = 1 (P = 0.31); I² = 4%
Test for overall effect: Z = 1.57 (P = 0.12)

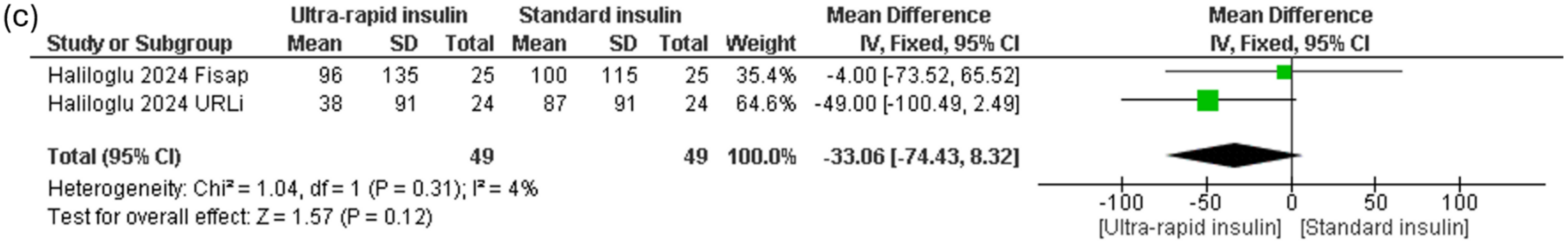

Supplementary Figure 28. Forest plots of Post meal glycemic control: (a) Glucose incremental area under the curve (iAUC) after 2 hour of launch, (b) Glucose iAUC after 2 hours of breakfast, (c) Glucose iAUC after 2 hours of dinner

(a)

Study or Subgroup	Ultra-rapid insulin Mean	SD	Total	Standard insulin Mean	SD	Total	Weight	Mean Difference IV, Fixed, 95% CI
Haliloglu 2024 Fisap	317	241	25	293	226	25	43.4%	24.00 [-105.51, 153.51]
Haliloglu 2024 URLi	211	183	24	298	224	24	54.3%	-87.00 [-202.72, 28.72]
Thabit 2022	190.9	1,017	16	458.1	539.8	16	2.3%	-267.20 [-831.37, 296.97]
**Total (95% CI)**			**65**			**65**	**100.0%**	**-42.97 [-128.27, 42.33]**

Heterogeneity: Chi² = 2.19, df = 2 (P = 0.33); I² = 9%
Test for overall effect: Z = 0.99 (P = 0.32)

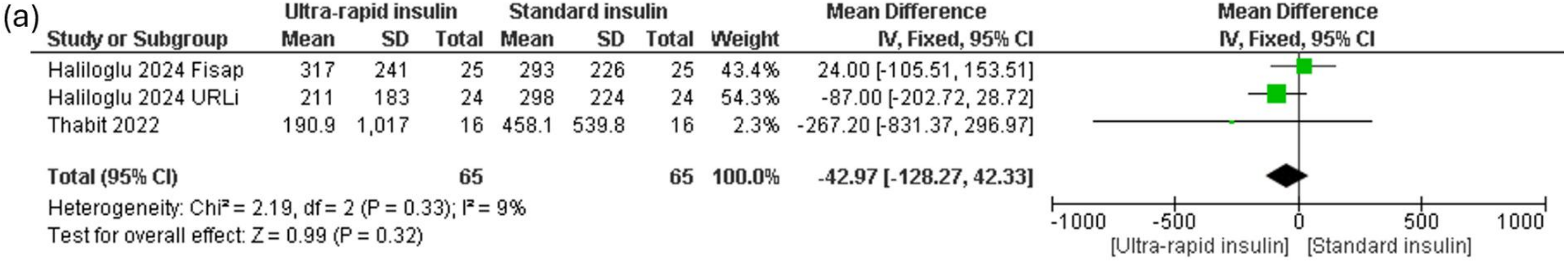

(b)

Study or Subgroup	Ultra-rapid insulin Mean	SD	Total	Standard insulin Mean	SD	Total	Weight	Mean Difference IV, Fixed, 95% CI
Haliloglu 2024 Fisap	367	197	22	343	201	21	39.5%	24.00 [-95.02, 143.02]
Haliloglu 2024 URLi	283	148	22	312	193	21	52.6%	-29.00 [-132.14, 74.14]
Thabit 2022	448.2	428.4	16	492.1	328.8	16	8.0%	-43.90 [-308.51, 220.71]
**Total (95% CI)**			**60**			**58**	**100.0%**	**-9.27 [-84.05, 65.50]**

Heterogeneity: Chi² = 0.51, df = 2 (P = 0.78); I² = 0%
Test for overall effect: Z = 0.24 (P = 0.81)

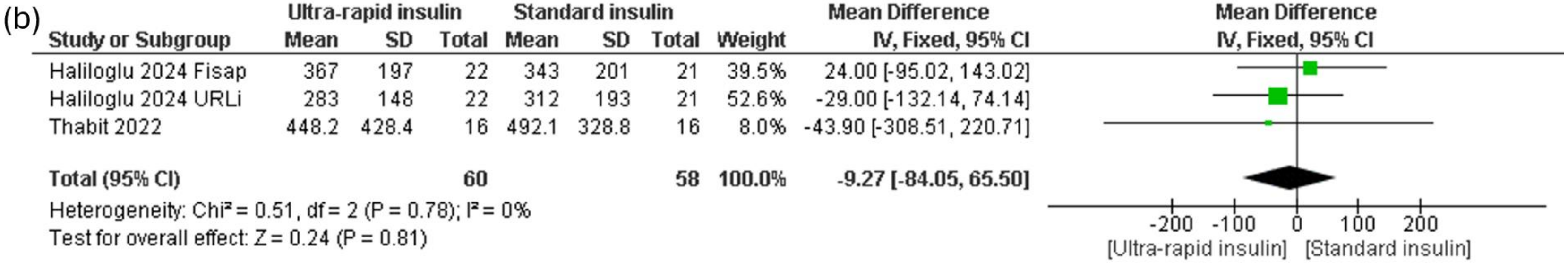

(c)

Study or Subgroup	Ultra-rapid insulin Mean	SD	Total	Standard insulin Mean	SD	Total	Weight	Mean Difference IV, Fixed, 95% CI
Haliloglu 2024 Fisap	317	241	25	293	226	25	43.4%	24.00 [-105.51, 153.51]
Haliloglu 2024 URLi	211	183	24	298	224	24	54.3%	-87.00 [-202.72, 28.72]
Thabit 2022	190.9	1,017	16	458.1	539.8	16	2.3%	-267.20 [-831.37, 296.97]
**Total (95% CI)**			**65**			**65**	**100.0%**	**-42.97 [-128.27, 42.33]**

Heterogeneity: Chi² = 2.19, df = 2 (P = 0.33); I² = 9%
Test for overall effect: Z = 0.99 (P = 0.32)

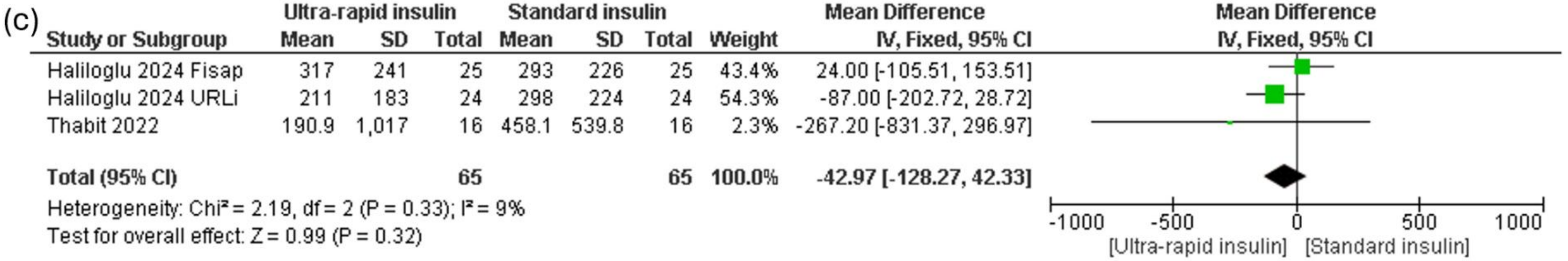

Supplementary Figure 29. Forest plots of Post meal glycemic control: (a) Glucose iAUC after 4 hours of lunch, (b) Glucose iAUC after 4 hours of breakfast, (c) Glucose iAUC after 4 hours of dinner

(a)

Study or Subgroup	Ultra-rapid insulin Mean	SD	Total	Standard insulin Mean	SD	Total	Weight	Mean Difference IV, Fixed, 95% CI
Haliloglu 2024 Fisap	72.1	11.4	25	71.2	8.1	24	50.0%	0.90 [-4.62, 6.42]
Haliloglu 2024 URLi	76.1	9.4	24	75.2	9.9	23	50.0%	0.90 [-4.62, 6.42]
**Total (95% CI)**			**49**			**47**	**100.0%**	**0.90 [-3.00, 4.80]**
Heterogeneity: Chi² = 0.00, df = 1 (P = 1.00); I² = 0%								
Test for overall effect: Z = 0.45 (P = 0.65)								

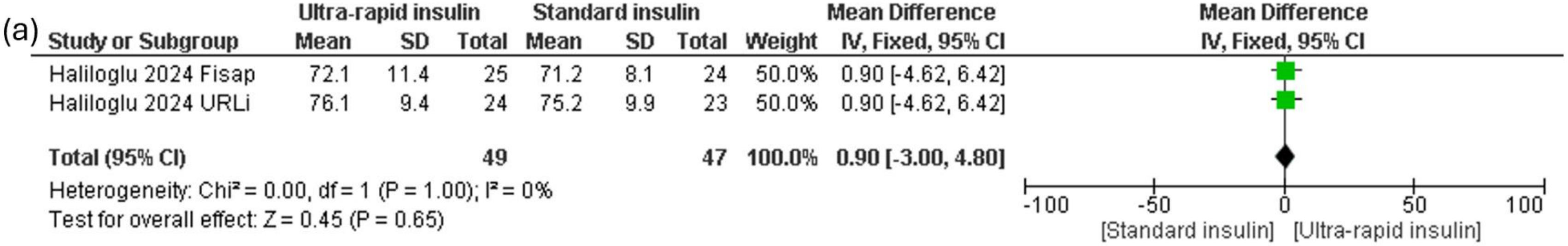

(b)

Study or Subgroup	Ultra-rapid insulin Mean	SD	Total	Standard insulin Mean	SD	Total	Weight	Mean Difference IV, Fixed, 95% CI
Haliloglu 2024 Fisap	76	13.1	22	75.5	10.9	21	41.7%	0.50 [-6.69, 7.69]
Haliloglu 2024 URLi	83.6	8.7	22	77.2	11.4	21	58.3%	6.40 [0.32, 12.48]
**Total (95% CI)**			**44**			**42**	**100.0%**	**3.94 [-0.70, 8.58]**
Heterogeneity: Chi² = 1.51, df = 1 (P = 0.22); I² = 34%								
Test for overall effect: Z = 1.66 (P = 0.10)								

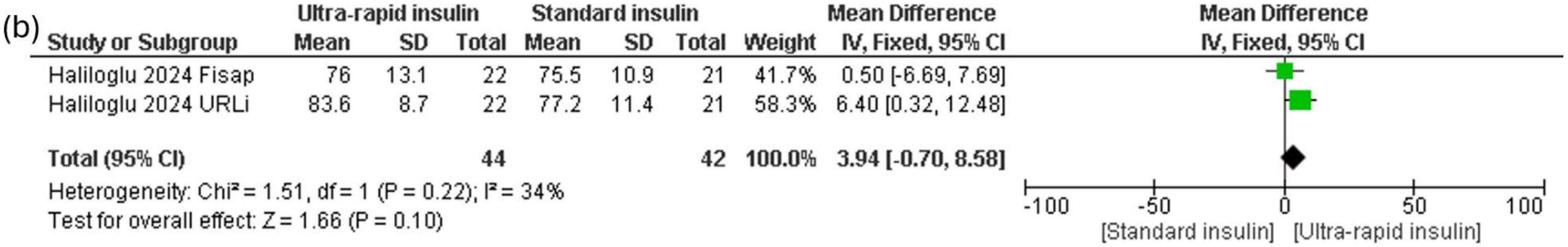

(c)

Study or Subgroup	Ultra-rapid insulin Mean	SD	Total	Standard insulin Mean	SD	Total	Weight	Mean Difference IV, Fixed, 95% CI
Haliloglu 2024 Fisap	72.2	10	25	74.5	10.7	25	53.5%	-2.30 [-8.04, 3.44]
Haliloglu 2024 URLi	79.1	9	24	73.4	12.5	24	46.5%	5.70 [-0.46, 11.86]
**Total (95% CI)**			**49**			**49**	**100.0%**	**1.42 [-2.78, 5.62]**
Heterogeneity: Chi² = 3.47, df = 1 (P = 0.06); I² = 71%								
Test for overall effect: Z = 0.66 (P = 0.51)								

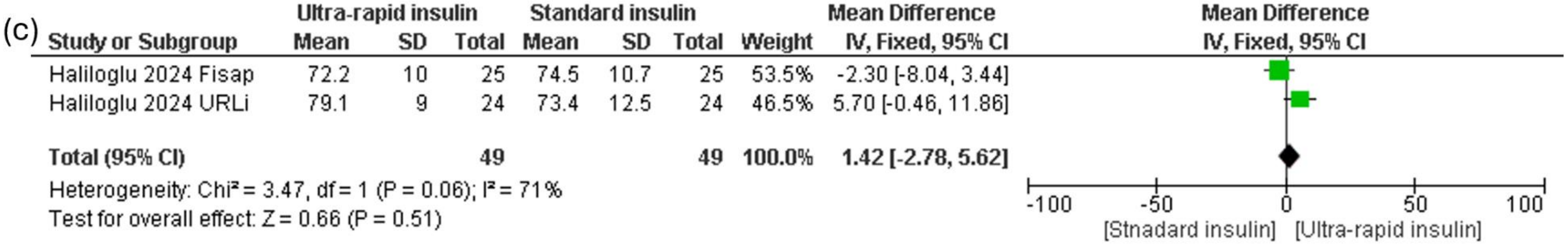

Supplementary Figure 30. Forest plots of Post meal glycemic control: (a) Lunch time in range (TIR) 70-180 mg/dl, (b) Breakfast time in range (TIR) 70-180 mg/dl, (c) Dinner time in range (TIR) 70-180 mg/dl

(a)

Study or Subgroup	Ultra-rapid insulin Mean	SD	Total	Standard insulin Mean	SD	Total	Weight	Mean Difference IV, Fixed, 95% CI
Haliloglu 2024 Fisap	2.87	3.3	25	2.17	2.05	24	43.8%	0.70 [-0.83, 2.23]
Haliloglu 2024 URLi	2.63	2.44	24	2.2	2.29	23	56.2%	0.43 [-0.92, 1.78]
**Total (95% CI)**			**49**			**47**	**100.0%**	**0.55 [-0.47, 1.56]**

Heterogeneity: Chi² = 0.07, df = 1 (P = 0.80); I² = 0%
Test for overall effect: Z = 1.06 (P = 0.29)

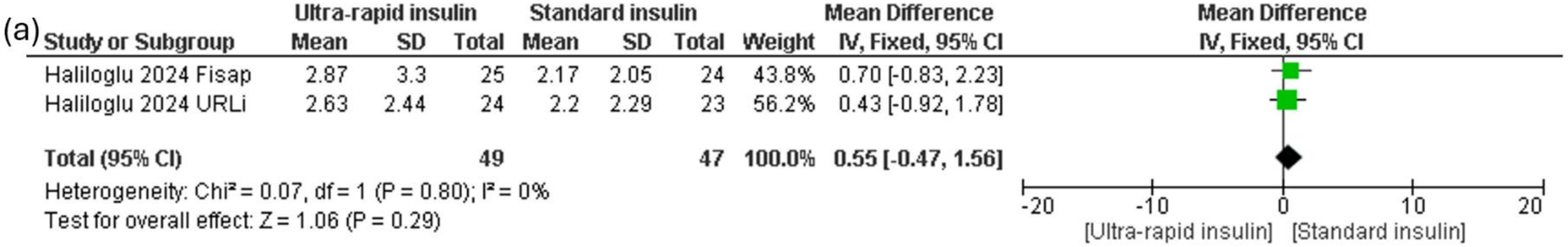

(b)

Study or Subgroup	Ultra-rapid insulin Mean	SD	Total	Standard insulin Mean	SD	Total	Weight	Mean Difference IV, Fixed, 95% CI
Haliloglu 2024 Fisap	2.5	2.77	22	3.27	3.02	21	24.1%	-0.77 [-2.50, 0.96]
Haliloglu 2024 URLi	2.67	2.22	22	1.97	0.72	21	75.9%	0.70 [-0.28, 1.68]
**Total (95% CI)**			**44**			**42**	**100.0%**	**0.35 [-0.51, 1.20]**

Heterogeneity: Chi² = 2.09, df = 1 (P = 0.15); I² = 52%
Test for overall effect: Z = 0.80 (P = 0.43)

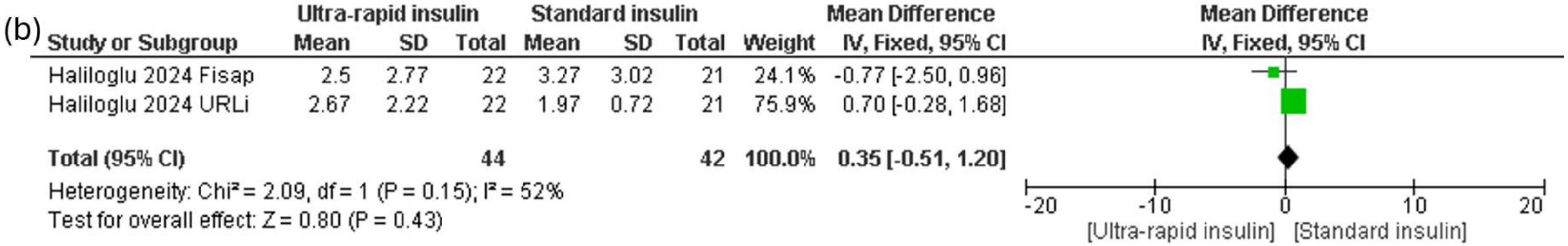

(c)

Study or Subgroup	Ultra-rapid insulin Mean	SD	Total	Standard insulin Mean	SD	Total	Weight	Mean Difference IV, Fixed, 95% CI
Haliloglu 2024 Fisap	3	3.62	25	3.53	3.46	25	29.0%	-0.53 [-2.49, 1.43]
Haliloglu 2024 URLi	3.23	1.97	24	2.53	2.44	24	71.0%	0.70 [-0.55, 1.95]
**Total (95% CI)**			**49**			**49**	**100.0%**	**0.34 [-0.71, 1.40]**

Heterogeneity: Chi² = 1.07, df = 1 (P = 0.30); I² = 7%
Test for overall effect: Z = 0.64 (P = 0.52)

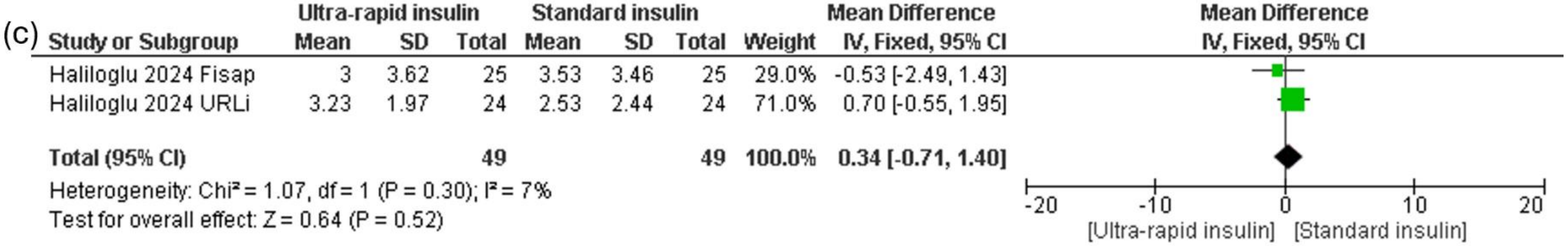

Supplementary Figure 31. Forest plots of Post meal glycemic control: (a) lunch TBR 70 mg/dl, (b) breakfast TBR 70 mg/dl, (c) dinner TBR 70 mg/dl

(a)

Study or Subgroup	Ultra-rapid insulin Mean	SD	Total	Standard insulin Mean	SD	Total	Weight	Mean Difference IV, Fixed, 95% CI
Haliloglu 2024 Fisap	24.7	13	25	26.1	8.9	24	47.5%	-1.40 [-7.62, 4.82]
Haliloglu 2024 URLi	20.9	9.7	24	22.2	10.9	23	52.5%	-1.30 [-7.21, 4.61]
**Total (95% CI)**			**49**			**47**	**100.0%**	**-1.35 [-5.63, 2.94]**

Heterogeneity: Chi² = 0.00, df = 1 (P = 0.98); I² = 0%
Test for overall effect: Z = 0.62 (P = 0.54)

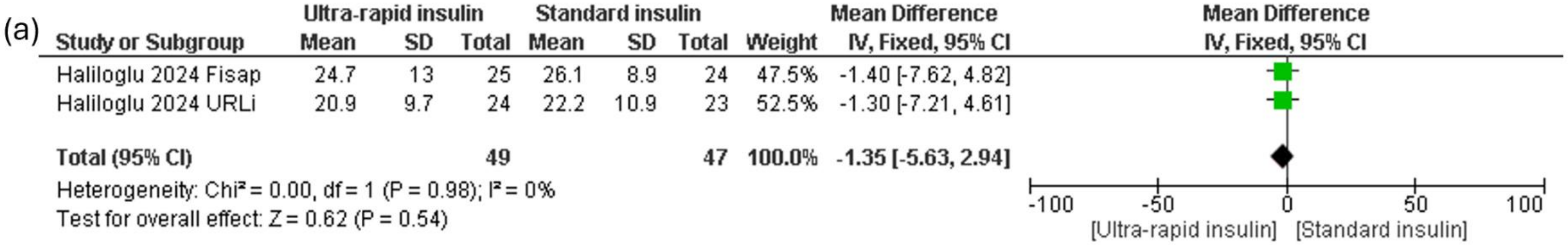

(b)

Study or Subgroup	Ultra-rapid insulin Mean	SD	Total	Standard insulin Mean	SD	Total	Weight	Mean Difference IV, Random, 95% CI
Haliloglu 2024 Fisap	21.8	13.9	22	20.9	11.5	21	46.0%	0.90 [-6.71, 8.51]
Haliloglu 2024 URLi	13.3	8.7	22	20.5	11.6	21	54.0%	-7.20 [-13.35, -1.05]
**Total (95% CI)**			**44**			**42**	**100.0%**	**-3.47 [-11.39, 4.44]**

Heterogeneity: Tau² = 20.34; Chi² = 2.63, df = 1 (P = 0.10); I² = 62%
Test for overall effect: Z = 0.86 (P = 0.39)

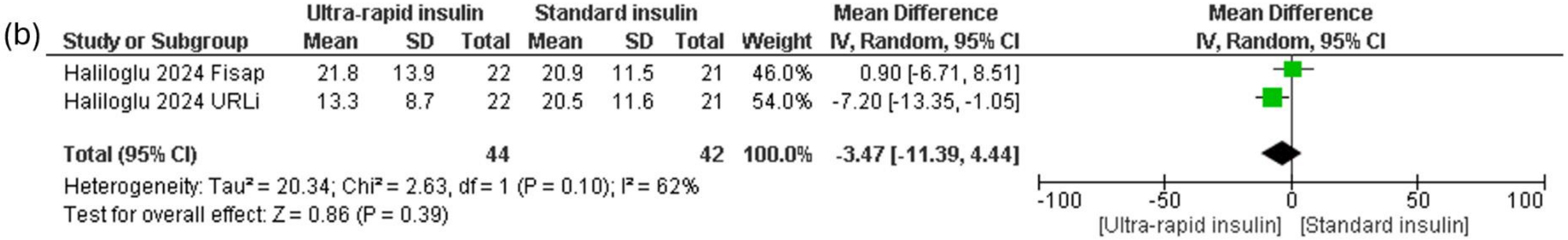

(c)

Study or Subgroup	Ultra-rapid insulin Mean	SD	Total	Standard insulin Mean	SD	Total	Weight	Mean Difference IV, Random, 95% CI
Haliloglu 2024 Fisap	24.4	11.8	25	21.7	11.6	25	50.5%	2.70 [-3.79, 9.19]
Haliloglu 2024 URLi	18	10	24	23.6	13.4	24	49.5%	-5.60 [-12.29, 1.09]
**Total (95% CI)**			**49**			**49**	**100.0%**	**-1.41 [-9.54, 6.73]**

Heterogeneity: Tau² = 23.14; Chi² = 3.05, df = 1 (P = 0.08); I² = 67%
Test for overall effect: Z = 0.34 (P = 0.73)

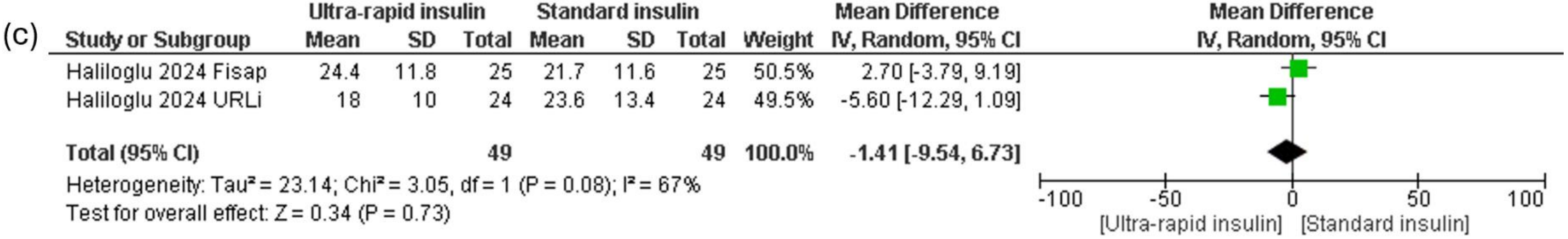

Supplementary Figure 32. Forest plots of Post meal glycemic control: (a) lunch TAR 180 mg/dl, (b) breakfast TAR 180 mg/dl, (c) dinner TAR 180 mg/dl

(a)

Study or Subgroup	ultra-rapid insulin Mean	SD	Total	Standard insulin Mean	SD	Total	Weight	Mean Difference IV, Fixed, 95% CI
Dovc 2020	80.57	29.92	20	78.47	38.22	20	6.9%	2.10 [-19.17, 23.37]
Dovc 2023	78	10	12	81	10	12	49.1%	-3.00 [-11.00, 5.00]
Morrison 2022	93.06	16.93	16	98.61	3.39	16	43.9%	-5.55 [-14.01, 2.91]
**Total (95% CI)**			**48**			**48**	**100.0%**	**-3.77 [-9.37, 1.84]**

Heterogeneity: Chi² = 0.50, df = 2 (P = 0.78); I² = 0%
Test for overall effect: Z = 1.32 (P = 0.19)

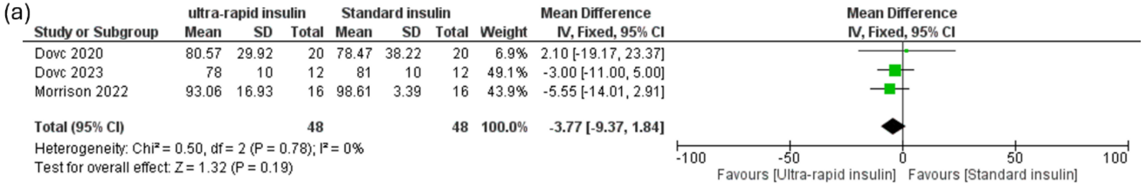

(b)

Study or Subgroup	Ultra-rapid insulin Mean	SD	Total	Standard insulin Mean	SD	Total	Weight	Mean Difference IV, Fixed, 95% CI
Dovc 2020	141.1	33.19	20	146.17	25.69	20	0.3%	-5.07 [-23.46, 13.32]
Dovc 2023	128	21	12	129	14	12	0.5%	-1.00 [-15.28, 13.28]
Haliloglu 2024 Fisap	3	3.62	25	3.53	3.46	25	28.6%	-0.53 [-2.49, 1.43]
Haliloglu 2024 URLi	3.23	1.97	24	2.53	2.44	24	70.1%	0.70 [-0.55, 1.95]
Morrison 2022	126.126	24.144	16	120.36	21.982	16	0.4%	5.77 [-10.23, 21.77]
**Total (95% CI)**			**97**			**97**	**100.0%**	**0.34 [-0.71, 1.39]**

Heterogeneity: Chi² = 1.88, df = 4 (P = 0.76); I² = 0%
Test for overall effect: Z = 0.64 (P = 0.52)

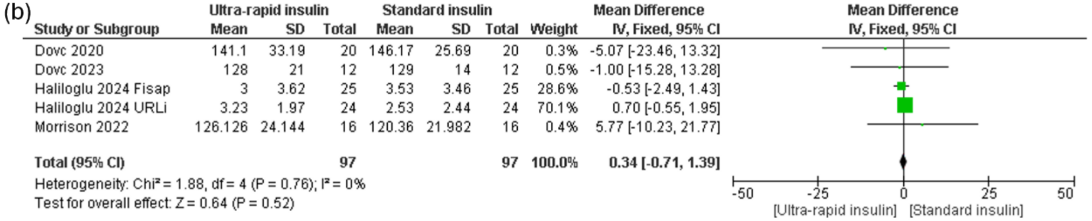

(c)

Study or Subgroup	Ultra-rapid insulin Mean	SD	Total	Standard insulin Mean	SD	Total	Weight	Mean Difference IV, Fixed, 95% CI
Dovc 2020	0	0	20	0	0	20		Not estimable
Dovc 2023	6.8	7.72	12	5	5.28	12	3.8%	1.80 [-3.49, 7.09]
Haliloglu 2024 Fisap	3	3.62	25	3.53	3.46	25	27.9%	-0.53 [-2.49, 1.43]
Haliloglu 2024 URLi	3.23	1.97	24	2.53	2.44	24	68.3%	0.70 [-0.55, 1.95]
Morrison 2022	0	0	16	0	0	16		Not estimable
**Total (95% CI)**			**97**			**97**	**100.0%**	**0.40 [-0.64, 1.44]**

Heterogeneity: Chi² = 1.35, df = 2 (P = 0.51); I² = 0%
Test for overall effect: Z = 0.75 (P = 0.45)

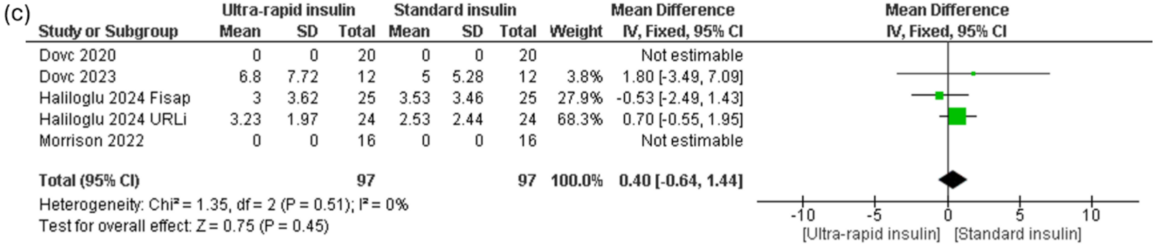

(d)

Study or Subgroup	Ultra-rapid insulin Mean	SD	Total	Standard insulin Mean	SD	Total	Weight	Mean Difference IV, Fixed, 95% CI
Dovc 2023	14	13	12	14	9	12	100.0%	0.00 [-8.95, 8.95]
Morrison 2022	1.39	3.39	16	0	0	16		Not estimable
**Total (95% CI)**			**28**			**28**	**100.0%**	**0.00 [-8.95, 8.95]**

Heterogeneity: Not applicable
Test for overall effect: Z = 0.00 (P = 1.00)

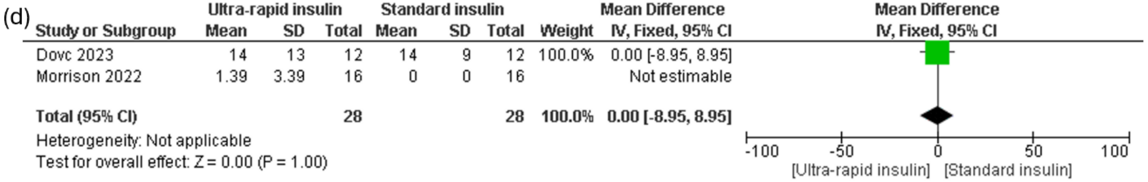

(e)

Study or Subgroup	Ultra-rapid insulin Mean	SD	Total	Standard insulin Mean	SD	Total	Weight	Mean Difference IV, Fixed, 95% CI
Dovc 2020	0	0	20	1.73	4.15	20		Not estimable
Dovc 2023	0.13	0.34	12	2.13	4.11	12	100.0%	-2.00 [-4.33, 0.33]
Morrison 2022	0	0	16	0	0	16		Not estimable
**Total (95% CI)**			**48**			**48**	**100.0%**	**-2.00 [-4.33, 0.33]**

Heterogeneity: Not applicable
Test for overall effect: Z = 1.68 (P = 0.09)

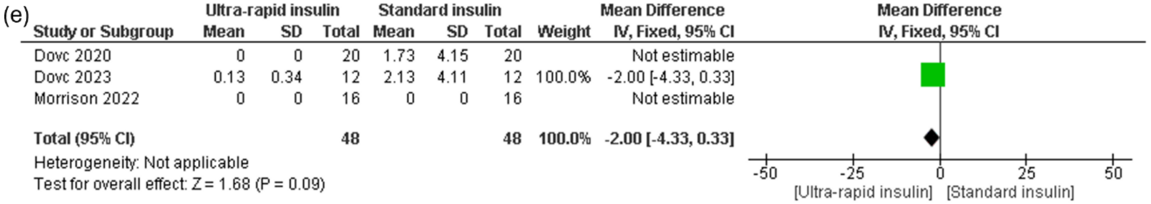

Supplementary Figure 33. Forest plots of Post exercise glycemic control: (a) TIR 70-180 mg/dl after 2 hours of exercise, (b) mean glucose after 2 hours of exercise, (c) TBR 70 mg/dl after 2 hours of exercise, (d) TAR 180 mg/dl, (e) TAR 250 mg/dl

	ultra-rapid insulin			Standard insulin				Mean Difference
**Study or Subgroup**	**Mean**	**SD**	**Total**	**Mean**	**SD**	**Total**	**Weight**	**IV, Random, 95% CI**
Dovc 2020	80.57	29.92	20	78.47	38.22	20	12.4%	2.10 [-19.17, 23.37]
Dovc 2023	78	10	12	81	10	12	87.6%	-3.00 [-11.00, 5.00]
Morrison 2022	93.06	16.93	16	0	3.39	16	0.0%	93.06 [84.60, 101.52]
**Total (95% CI)**			**32**			**32**	**100.0%**	**-2.37 [-9.86, 5.12]**

Heterogeneity: Tau² = 0.00; Chi² = 0.19, df = 1 (P = 0.66); I² = 0%
Test for overall effect: Z = 0.62 (P = 0.54)

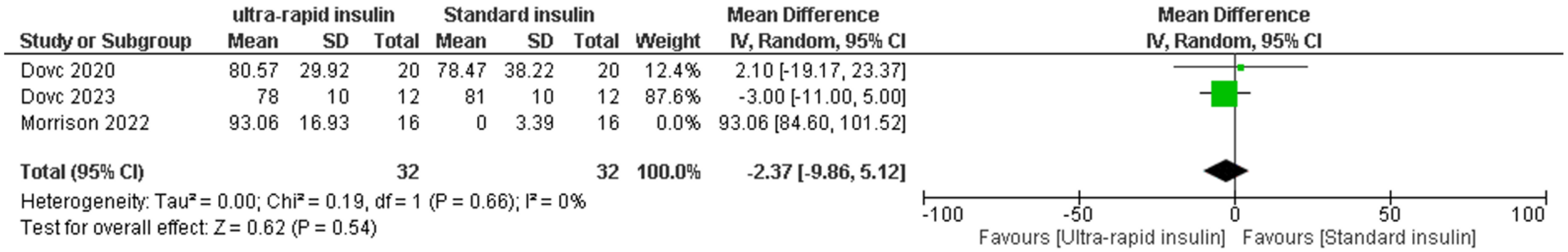

Supplementary Figure 34. Time in Range 70-180 after 2 hours of exercise sensitivity analysis

(a)

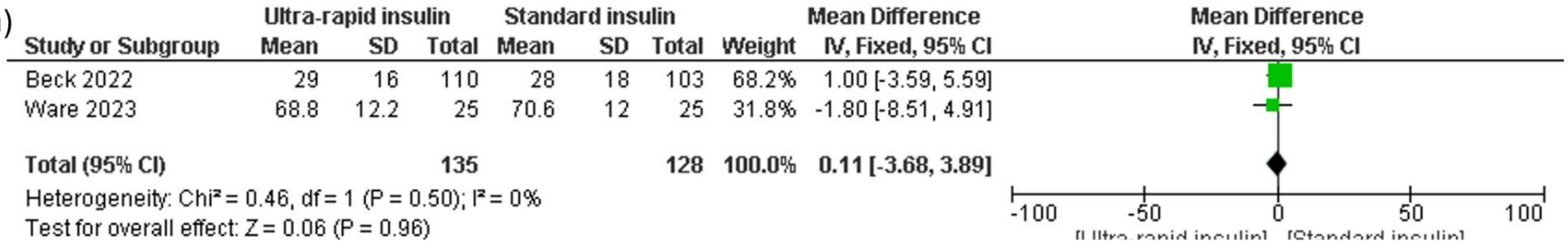

(b)

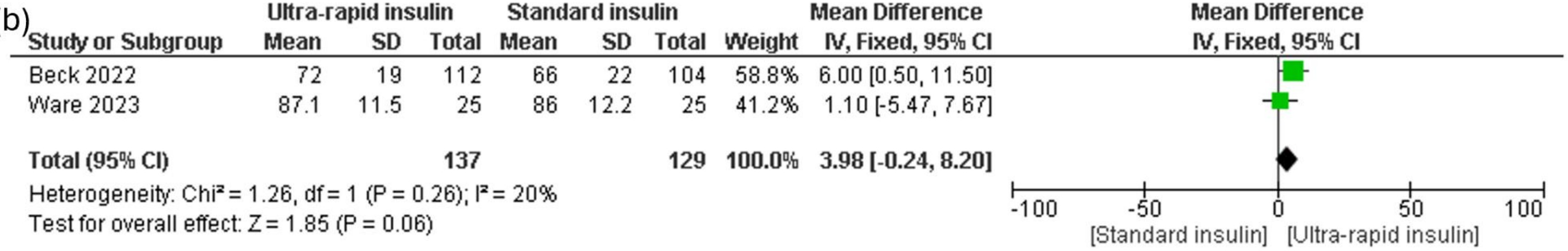

Supplementary Figure 35. Forest plots of psychological outcomes: (a) hypoglycemia fear scale, (b) Insulin delivery Systems: Perspectives, Ideas, Reflections and Expectations score [INSPIRE score]

Study or Subgroup	Ultra-rapid insulin Mean	SD	Total	Standard insulin Mean	SD	Total	Weight	Mean Difference IV, Fixed, 95% CI
**10.1.1 CamAPS FX**								
Boughton 2021	75	8	25	75	8	25	6.0%	0.00 [-4.43, 4.43]
Nwokolo 2023	78.7	9.8	26	76.2	9.6	27	4.3%	2.50 [-2.73, 7.73]
Thabit 2022	61.8	14.3	16	61.5	12.8	16	1.3%	0.30 [-9.10, 9.70]
Ware 2023	64.2	8.8	25	64.6	8.8	25	4.9%	-0.40 [-5.28, 4.48]
**Subtotal (95% CI)**			**92**			**93**	**16.5%**	**0.56 [-2.11, 3.22]**
Heterogeneity: Chi² = 0.74, df = 3 (P = 0.86); I² = 0%								
Test for overall effect: Z = 0.41 (P = 0.68)								
**10.1.2 Medtronic 670G**								
Bode 2022	77	5.8	42	77.8	5.8	42	19.1%	-0.80 [-3.28, 1.68]
Hsu 2021	78.4	9.3	19	75.3	9.5	19	3.3%	3.10 [-2.88, 9.08]
**Subtotal (95% CI)**			**61**			**61**	**22.4%**	**-0.23 [-2.52, 2.06]**
Heterogeneity: Chi² = 1.39, df = 1 (P = 0.24); I² = 28%								
Test for overall effect: Z = 0.19 (P = 0.85)								
**10.1.3 Medtronic 780G**								
Dovc 2023	78	6	30	77	7	30	10.8%	1.00 [-2.30, 4.30]
Morrison 2022	85.81	10.86	16	81.59	17.65	16	1.1%	4.22 [-5.93, 14.37]
**Subtotal (95% CI)**			**46**			**46**	**11.9%**	**1.31 [-1.83, 4.45]**
Heterogeneity: Chi² = 0.35, df = 1 (P = 0.55); I² = 0%								
Test for overall effect: Z = 0.82 (P = 0.41)								
**10.1.4 iLet bionic pancreas**								
Beck 2022	71	8	114	69	8	107	26.4%	2.00 [-0.11, 4.11]
**Subtotal (95% CI)**			**114**			**107**	**26.4%**	**2.00 [-0.11, 4.11]**
Heterogeneity: Not applicable								
Test for overall effect: Z = 1.86 (P = 0.06)								
**10.1.5 Others**								
Dovc 2020	54.83	8.06	20	57.6	8.3	20	4.6%	-2.77 [-7.84, 2.30]
Lee 2021	81.5	4.09	25	80	5.03	25	18.2%	1.50 [-1.04, 4.04]
**Subtotal (95% CI)**			**45**			**45**	**22.8%**	**0.64 [-1.63, 2.91]**
Heterogeneity: Chi² = 2.18, df = 1 (P = 0.14); I² = 54%								
Test for overall effect: Z = 0.55 (P = 0.58)								
**Total (95% CI)**			**358**			**352**	**100.0%**	**0.87 [-0.21, 1.95]**
Heterogeneity: Chi² = 6.81, df = 10 (P = 0.74); I² = 0%								
Test for overall effect: Z = 1.57 (P = 0.12)								
Test for subgroup differences: Chi² = 2.15, df = 4 (P = 0.71), I² = 0%								

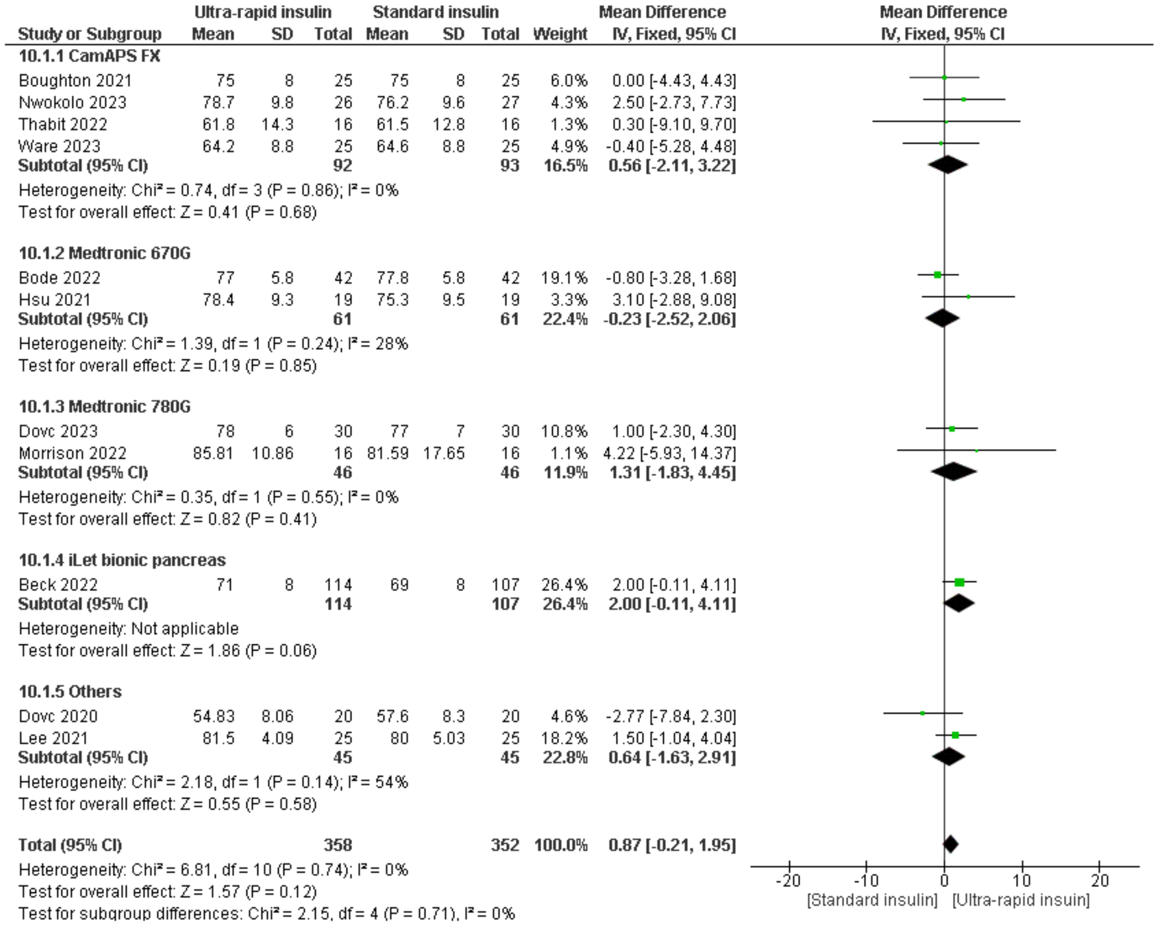

Supplementary Figure 36. TIR 70-180 mg/dl subgroup analysis by device type

Study or Subgroup	Ultra-rapid insulin Mean	SD	Total	Standard insulin Mean	SD	Total	Weight	Mean Difference IV, Fixed, 95% CI
**11.1.1 Fisap**								
Beck 2022	71	8	114	69	8	107	26.4%	2.00 [-0.11, 4.11]
Boughton 2021	75	8	25	75	8	25	6.0%	0.00 [-4.43, 4.43]
Dovc 2020	54.83	8.06	20	57.6	8.3	20	4.6%	-2.77 [-7.84, 2.30]
Dovc 2023	78	6	30	77	7	30	10.8%	1.00 [-2.30, 4.30]
Hsu 2021	78.4	9.3	19	75.3	9.5	19	3.3%	3.10 [-2.88, 9.08]
Lee 2021	81.5	4.09	25	80	5.03	25	18.2%	1.50 [-1.04, 4.04]
Morrison 2022	85.81	10.86	16	81.59	17.65	16	1.1%	4.22 [-5.93, 14.37]
Thabit 2022	61.8	14.3	16	61.5	12.8	16	1.3%	0.30 [-9.10, 9.70]
Ware 2023	64.2	8.8	25	64.6	8.8	25	4.9%	-0.40 [-5.28, 4.48]
**Subtotal (95% CI)**			**290**			**283**	**76.6%**	**1.20 [-0.04, 2.43]**
Heterogeneity: Chi² = 4.43, df = 8 (P = 0.82); I² = 0%								
Test for overall effect: Z = 1.89 (P = 0.06)								
**11.1.2 URLi**								
Bode 2022	77	5.8	42	77.8	5.8	42	19.1%	-0.80 [-3.28, 1.68]
Nwokolo 2023	78.7	9.8	26	76.2	9.6	27	4.3%	2.50 [-2.73, 7.73]
**Subtotal (95% CI)**			**68**			**69**	**23.4%**	**-0.19 [-2.43, 2.05]**
Heterogeneity: Chi² = 1.25, df = 1 (P = 0.26); I² = 20%								
Test for overall effect: Z = 0.17 (P = 0.87)								
**Total (95% CI)**			**358**			**352**	**100.0%**	**0.87 [-0.21, 1.95]**
Heterogeneity: Chi² = 6.81, df = 10 (P = 0.74); I² = 0%								
Test for overall effect: Z = 1.57 (P = 0.12)								
Test for subgroup differences: Chi² = 1.13, df = 1 (P = 0.29), I² = 11.5%								

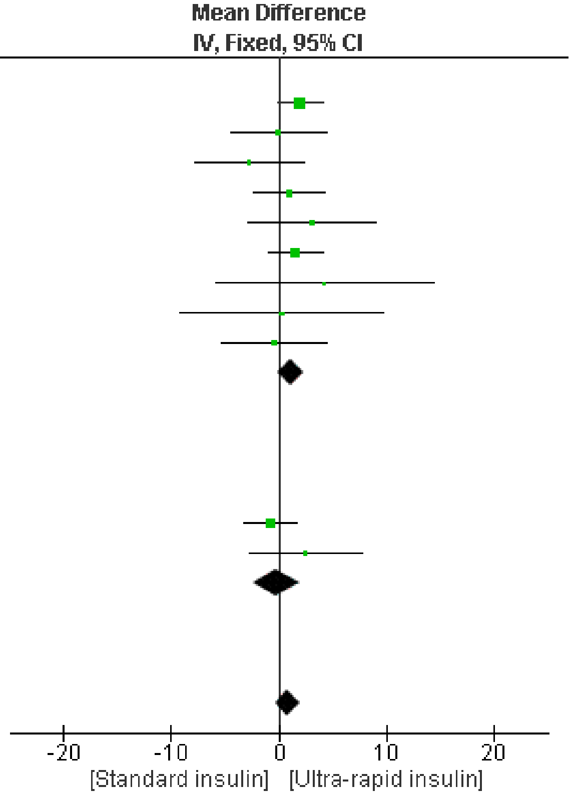

Supplementary Figure 37. TIR 70-180 mg/dl subgroup analysis by insulin type

Supplementary table 1 Search strategy

Database	Search Query
PubMed	("closed loop"[All Fields] OR "artificial pancreas"[All Fields] OR "automated insulin delivery"[All Fields]) AND ("insulin"[MeSH Terms] OR "insulin"[All Fields] OR "insulin s"[All Fields] OR "insuline"[All Fields] OR "insulinic"[All Fields] OR "insulinization"[All Fields] OR "insulinized"[All Fields] OR "insulins"[MeSH Terms] OR "insulins"[All Fields] OR "aspart"[All Fields] OR ("insulin lispro"[MeSH Terms] OR ("insulin"[All Fields] AND "lispro"[All Fields]) OR "insulin lispro"[All Fields] OR "lispro"[All Fields]) OR "URLi"[All Fields] OR "lyumjev"[All Fields] OR "FIASP"[All Fields]) AND ("ultrarapid*"[All Fields] OR "ultrashort*"[All Fields] OR "fast*"[All Fields] OR "rapid*"[All Fields])
Scopus	TITLE-ABS-KEY (("closed loop" OR "artificial pancreas" OR "automated insulin delivery") AND (insulin OR aspart OR lispro OR urli OR lyumjev OR fiasp) AND (ultrarapid* OR ultrashort* OR fast* OR rapid*))
Web of science	("closed loop" OR "artificial pancreas" OR "automated insulin delivery") AND (insulin OR aspart OR lispro OR URLi OR lyumjev OR FIASP) AND (ultrarapid* OR ultrashort* OR fast* OR rapid*) (Topic)
Cochrane	("closed loop" OR "artificial pancreas" OR "automated insulin delivery") AND (insulin OR aspart OR lispro OR URLi OR lyumjev OR FIASP) AND (ultrarapid* OR ultrashort* OR fast* OR rapid*) in All Text - (Word variations have been searched)

Supplementary table 2 meta- regression analysis

Outcomes	Covariate	Coefficient	Standard error	95% Confidence interval	95% Confidence interval	P value
**TIR**	Diabetes duration	0.04	0.069	-0.095	0.176	0.558
	Hba1c level	0.425	1.068	-0.592	2.52	0.69
**TBR**	Diabetes duration	-0.02	0.016	-0.052	0.012	0.22
	Hba1c level	0.106	0.2	-0.289	0.502	0.6
**TAR**	Diabetes duration	0.03	-0.104	0.165	0.069	0.66
	Hba1c level	-0.43	1.31	-2.99	2.14	0.74
**mean glucose**	Diabetes duration	0.025	0.086	-0.144	0.193	0.77
	Hba1c level	-0.69	1.67	-3.97	2.58	0.68

Supplementary table 3 subgroup analysis

Outcomes	*Comparisons, n*	*MD (95% CI)*	*p*	I^2%	*p diff*
TIR 70-180 mg/dl					
24-hour data subgroups					
Country					
USA	*3*	*0.98 [-0.57, 2.53]*	*0.22*	*40*	*0.98*
UK	*3*	*0.87 [-2.47, 4.20]*	*0.61*	*0*	
Others	*5*	*0.74 [-0.96, 2.44]*	*0.39*	*0*	
Age group					
Adults	*9*	*0.93 [-0.25, 2.11]*	*0.12*	*0*	*0.81*
Adolescents and children	*2*	*0.56 [-2.17, 3.29]*	*0.69*	*0*	
Device					
CamAPX FX	*4*	*0.56 [-2.11, 3.22]*	*0.68*	*0*	*0.71*
Medtronic 670G	*2*	*-0.23 [-2.52, 2.06]*	*0.85*	*28*	
Medtronic 780G	*2*	*1.31 [-1.83, 4.45]*	*0.41*	*0*	
Let bionic pancreas	*1*	*2.00 [-0.11, 4.11]*	*0.06*	*NA*	
Others	*2*	*0.64 [-1.63, 2.91]*	*0.58*	*54*	
Insulin					
Fisap	*9*	*1.20 [-0.04, 2.43]*	*0.06*	*0*	*0.29*
URLi	*2*	*-0.19 [-2.43, 2.05]*	*0.87*	*20*	
Study duratioin					
Long	*8*	*1.02 [-0.11, 2.14]*	*0.08*	*0*	*0.34*
short	*3*	*-1.06 [-5.14, 3.03]*	*0.61*	*0*	
Nighttime data subgroups					
Country					
USA	*2*	*-2.02 [-2.61, -1.43]*	*0.0001*	*43*	*0.11*
UK	*3*	*1.95 [-1.94, 5.83]*	*0.33*	*0*	
Others	*4*	*-0.83 [-4.01, 2.35]*	*0.61*	*0*	
Age group					
Adults	*7*	*-1.96 [-2.54, -1.38]*	*0.0001*	*19*	*0.19*
Adolescents and children	*2*	*0.53 [-3.18, 4.24]*	*0.78*	*0*	
Device					
CamAPX FX	*4*	*0.98 [-2.20, 4.17]*	*0.55*	*0*	*0.12*

Medtronic 670G	*1*	*-2.10 [-2.70, -1.50]*	*0.0001*	*NA*	
Medtronic 780G	*2*	*0.53 [-3.75, 4.80]*	*0.81*	*0*	
Let bionic pancreas	*1*	*0.00 [-3.05, 3.05]*	*1*	*NA*	
Others	*1*	*-6.47 [-15.55, 2.61]*		*NA*	
Insulin					
Fisap	*7*	*-0.34 [-2.39, 1.72]*	*0.75*	*0*	*0.12*
URLi	*2*	*-2.03 [-2.62, -1.43]*	*0.0001*	*77*	
Study duratioin					
Long	*6*	*-1.89 [-2.46, -1.31]*	*0.0001*	*0.64*	
short	*3*	*-3.39 [-9.68, 2.89]*	*0.29*		
Daytime data subgroups					
Country					
USA	*2*	*1.09 [-2.33, 4.51]*	*0.53*	*90*	*0.97*
UK	*2*	*0.75 [-2.85, 4.35]*	*0.68*	*0*	
Others	*2*	*1.29 [-1.36, 3.93]*	*0.34*	*0*	
Age group					
Adults	*4*	*0.98 [-1.35, 3.32]*	*0.41*	*72*	*0.88*
Adolescents and children	*2*	*1.26 [-1.46, 3.98]*	*0.36*	*0*	
Device					
CamAPX FX	*3*	*0.45 [-2.34, 3.25]*	*0.75*	*0*	*0.006*
Medtronic 670G	*1*	*-0.50 [-0.93, -0.07]*	*0.02*	*NA*	
Medtronic 780G	*1*	*2.00 [-1.30, 5.30]*	*0.23*	*NA*	
Let bionic pancreas	*1*	*3.00 [0.88, 5.12]*	*0.006*	*NA*	
Insulin					
Fisap	*4*	*2.05 [0.49, 3.62]*	*0.01*	*0*	*0.002*
URLi	*2*	*-0.48 [-0.91, -0.06]*	*0.03*	*0*	
TBR <70mg/dl					
24 hour data subgroups					
Country					
USA	*3*	*-0.37 [-0.63, -0.10]*	*0.008*	*21*	*0.87*
UK	*2*	*-0.05 [-1.23, 1.12]*	*0.93*	*0*	
Others	*5*	*ne0.32 [ne0.7, 0.05]*	*0.09*	*0*	
Age group					

Adults	*10*	*-0.34 [-0.56, -0.13]*	*0.002*	*0*	*0.82*
Adolescents and children	*2*	*-0.25 [-1.10, 0.60]*	*0.57*	*0*	
Device					
CamAPX FX	*3*	*-0.39 [-1.12, 0.35]*	*0.3*	*0*	*0.55*
Medtronic 670G	*2*	*-0.72 [-1.22, -0.21]*	*0.006*	*0*	
Medtronic 780G	*2*	*-0.48 [-1.26, 0.30]*	*0.23*	*0*	
Let bionic pancreas	*1*	*-0.23 [-0.55, 0.09]*	*0.15*	*NA*	
Others	*2*	*-0.19 [-0.68, 0.29]*	*0.44*	*0*	
Insulin					
Fisap	*9*	*-0.28 [-0.51, -0.04]*	*0.02*	*0*	*0.17*
URLi	*1*	*-0.70 [-1.25, -0.15]*	*0.01*	*0*	
Study duratioin					
Long	*7*	*-0.38 [-0.62, -0.14]*	*0.002*	*0*	*0.49*
short	*3*	*-0.19 [-0.67, 0.29]*	*0.44*	*0*	
Nighttime data subgroups					
Country					
USA	*7*	*0.13 [-0.43, 0.69]*	*0.65*	*0*	*0*
Others	*1*	*NA*	*NA*	*NA*	
Age group					
Adults	*6*	*0.19 [-0.49, 0.88]*	*0.58*	*92*	*0.66*
Adolescents and children	*2*	*-0.04 [-0.86, 0.77]*	*0.91*	*0*	
Device					
CamAPX FX	*3*	*0.67 [-0.86, 2.19]*	*0.39*	*90*	*0.6*
Medtronic 670G	*1*	*-0.30 [-0.47, -0.13]*	*0.0006*	*NA*	
Medtronic 780G	*2*	*-0.20 [-1.28, 0.88]*	*0.72*	*NA*	
Let bionic pancreas	*1*	*-0.40 [-0.75, -0.05]*	*0.02*	*NA*	
Others	*1*	*NA*	*NA*	*NA*	
Insulin					
Fisap	*6*	*0.40 [-0.88, 1.67]*	*0.54*	*91*	*0.29*
URLi	*2*	*-0.29 [-0.46, -0.13]*	*0.0005*	*0*	
Study duratioin					
Long	*6*	*0.13 [-0.43, 0.69]*	*0.65*	*0*	*NA*
short	*2*	*NA*	*NA*		

Daytime data subgroups					
Country					
USA	*2*	*-0.58 [-1.07, -0.10]*	*0.02*	*86*	*0.64*
UK	*2*	*-0.14 [-0.93, 0.65]*	*0.72*	*0*	
Others	*2*	*-0.52 [-1.32, 0.27]*	*0.19*	*0*	
Age group					
Adults	*4*	*-0.54 [-0.92, -0.15]*	*0.006*	*67*	*0.66*
Adolescents and children	*2*	*-0.31 [-1.24, 0.61]*	*0.51*	*0*	
Device					
CamAPX FX	*3*	*-0.30 [-0.97, 0.36]*	*0.37*	*0*	*0.02*
Medtronic 670G	*1*	*-0.80 [-0.89, -0.71]*	*0.0001*	*NA*	
Medtronic 780G	*1*	*-0.40 [-1.44, 0.64]*	*0.45*	*NA*	
Let bionic pancreas	*1*	*-0.30 [-0.66, 0.06]*	*0.1*	*NA*	
Insulin					
Fisap	*4*	*-0.33 [-0.65, -0.01]*	*0.05*	*0*	*0.35*
URLi	*2*	*-0.63 [-1.18, -0.09]*	*0.02*	*52*	
TAR >180 mg/dl					
24 hour data subgroups					
Country					
USA	*3*	*0.13 [-1.39, 1.65]*	*0.86*	*40*	*0.66*
UK	*3*	*-0.91 [-4.42, 2.59]*	*0.61*	*0*	
Others	*4*	*-0.95 [-2.90, 1.01]*	*0.34*	*0*	
Age group					
Adults	*8*	*-0.27 [-1.53, 0.99]*	*0.68*	*0*	*0.66*
Adolescents and children	*2*	*-0.65 [-3.24, 1.94]*	*0.63*	*0*	
Device					
CamAPX FX	*4*	*-0.28 [-3.15, 2.58]*	*0.85*	*0*	*0.57*
Medtronic 670G	*2*	*1.09 [-0.98, 3.15]*	*0.3*	*36*	
Medtronic 780G	*2*	*-1.33 [-4.20, 1.54]*	*0.36*	*0*	
Let bionic pancreas	*1*	*-1.00 [-3.25, 1.25]*	*0.38*	*NA*	
Others	*1*	*-1.26 [-4.42, 1.90]*	*0.43*	*NA*	
Insulin					
Fisap	*8*	*-0.95 [-2.32, 0.41]*	*0.17*	*0*	*0.11*

URLi	*2*	*1.04 [-1.01, 3.08]*	*0.32*	*42*	
Study duratioin					
Long	*8*	*-0.27 [-1.42, 0.88]*	*0.65*	*0*	*0.44*
short	*2*	*-3.15 [-10.36, 4.06]*	*0.39*	*0*	
Nighttime data subgroups					
Country					
USA	*2*	*2.32 [1.78, 2.87]*	*0.0001*	*56*	*0.07*
UK	*2*	*-1.65 [-5.62, 2.32]*	*0.42*	*0*	
Others	*3*	*0.38 [-2.59, 3.34]*	*0.8*	*0*	
Age group					
Adults	*5*	*2.25 [1.71, 2.79]*	*0.0001*	*41*	*0.18*
Adolescents and children	*2*	*0.13 [-2.95, 3.21]*	*0.93*	*0*	
Device					
CamAPX FX	*3*	*-0.81 [-4.09, 2.47]*	*0.63*	*0*	*0.07*
Medtronic 670G	*1*	*2.40 [1.84, 2.96]*	*0.0001*	*NA*	
Medtronic 780G	*2*	*0.16 [-3.28, 3.60]*	*0.93*	*0*	
Let bionic pancreas	*1*	*0.00 [-3.05, 3.05]*	*1*	*NA*	
Insulin					
Fisap	*5*	*0.23 [-1.78, 2.23]*	*0.82*	*0*	*0.05*
URLi	*2*	*2.34 [1.78, 2.89]*	*0.0001*	*77*	
Study duratioin					
Long	*6*	*2.19 [1.66, 2.72]*	*0.0001*	*42*	*0.99*
short	*1*	*2.08 [-10.37, 14.53]*	*0.74*	*NA*	
Daytime data subgroups					
Country					
USA	*2*	*-0.18 [-3.40, 3.04]*	*0.91*	*89*	*0.97*
UK	*2*	*-0.75 [-4.54, 3.04]*	*0.7*	*0*	
Others	*2*	*-0.66 [-3.55, 2.22]*	*0.65*	*0*	
Age group					
Adults	*4*	*-0.32 [-2.66, 2.02]*	*0.79*	*71*	*0.88*
Adolescents and children	*2*	*-0.61 [-3.53, 2.30]*	*0.68*	*0*	
Device					
CamAPX FX	*3*	*-0.48 [-3.50, 2.54]*	*0.76*	*0*	*0.01*

Medtronic 670G	*1*	*1.30 [0.87, 1.73]*	*0.0001*	*NA*	
Medtronic 780G	*1*	*-1.00 [-4.54, 2.54]*	*0.58*	*NA*	
Let bionic pancreas	*1*	*-2.00 [-4.12, 0.12]*	*0.06*	*NA*	
Insulin					
Fisap	*4*	*-1.36 [-2.98, 0.26]*	*0.1*	*0*	*0.06*
URLi	*2*	*0.98 [-0.90, 2.86]*	*0.31*	*19*	
insulin TDD					
24 hour data subgroups					
Country					
USA	*3*	*0.50 [-0.06, 1.06]*	*0.08*	*0*	*0.91*
UK	*2*	*0.56 [-5.68, 6.79]*	*0.86*	*0*	
Others	*3*	*1.50 [-3.09, 6.09]*	*0.52*	*0*	
Device					
CamAPX FX	*3*	*0.36 [-4.64, 5.35]*	*0.89*	*0*	*0.95*
Medtronic 670G	*3*	*0.50 [-0.06, 1.06]*	*0.08*	*0*	
Medtronic 780G	*1*	*2.50 [-5.93, 10.93]*	*0.56*	*NA*	
Others	*1*	*1.90 [-5.35, 9.15]*	*0.61*	*NA*	
Insulin					
Fisap	*6*	*1.26 [-2.47, 4.98]*	*0.51*	*0*	*0.69*
URLi	*2*	*0.50 [-0.06, 1.06]*	*0.08*	*0*	
Study duratioin					
Long	*5*	*0.50 [-0.06, 1.05]*	*0.08*	*0*	*0.62*
short	*3*	*1.69 [-2.91, 6.29]*	*0.47*	*0*	
